# *TGTT* and *AACA*: two transcriptionally active LTR retrotransposon subfamilies with a specific LTR structure and horizontal transfer in four Rosaceae species

**DOI:** 10.1186/s13100-017-0098-8

**Published:** 2017-10-27

**Authors:** Hao Yin, Xiao Wu, Dongqing Shi, Yangyang Chen, Kaijie Qi, Zhengqiang Ma, Shaoling Zhang

**Affiliations:** 1 Center of Pear Engineering Technology Research, College of Horticulture, Nanjing Agricultural University, Nanjing, 210095 China; 2 State Key Laboratory of Crop Genetics and Germplasm Enhancement, Nanjing Agricultural University, Nanjing, China; 3 College of Agricultural Sciences, Nanjing Agricultural University, Nanjing, China

**Keywords:** LTR retrotransposon, Horizontal transfer, Transcription activity, Pear, Rosaceae

## Abstract

**Background:**

Long terminal repeat retrotransposons (LTR-RTs) are major components of plant genomes. Common LTR-RTs contain the palindromic dinucleotide 5′-‘TG’–‘CA’-3′ motif at the ends. Thus, further analyses of non-canonical LTR-RTs with non-palindromic motifs will enhance our understanding of their structures and evolutionary history.

**Results:**

Here, we report two new LTR-RT subfamilies (*TGTT* and *AACA*) with atypical dinucleotide ends of 5′-‘TG’–‘TT’-3′, and 5′-‘AA’–‘CA’-3′ in pear, apple, peach and mei. In total, 91 intact LTR-RTs were identified and classified into four *TGTT* and four *AACA* families. A structural annotation analysis showed that the four *TGTT* families, together with *AACA1* and *AACA2*, belong to the *Copia*-like superfamily, whereas *AACA3* and *AACA4* appeared to be TRIM elements. The average amplification time frames for the eight families ranged from 0.05 to 2.32 million years. Phylogenetics coupled with sequence analyses revealed that the *TGTT1* elements of peach were horizontally transferred from apple. In addition, 32 elements from two *TGTT* and three *AACA* families had detectable transcriptional activation, and a qRT-PCR analysis indicated that their expression levels varied dramatically in different species, organs and stress treatments.

**Conclusions:**

Two novel LTR-RT subfamilies that terminated with non-palindromic dinucleotides at the ends of their LTRs were identified in four Rosaceae species, and a deep analysis showed their recent activity, horizontal transfer and varied transcriptional levels in different species, organs and stress treatments. This work enhances our understanding of the structural variation and evolutionary history of LTR-RTs in plants and also provides a valuable resource for future investigations of LTR-RTs having specific structures in other species.

**Electronic supplementary material:**

The online version of this article (10.1186/s13100-017-0098-8) contains supplementary material, which is available to authorized users.

## Background

Long terminal repeat retrotransposons (LTR-RTs) are major components that are widespread in flower plant genomes [1]. They are capable of propagating to reach thousands of copies in a genome using RNA as an intermediate [2, 3]. LTR-RTs are the most significant contributor to genome size, representing 43% of the nuclear DNA in pear [4], 38% in apple [5], 19% in peach [6], 53% in cotton [7] and over 70% in maize genomes [8]. A representative intact LTR-RT usually contains two highly identical LTRs, which are typically flanked by 2-bp palindromic motifs, commonly 5′-TG–CA-3′. The internal region of an autonomous LTR should contain a primer-binding site (PBS), a polypurine tract (PPT) and two functional genes (*gag*, and *pol*) [9]. Based on the order of Reverse transcriptase (*rt*) and Integrase (*int*) in *pol*, LTR-RTs can be further classed into *Gypsy* and *Copia* super-families [9]. In addition, the LTR-RTs also contain two types of non-autonomous groups, large retrotransposon derivatives (LARDs) [10] and terminal-repeat retrotransposons in miniature (TRIMs) [11]. The insertion of an LTR-RT is accompanied by the duplication of a 4–6-bp sequence immediately flanking with the 5′ and 3′ ends of the element, called target site duplication (TSD).

The most common dinucleotide motif flanking the direct LTR-RT repeat regions is the palindromic 5′-TG–CA-3′ motif. However, several LTR-RT families with non-TGCA motifs have been reported. For example, *Tos17*, a rice LTR-RT that can be activated by tissue culture, has a non-canonical motif of 5′-TG–GA-3′ [12] and *TARE1*, which was identified as intensively amplified in the tomato genome, ends with 5′-TA–CA-3′ motifs [13]. In addition, *AcCOPIA1* that terminated with 5′-‘TG’–‘TA’-3′ at both ends of the LTRs was identified in onion [14]. However, no such non-canonical elements have been identified in the Rosaceae species.

Horizontal transfers (HTs) indicate the transmission of genetic material among sexually isolated species. As a possible dissemination mechanism of transposable elements (TEs) in eukaryotes, the horizontal transfer of TEs (HTTs) into a new organism is an important step for the TE to escape from the silencing machinery of their host genome and obtain a new ‘life cycle’ [15]. The first case of horizontal TE transfer (HTT) was the *P* TE identified between *Drosophila willistoni* and *Drosophila melanogaster* [16]. Recently, with the availability of many plant genome sequences, several HTT cases have been reported mainly through comparative genomic approaches. For example, multiple HTs of the LTR-RT *RIRE1* were identified within the genus *Oryza* [17], and another LTR-RT family *Route66* were found and proven to be HTs among the rice, maize and sorghum genomes through a comparative genomics analysis [18]. In addition, 32 HTs of LTR-RTs were discovered by whole genome surveys and comparative analyses in 46 sequenced plant genomes [19].

The propensity of LTR-RTs not only contributed to genome size but also resulted in byproducts of gene disruption, expression level alterations and genomic rearrangements by inserting themselves into genes or their promoter regions [20, 21]. In plants, LTR-RTs are usually silent under normal conditions, but some show transcriptional activities and increased accumulations while under stress, potentially triggering the genetic diversity required to evolve adaptations [21, 22]. For example, salt (*AtCopeg1* in *Arabidopsis* [23]), drought (*BARE1* in barley [24]), heat (*ONSEN* in *Arabidopsis* [25, 26]), cold (*mPing* in rice [27, 28]) and wounding (*Corky* from Quercus [29]; *CLCoy1* in lemon [30], *OARE1* in oat [31] and *Tnt1* in tobacco [32]). Recently, several LTR-RTs were identified as being expressed in the fruits and buds of pear in the RNA-seq databases [33]. However, their study did not focus on the LTR-RTs’ transcription activities under stress in pear.

The Rosaceae family is an economically important angiosperm lineage, containing over 3000 distinct species with chromosome’s numbering from 7 to 17 pairs [34]. Some genera with higher economic values that are widely cultivated have had their whole genomes sequenced in the last decade, including pear (*Pyrus bretschneideri*, *n* = 17, 527 Mb) [4], apple (*Malus domestica*, n = 17, 743 Mb) [5], peach (*Prunus persica*, *n* = 8, 265 Mb) [6], mei (*Prunus mume*, n = 8, 280 Mb) [35] and woodland strawberry (*Fragria vesca, n* = 7) [36] (Additional file 1: Table S1). Based on DNA sequence data, *Fragaria* belongs to the Rosoideae, supertribe Rosadea, tribe Potentilleae, *Malus* and *Pyrus* occur in the Spiraeoideae, supertribe Pyrodeae, tribe Pyreae and *Prunus* is in the Spiraeoideae, tribe Amygdaleae [37]. The availability of the five Rosaceae genomic sequences provided opportunities to undertake comparative analyses of LTR-RTs in pear and four other genomes [3, 38]. In this study, a genome wide identification of non-typical LTR-RTs in pear genome was conducted. Two new subfamilies of LTR-RTs, *TGTT* and *AACA*, were identified in pear, apple, peach and mei, but not in strawberry. Their structures, abundance levels, insertion time frames, evolution and transcription activities have been comprehensively analyzed between the four Rosaceae species. *TGTT* and *AACA* elements terminate in short inverted repeat dinucleotides, such as ‘TG’ and ‘TT’, ‘AA’ and ‘CA’, and the *AACA1* elements in peach may have been horizontally transferred from apple. In addition, multiple elements from the two subfamilies present differential expression levels in different pear organs and also show different expression levels under heat, cold and salt stress treatments. Our study reveals novel structures, horizontal transfer and the transcription activation of two new LTR-RT subfamilies, providing additional information on, and knowledge of, the structure, evolution and activity of TEs in plants.

## Results

### Identification, structural characterization and sequence analysis of *TGTT* and *AACA* TEs in the pear genome

**Fig. 1:**
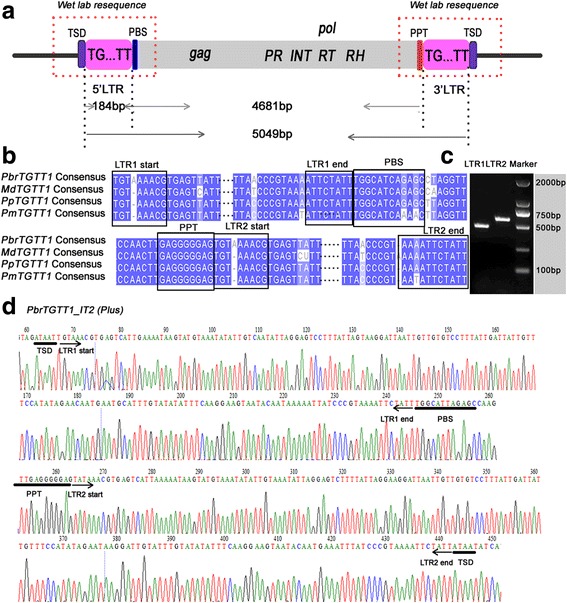
Schematic presentation, consensus sequence comparison and wet laboratory verification of *TGTT1* elements. **a** Structural annotations of the *TGTT* elements. The long terminal repeats (LTRs) are shown in pink boxes; ‘TSD’ indicates the target site duplication; ‘PBS’ indicates the primer binding site; ‘PPT’ indicates the polypurine tract; PR, INT, RT and RH are abbreviations for GAG-pre-integrase, integrase, reverse transcriptase and Ribonuclease H domains, respectively. **b** The *TGTT1* consensus sequence alignment from pear, apple, peach and mei genomes. Identical nucleotides are shown with *blue* shadows. The internal LTR sequences are marked by ellipsis. **c** PCR amplification of one randomly selected *TGTT1* element (*PbrTGTT1_IT2*) from the pear genome. The physical positions of the element are located on scaffold809.0 from 128,978 to 133,998. **d** Resequencing of the *PbrTGTT1_IT2* element

**Fig. 2:**
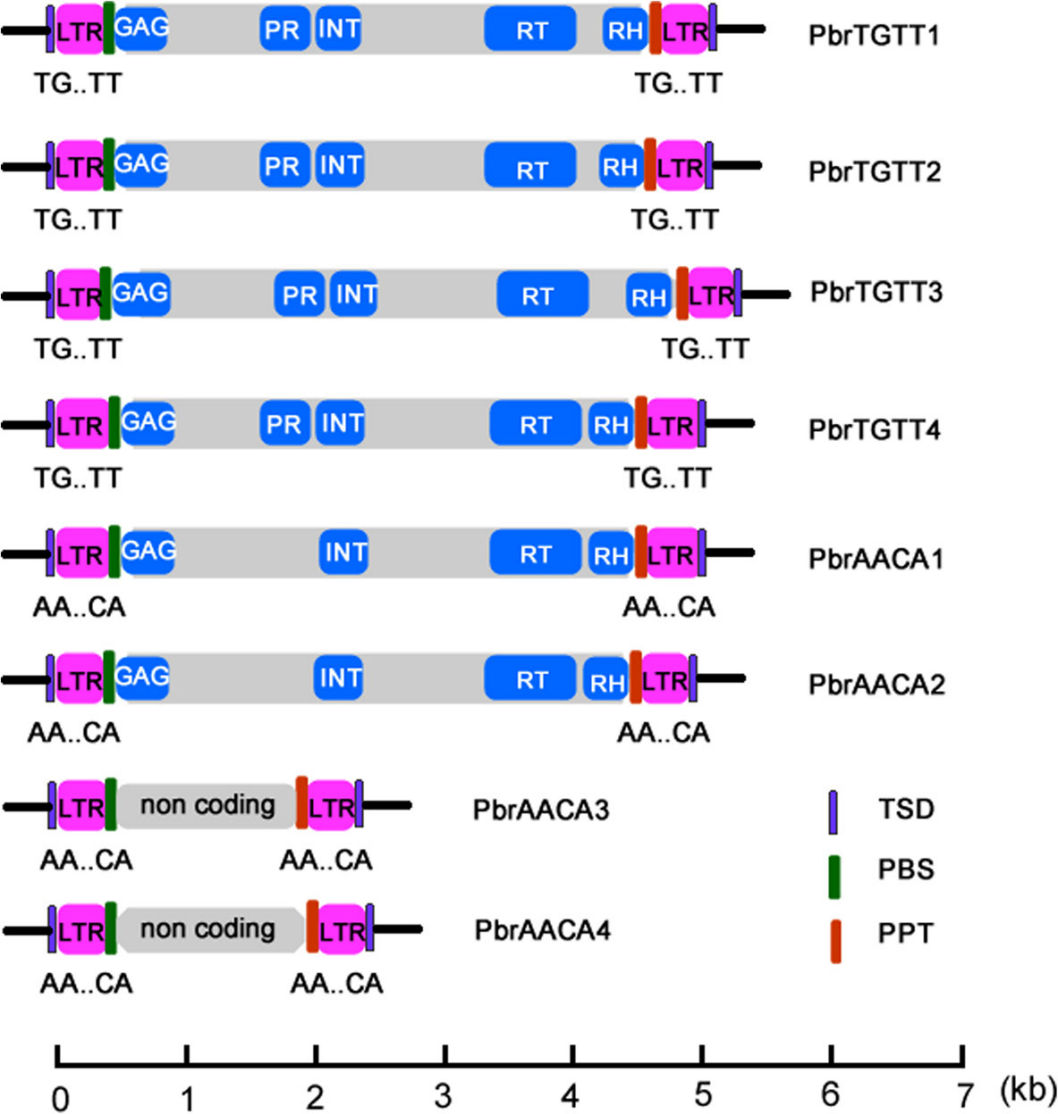
Schematic representation of *TGTT* and *AACA* structures in pear. The *black* lines at the ends represent the DNA sequences. The scale below measures the lengths of the elements

We started our analyses by focusing on a class of atypical LTR-RTs identified in the pear (*P. bretschneideri*) genome. Initially, 12 intact TEs with atypical characteristics were identified using the LTR_STRUC program [39]. The LTRs of the 12 intact TEs terminate in the dinucleotide 5′-TG–TT-3′ or 5′-AA–CA-3′ (Figs. 1a,b and 2) instead of 5′-TG–CA-3′ usually found in typical LTR-RTs. Thus, these TEs were classified into two subfamilies, named *TGTT* and *AACA*, based on their terminal dinucleotides. In total, 66 intact TEs with two clearly defined boundaries and TSDs were identified using combined homology-based approaches as previously described [3, 13, 38]. Using the unified classification for eukaryotic TEs [40], the 66 TEs were grouped into eight distinct families based on an over 80% identity in at least 80% of their LTR regions (Table 1, Additional file 1: Tables S2 and S3 and Additional file 2: Figure S1). Four families, containing 35 TEs, belonged to the *TGTT* subfamily and the other four families, containing 31 TEs, belonged to the *AACA* subfamily. We randomly selected nine elements and confirmed the existence of 5′-TG–TT-3′ and 5′-AA–CA-3′ terminals in the LTR sequences by PCR and Sanger sequencing (see Methods, Fig. 1c and d, Additional file 2: Figure S2 and Additional file 1: Table S4).

**Table 1:** Summary of *TGTT* and *AACA* elements in four Rosaceae genomes

Family	Superfamily	No. of intact element	Length of LTR (bp)	Length of intact element (bp)	Start and end of LTR (Plus)	Length of TSDs	Ave. age
	Lineage						(mys)
*PbrTGTT1*	*Copia/Ale*	8	184	5049	TG..TT	5	2.32
*MdTGTT1*	*Copia/Ale*	6	179	5113	TG..TT	5	0.91
*PpTGTT1*	*Copia/Ale*	4	179	5009	TG..TT	5	0.16
*PmTGTT1*	*Copia/ Ale*	2	180	5055	TG..TT	5	0.65
*PbrTGTT2*	*Copia/ Ale*	22	180	5039	TG..TT	5	0.3
*MdTGTT2*	*Copia/ Ale*	3	169	5046	TG..TT	5	0.92
*PbrTGTT3*	*Copia/ Ale*	2	152	5114	TG..TT	4	0.38
*PbrTGTT4*	*Copia/ Ale*	3	229	4999	TG..TT	6	0.28
Subtotal/average		50	180	5053	TG..TT	5	0.74
*PbrAACA1*	*Copia/ Ale*	7	266	4924	AA..CA	6	0.94
*PmAACA1*	*Copia/ Ale*	2	251	4883	AA..CA	6	0.15
*PbrAACA2*	*Copia/ Ale*	4	201	4857	AA..CA	6	0.05
*PbrAACA3*	*TRIM*	4	244	2129	AA..CA	6	1.85
*PbrAACA4*	*TRIM*	16	203/242	2364/2522	AA..CA	6	1.84
*MdAACA4*	*TRIM*	8	207/242	2606/2735	AA..CA	6	1.03
Subtotal/average		41	232	3378	AA..CA	6	1.26

Note: *PbrAACA4* and *MdAACA4* elements can be separated into two sub-groups based on their sequence length, since their sequence identity and sequence length are still over 80%, the two sub-groups were still classified into one family

The consensus sequence sizes of the eight families ranged from 2129 (*PbrAACA3*) to 5114 bp (*PbrTGTT3*), and the LTR sequence sizes ranged from 152 (*PbrTGTT3*) to 266 bp (*PbrAACA1*, Table 1)*.* The coding sequences of the 66 elements indicated that all of the *PbrTGTT* elements contained the *Gag* and *Pol* genes, including the protease (PR), integrase (INT), reverse transcriptase (RT), and RNase H (RH) domains. The *PbrAACA1* and *PbrAACA2* TEs also contain the *Gag* and *Pol* genes, but the PR domain was absent in their *Pol*s. The order of *int*, *rt.* and *rh* defined the six families (46 elements) as *Copia*-like elements (Fig. 2). Interestingly, no coding sequences were identified in the short internal sequences between the two LTRs (1641–2042-bp) of *PbrAACA3* and *PbrAACA4* (20 elements), indicating that these two *AACA* families were TRIM families (Fig. 2). Notably, the TSD sizes of the *PbrAACA* elements were 6 bp, while those of the *PbrTGTT* family varied from 4 to 6 bp (Table 1).

### *TGTT* and *AACA* TEs are also present in other Rosaceae genomes

To detect whether the *TGTT* and *AACA* elements are specific to the pear genome, these elements were annotated in other published plant genomes at pyhtozome (http://www.phytozome.net) using the same strategies as described above. Only four families of *TGTT* and *AACA* were identified in three other closely related Rosaceae genomes, apple (*M. domestica*) [5], peach (*P. persica*) [6] and mei (*P. mume*) [35] (Additional file 1: Table S1). To distinguish these TEs in different genomes, we have named them *MdTGTT1*, *PpTGTT1*, *PmTGTT1*, *MdTGTT2*, *PmAACA1* and *MdAACA4* (Table 1, Additional file 1: Table S3). In total, six *MdTGTT1* copies, four *PpTGTT1* copies, two *PmTGTT1* copies, three *MdTGTT2* copies, two *PmAACA1* and eight *MdAACA4* copies, which are all less than the number in pear, were identified. No *TGTT* or *AACA* TEs were identified in the closely related Rosaceae species, woodland strawberry (*F. vesca*) [36] or other published plant genomes.

### Variable spectra of activities for amplification of *TGTT* and *AACA* elements over evolutionary time

**Fig. 3:**
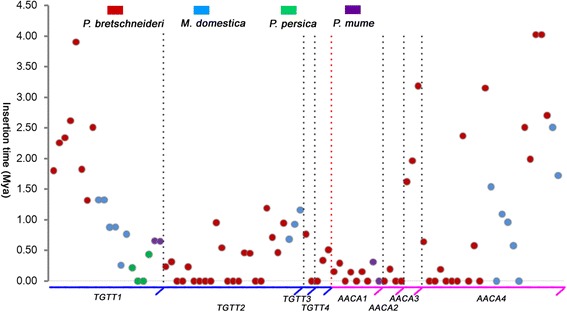
Insertion times of *TGTT* and *AACA* elements in the four Rosaceae species. The *y*-axis represents the insertion time. Each *TGTT* and *AACA* family is separated by dotted lines. Elements from different species are represented by red (*Pyrus bretschneideri*), *blue* (*Malus domestica*), green (*Prunus persica*) and purple (*Prunus mume*) circles, respectively

To compare the activities and amplification time frames of *TGTT* and *AACA* elements among the four Rosaceae species, the full-length TEs with TSDs were dated using a previously described approach [41, 42]. Even though the two LTR sequences of an intact LTR-RT element are identical at the time of insertion, both LTRs accumulate nucleotide substitutions independently over evolutionary time. Thus, when an evolutionary rate is applied to the LTR-RT element, the sequence divergence of two LTRs can be roughly converted into the insertion time. Although the evolution rate of LTR-RTs varies among different loci, families, and lineages [43], an estimation of 1.3 × 10^−8^ per site per year has been applied in many studies [13, 42, 44]. Using this rate, the insertion times of the 50 *TGTT* and 41 *AACA* intact copies with TSDs from the four Rosaceae species were estimated. The following was observed: 1) the average insertion times of *TGTT* and *AACA* subfamilies are 0.74 and 1.26 million years (Mys), respectively; 2) the average insertion times of the eight families in the four Rosaceae species ranged from 0.05 (*PbrAACA2*) to 2.32 Mys (*PbrTGTT1*). Most of these elements (65, 71.43%) inserted into the genome <1.0 million years ago (Mya), and 21 copies (23.08%) integrated into the genome within 1–3 Mya. In addition, only five copies (5.49%) have been dated >3 Mya; 3) over one third of these *TGTT* and *AACA* elements (31, 34.07%) have two identical LTRs, and the ratio of *TGTT* to *AACA* TEs is almost 1:1 (15:16, Fig. 3, Additional file 1: Table S3); and 4) the average insertion times of *TGTT* and *AACA* TEs varied among pear, apple, peach and mei, at 1.00, 0.95, 0.16 and 0.40 Mya, respectively (Fig. 3).

### The evolutionary relationship between *TGTT* and *AACA* TEs

**Fig. 4:**
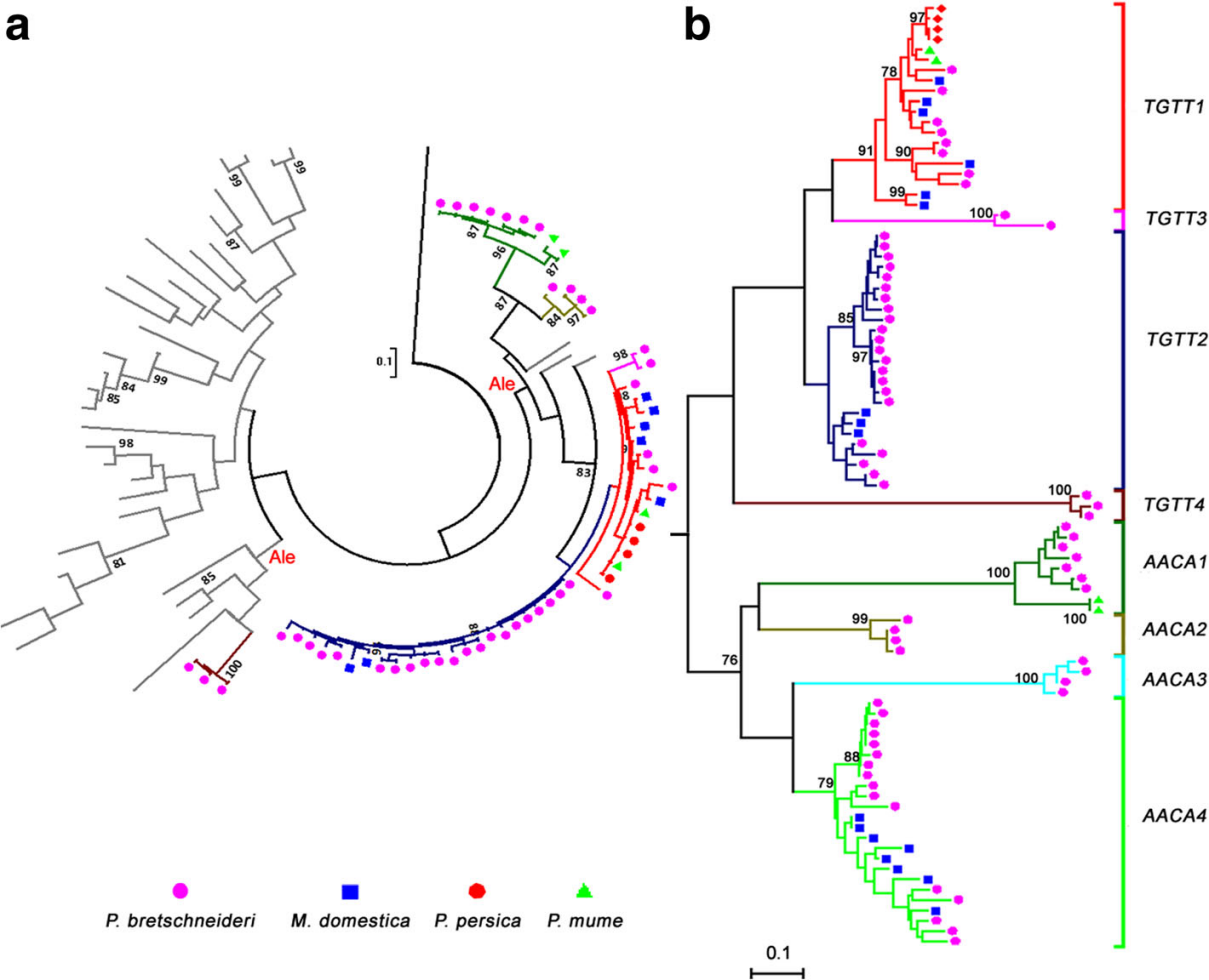
Phylogenetic relationships of *TGTT* and *AACA* elements. **a** RT phylogenetic relationship of six *Copia*-like *TGTT* and *AACA* families identified in four Rosaceae species. A *Bel-Pao* type RT (gi#972521 from GenBank) of *Bombyx mori* was used as an outgroup. The lineage reference sequences described by lineage names are available in the Repbase database [42]. **b** LTR-based phylogenetic relationships of eight *TGTT* and *AACA* families identified in four Rosaceae species. The 5′-LTR sequences of 91 *TGTT* and *AACA* elements were extracted from *Pyrus bretschneideri* (*pink circles*), *Malus domestica* (*blue squares*), *Prunus persica* (*red circles*) and *Prunus mume* (*green triangles*). The families are indicated by various branch colors. The nucleotide sequence distances are indicated by the scales

To understand the evolutionary relationships among *TGTT* and *AACA* TEs in the four Rosaceae species, a phylogenetic tree using the 5′ LTR sequences was constructed (Fig. 4a). The 91 *TGTT* and *AACA* TEs can be successfully separated into eight clades. The four *TGTT* families clustered together, and the four *AACA* families were closer to each other, indicating that the *TGTT* and *AACA* TEs evolved independently. Although *TGTT* and *AACA* TEs are separated from each other, the *MdTGTT2* and *MdAACA4* elements were mixed with *PbrTGTT2* and *PbrAACA4* elements, respectively. In addition, the *PpTGTT1* and *PmTGTT1* were also found with *MdTGTT1* and *PbrTGTT1*, indicating that the species may have experienced some introgression in early stages of their evolution or HT events after their divergence.

The individual *Copia*-like LTR-RT families can be separated into six major evolutionary lineages, *Angela*, *Ale*, *Bianca*, *Ivana*, *Maximus* and *TAR*. To discern the evolutionary history and phylogenetic relationships among the four *TGTT* and two *AACA Copia-*like families and the major evolutionary lineages, the conserved RT DNA sequences from each of the *TGTT* and *AACA* elements, as well as *Copia*-like LTR-RTs in *Arabidopsis*, rice and soybean, which were previously identified [42], were used to construct a Maximum Likelihood (ML) phylogenetic tree. As shown in Fig. 4a, the six *Copia*-like *TGTT* and *AACA* families all belong to the *Ale* lineage but formed three distinct sublineages. The two *AACA* families were separated into a sublineage, three *TGTT* families (*TGTT1–3*) formed another sublineage, with two sublineages being closer to soybean *Ale* elements, while the *TGTT4* elements, together with the *Arabidopsis Ale* elements, grouped into a distinct sublineage. Because the two *AACA* TRIM families have no coding genes inside the internal regions, their PBS sites were used to make multiple alignments with those of other elements. Interestingly, the PBS sites were highly conserved with those of other *Ale* lineage elements (Additional file 2: Figure S3). Thus, the two *AACA* TRIM families may also originate from the *Ale* lineage.

### HT of *TGTT1* elements between apple and peach genomes

**Fig. 5:**
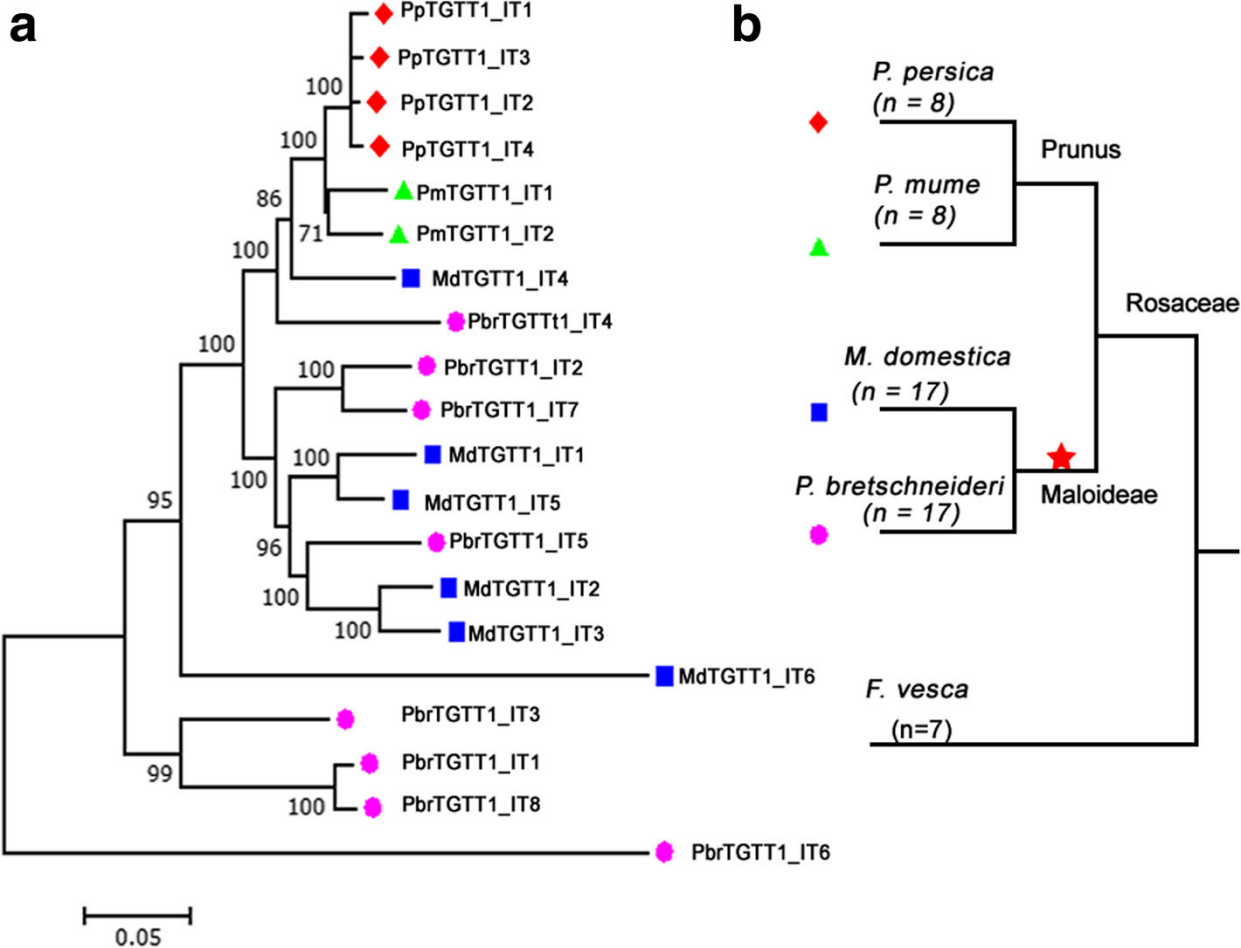
Phylogenetic incongruences between horizontally transferred *TGTT1* elements and trees of four Rosaceae species. **a** *TGTT1* tree; the ML phylogenetic tree was based on intact sequences of 20 *TGTT1* elements from 8 *PbrTGTT1* (*pink circles*), 6 *MdTGTT1* (*blue squares*), 4 *PpTGTT1* (*red circles*) and 2 *PmTGTT1* (*green triangles*). **b** Species tree; The red star indicates the recent whole-genome duplication event. The nucleotide sequence distances are indicated by the scales

*TGTT1* is the only family identified in all four Rosaceae species (pear, apple, peach and mei) but not in the woodland strawberry and other species, indicating that these TEs arose after the divergence of strawberry (*F. vesca*, *n* = 7) and the ancestors of pear (*P. bretschneideri*, *n* = 17), apple (*M. domestica*, n = 17), peach (*P. persica*, *n* = 8) and mei (*P. mume*, n = 8). Previously, the 20 *TGTT1* elements from four Rosaceae species were shown to be mixed in an ML phylogenetic tree based on LTR sequences (Fig. 4b). To understand the evolutionary history of the *TGTT1* elements, the ML phylogenetic tree of *TGTT1* was rebuilt using the whole complete sequences of the 20 TEs, and the data indicated that the *TGTT1* TEs had a patchy distribution in the phylogenies (Fig. 5a). Specifically, four *PpTGTT1* and two *PmTGTT1* TEs were phylogenetically closer to the apple *TGTT1* element (*MdTGTT1_IT4*). The phylogenetic relationships among these *TGTT1* TEs were not fully congruent with their host species (Fig. 5b), which prompted an investigation into the possibility of HT occurring between distantly related Prunus (peach and mei) and Maloideae (apple and pear) species.

**Fig. 6:**
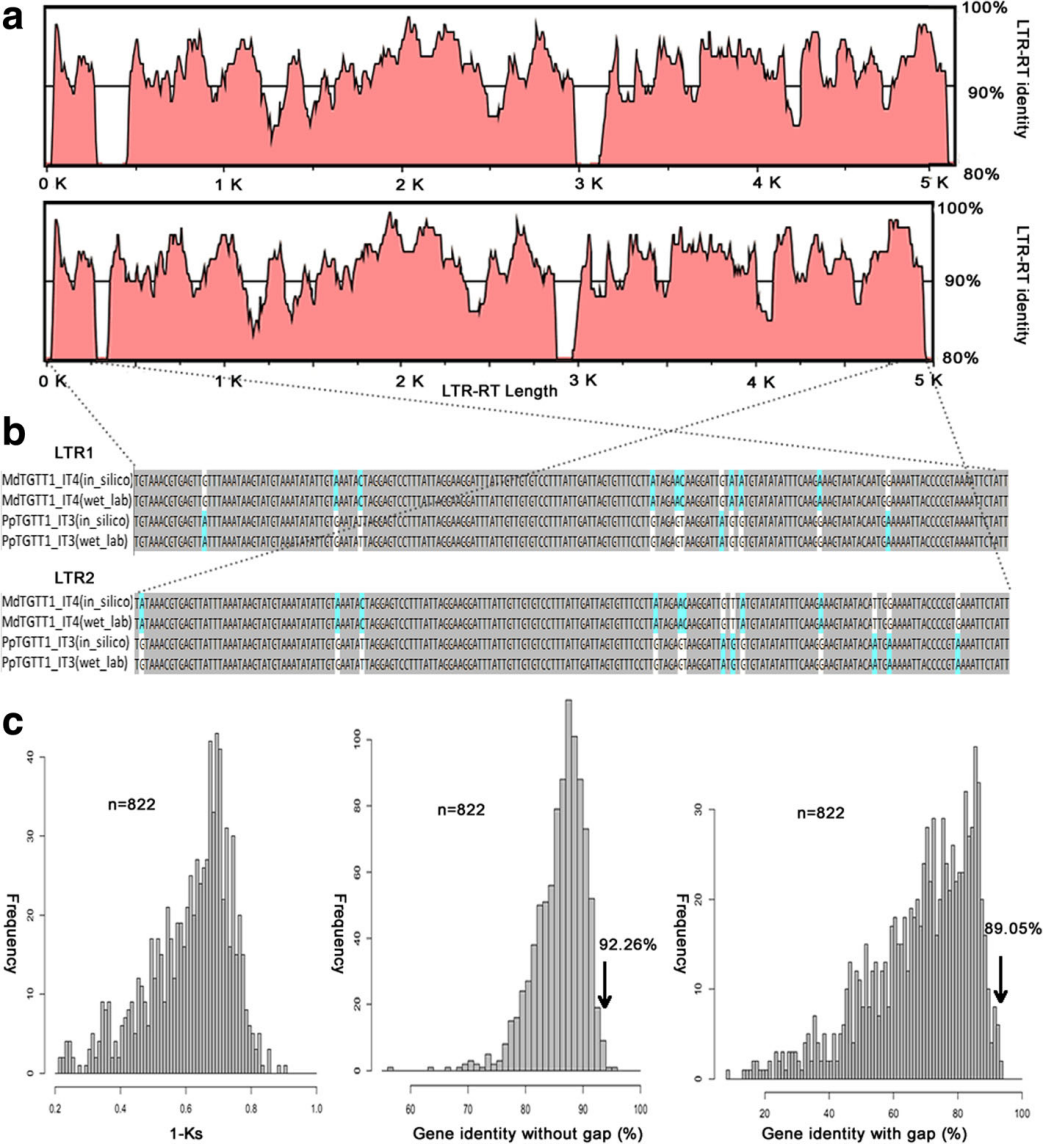
Comparisons between the sequence identities of HT LTR-RTs and the genomic distances between the two host species involved in the HT. **a** The sequence identities along the complete lengths of the HT LTR-RTs. The *black* line represents the 90% identity threshold. **b** Wet laboratory validation of the HTTs. Sequenced PCR products of LTRs were aligned with the sequences that were mined from the genome sequence. **c** Histogram representing the distribution of orthologous single-gene identities based on Ks analyses and CDS comparisons with or without gaps (see Methods). The numbers of CDS pairs of orthologous single genes analyzed are as indicated (n). Arrows correspond to average sequence identities between the HT LTR-RTs

To test this hypothesis, the sequence identities between pairs of *TGTT1* elements were initially analyzed. A higher sequence similarity (92.26%, gaps were excluded) was identified between peach (*PpTGTT1_IT3*) and apple *(MdTGTT1_IT4*), and even when taking into account the gaps between the two sequences, the sequence similarity was 89.05% (Additional file 1: Table S5). Additionally, to make a comparison, sequence identities of single orthologous genes were calculated between peach and apple based on their synonymous substitutions per site (Ks; average = 61.29% ± 12.85%) and sequence similarities (gaps calculated average = 69.80% ± 15.56%; gaps excluded average = 86.57% ± 3.59%). Sequence identity at the peak of the distribution should be a good indicator of overall genomic divergence [19]. Here, the sequence identities of *PpTGTT1_IT3* and *MdTGTT1_IT4* were always higher than the sequence identity peak values of 822 orthologous single genes (Fig. 6, Additional file 1: Tables S6 and S7). This hypothesis was also supported by the Ks values of *TGTT1* family integrases, which are much lower for *PpTGTT1* (0.02 ± 0.01) than for *MdTGTT1* (0.78 ± 0.29) (Table 2), suggesting that it recently entered the peach genome. The presence of the two elements with higher sequence identities in apple and peach were tested by PCR amplification of the LTRs and Sanger sequencing (Fig. 6 and Additional file 2: Figure S2). Thus, the HT of *TGTT1* might have occurred between the distantly related apple and peach.

**Table 2:** Ka, Ks and Ka/Ks values of integrases in the *TGTT* and *AACA* families

Family	Ka	Ks	Ka/Ks
*PbrTGTT1*	0.73 ± 0.42	0.56 ± 0.18	1.33 ± 0.65
*MdTGTT1*	0.74 ± 0.39	0.78 ± 0.29	0.99 ± 0.48
*PpTGTT1*	0.01 ± 0.00	0.02 ± 0.01	0.51 ± 0.36
*PmTGTT1*	0.51 ± 0.00	0.50 ± 0.00	1.01 ± 0.00
*PbrTGTT2*	0.15 ± 0.17	0.32 ± 0.21	0.42 ± 0.38
*MdTGTT2*	0.42 ± 0.24	0.44 ± 0.20	0.85 ± 0.24
*PbrTGTT3*	0.12 ± 0.00	0.09 ± 0.00	1.25 ± 0.00
*PbrTGTT4*	0.58 ± 0.00	0.61 ± 0.00	0.96 ± 0.00
*PbrAACA1*	0.49 ± 0.73	0.33 ± 0.42	0.63 ± 0.67
*PmAACA1*	0.00 ± 0.00	0.01 ± 0.00	0.29 ± 0.00
*PbrAACA2*	0.03 ± 0.00	0.11 ± 0.00	0.25 ± 0.00

### Transcriptional activities of *TGTT* and *AACA* TEs in different organs and under stress treatments in pear

**Fig. 7:**
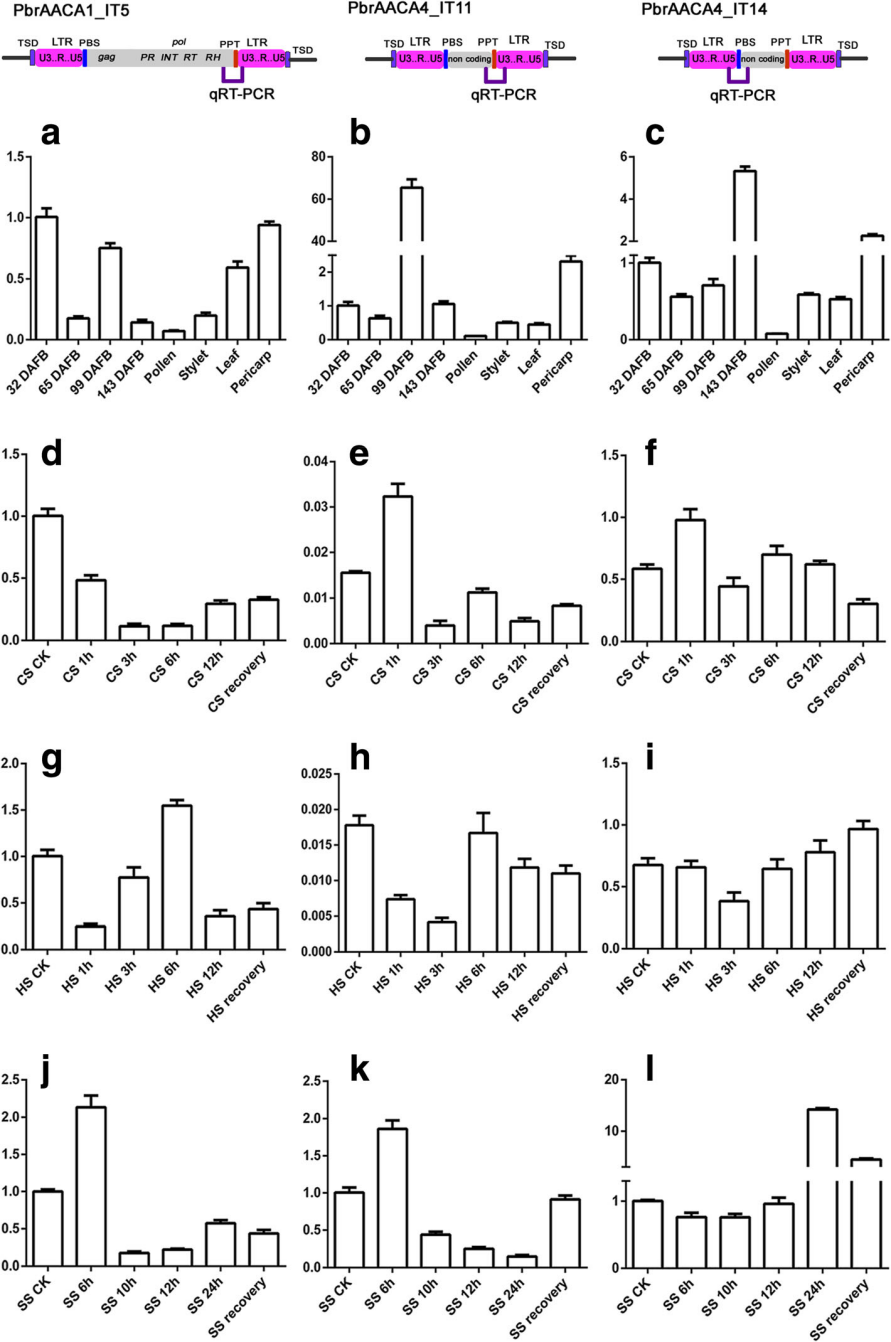
Time-course expression levels of active *AACA* elements in *Pyrus bretschneideri*. The positions of the primers used for transcriptional validation are indicated in the schematic of each element in qRT-PCR region. The expression levels were detected in the fruit flesh of four developmental stages, pollen, stylet, leaf and pericarp. **a–c**, leaves under cold (**d–f**), heat (**g–i**), and salt (**j–l**) treatments for *PbrAACA1_IT5* (**a, d, g, j**), *PbrAACA4_IT11* (**b, e, h, k**) and *PbrAACA4_IT14* (**c, f, i, l**). Error bars indicate the standard deviations of three biological replicates

Because over 70% of *TGTT* and *AACA* TEs were inserted into the four genomes <1.0 Mya, and over one third of these elements (34.07%) contained two identical LTRs, these TEs may still be transcriptionally or even transpositionally active. To detect the transcriptional activity of *TGTT* and *AACA* TEs in the four Rosaceae species, HISAT alignments for each were constructed using the RNA-seq data from Sequence Read Archive (SRA) database of NCBI. A total of 12 *AACA* TEs from pear, including 7 *PbrAACA1*, 1 *PbrAACA3* and 4 *PbrAACA4*, were transcriptionally active in fruit and buds of *P. bretshneideri* (‘Dangshansuli’; Additional file 1: Table S3 and Additional file 2: Figure S5), whereas none of the *TGTT* TEs were active according to the RNA-seq data. To gain insight into the expression levels of these active TEs, three pair of primers (Additional file 1: Table S8) that corresponded to the transcribed U5 of the 5′ LTR or U3 of the 3′ LTR and the partial internal regions of three randomly selected elements (*PbrAACA1_IT5*, *PbrAACA4_IT11* and *PbrAACA4_IT14*) were used for qRT-PCR in different pear (‘Dangshansuli’) organs, including fruit flesh at four developmental periods (32, 65, 99 and 143 d after flower bloom), leaves, pericarp, pollen and stylet. Transcripts of the three elements could be detected in all eight samples (Fig. 7a–c, Additional file 2: Figure S5). The transcriptional levels varied for the three elements in the different organs. For example, the transcriptional levels of *PbrAACA1_IT5* (Fig. 7a), *PbrAACA4_IT11* (Fig. 7b) and *PbrAACA4_IT14* (Fig. 7c) were highest at 32, 99 and 143 d after flower bloom in fruit flesh, respectively, and they all expressed at their lowest levels in the pollen.

The transcriptional activities of LTR-RTs are usually repressed in plant tissues during normal development, as well as in response to a variety of biotic and abiotic stresses [45]. Thus, the expression patterns of the former three elements in leaves of pear (‘Dangshansuli’) were examined under various stress treatments, including cold, heat and salt (see Methods). *PbrAACA1_IT5* was significantly up-regulated in heat (6 h, Fig. 7g) and salt (6 h, Fig. 7j) stress treatments (two-tailed *t*-test, *P* < 0.01), but down-regulated in the cold stress treatments (two-tailed *t*-test, *P* < 0.01, Fig. 7d). Elevated levels of *PbrAACA4_IT11* transcripts were observed in cold (1 h, Fig. 7e) and salt (6 h, Fig. 7k) stress treatments (two-tailed *t*-test, *P* < 0.01) but not in heat. For *PbrAACA4_IT14*, the up-regulation was also significant in cold (two-tailed *t*-test, *P* < 0.01, 1 h, Fig. 7f) and salt stress treatments (two-tailed *t*-test, *P* < 0.01, 24 h, Fig. 7l). These data may indicate the constitutive expression of *TGTT* and *AACA* TEs in different pear tissues and the elevation of their expression levels under various stresses.

## Discussion

We have isolated two novel LTR-RT subfamilies called *TGTT* and *AACA* in four Rosaceae genomes, and these can be classified into eight families according to the TE classification system proposed by Wicker et al. 2007 [40]. Six *Copia*-like families were classified into the *Ale* lineage using a phylogenetic analysis (Fig. 4a), while the other two *AACA* families were *TRIM* elements owing to their short non-coding internal regions (Fig. 2). However, *TGTT* and *AACA* TEs were restricted to four Rosacese species (pear, apple, peach and mei), and not even truncated fragments were detected in the closest woodland strawberry genome and other published plant genomes. This suggested that the two subfamilies evolved specifically after the divergence between *F. vesca* and the ancestor of the four Rosacese species.

### Specific dinucleotide termini of two new subfamilies of non-canonical LTR-RTs in the four Rosaceae species

LTR sequences start with ‘TG’ and end with ‘CA’ in typical LTR-RTs [9]. In a previous study, our group described a systematic survey of LTR-RTs in the sequenced pear (*P. bretschneideri*) genome, and 3221 full-length LTR-RTs have been found to terminate with 5′-TG–CA-3′ [3, 38]. Here, although these new TEs contained most of the typical features of LTR-RTs, a salient difference was identified in the dinucleotide positions at both ends of the LTRs. When these TEs with their two flanking sequences (500-bp for each site) where combined and aligned, they showed an accurate insertion site terminating with 5′-TG–TT-3′ or 5′-AA–CA-3′, and flanked by perfect 4 to 6-bp TSDs. Thus, these atypical LTR-RTs were defined as two new subfamilies (*TGTT* and *AACA*). Previously, an atypical LTR-RT family (*TARE1*) in tomato [13] that contained 5′-TA–CA-3′ at both ends of the LTRs was reported, and a plausible mutation model explaining the creation of such atypical dinucleotides in the LTRs was proposed. In addition, another exception, *AcCOPIA1*, which terminates with ‘TG’ and ‘TA’ at both ends of the LTRs, was identified in onion [14]. Similar to *TARE1*, only one nucleotide changed from ‘CA’ to ‘TA’ in *AcCOPIA1*. Compared with the *TARE1* and *AcCOPIA1* TEs*,* the ‘CA’ has been changed to ‘TT’ in the *TGTT* elements and ‘TG’ turned into ‘AA’ in the *AACA* elements. Interestingly, the 5′-TG–TT-'3′ of *TGTT* is the reverse complement of the 5′-AA–CA-3′ of *AACA.* The simultaneous mutation of the dinucleotides is a low probability event that cannot be easily explained by the mutation model [13]. A global annotation, structural analysis and phylogenetic study of all the non-TGCA TEs within the eukaryotes is worth performing in the future, to unravel the scale and frequencies of non-canonical LTR-RTs that terminate with non-TGCA motifs and whether they exist naturally or derived from the normal TGCA-containing LTR-RT elements.

Although most of LTR-RTs carry the palindromic dinucleotide motif (5′-TG–CA-3′) flanking each LTR, the importance of this conserved motif is still poorly understood. Studies of retrovirus integration indicate that the 3′ CA terminal sequences of retroviral LTRs are essential for viral integration [46, 47]. The close relationship between retroviruses and LTR-RT TEs, with an additional envelope protein [46, 47], may explain why most LTR-RTs have the conserved 5′-TG–CA-3′ motif. Here, based on the phylogenetic tree of *TGTT*, *AACA* and typical TGCA integrases, most *TGTT* integrases (*TGTT1*, *TGTT2* and *TGTT3* elements) can be differentiated from typical integrases (Additional file 2: Figure S4), whereas the *AACA* elements could not be differentiated. The Ka/Ks values of the INT domains of *TGTT1* and *TGTT3* families (>1) are significantly greater than those of *AACA1* and *AACA2* families (<1) (Table 2). Thus, the functional divergence of the integrase active sites from these *3′-TT ends* LTR-RTs has occurred and might result in a novel integration mechanism. Based on sequence comparisons, structural and phylogenetic analyses, the newly identified *TGTT* and *AACA* TEs should provide a valuable resource to study the non-canonical LTR-RTs integration mechanism.

### The *PpTGTT1* elements originated from *MdTGTT1* through HT

The transmission of genetic materials among sexually isolated species is usually defined as HTs. HTTs were first proposed as a possible dissemination mechanism of TEs in eukaryotes [15]. Because TEs could undergo epigenetic-mediated silencing by the host genome [48], HTTs could be the mechanism of escaping the silencing and ensuring the long-term survival of TEs among eukaryotic lineages. Based on this model, most of the active TEs found in plant and animal genomes may originate from other species through HTT [49]. Owing to the availability of more released eukaryotic genome sequences and standard comparative genomics approaches [50], hundreds of cases of HTTs have been reported over the past years [18, 19, 51]. Recently, Fawcet and Innan (2016) [52] proposed a method to differentiate the HTT and vertical transmission scenarios, which involves testing whether the hypothetical HTT copies are present in the orthologous regions of the two species [52]. If the two species acquired the copies independently by HTs, then the two species should not share any copies and each copy should be species-specific. This theory is reasonable because TE-based recombination and loss occur frequently in host genomes. The same analysis was conducted for the *TGTT1* TEs among the four Rosaceae genomes, however, no shared intact *TGTT1* TEs or degenerated fragments were detected between pairs of the Rosaceae genomes. Combined with the high similarity between *MdTGTT1_IT4* and *PpTGTT1_IT3,* the deep divergence time between Maloideae and Prunus (>45 Mys), and their patchy distribution in Rosaceae, indicates that the HT of the *TGTT1* TEs occurred between apple and peach. Recently, 32 clear cases of recent HTTs of LTR-RTs, including 5 HTTs between apple and peach, were detected among 46 sequenced plant genomes [19]. As expected, the HTT of *TGTT1* TEs was not included in the five reported HTTs between apple and peach, possibly because the *TGTT1* TEs were not identified initially through their LTR-RTs annotation method. Thus, the estimation of millions of HTTs occurring among the angiosperms in the recent evolutionary past may have been an underestimation.

The insertion time of the HTT elements have facilitated us to speculate the HTT history and time frame. Based on the sequence divergence of two LTRs of *MdTGTT1_IT4* and *PpTGTT1_IT3*, we propose that the presence of *TGTT1* in peach was resulted from HT of *MdTGTT1* between 0.43 Mys and 0.88 Mys. First, the average insertion time of the four *PpTGTT1* elements (0.16 Mys) is much younger than that of the six *MdTGTT1* elements (0.91 Mys, Additional file 1: Table S3), especially, *PpTGTT1_IT3* (0.43 Mys) aged much younger than *MdTGTT1_IT4* (0.88 Mys); Second, *PpTGTT1_IT3* is the oldest element of the four *PpTGTT1* elements (Additional file 1: Table S3); Third, four *MdTGTT1* elements including *MdTGTT1_IT4* are still transcriptionally active (Additional file 2: Figure S5 and Additional file 1: Table S3). Last, the cluster of the horizontally transferred *PpTGTT1* copies is included in the larger cluster of copies from apple and pear. All of these evidences suggest that peach is the recipient species of the HT event. Although several studies suggest that “host-vector species” interactions may favor HTTs in animals [53, 54]. However, evidence of “host-vector-driven” HTTs has not been provided in plants, and no experimental evidence of this process has been reported yet. Thus it is unclear how the transfer of *TGTT1* may have occurred between apple and peach. Considering that apple and peach belong to Rosaceae fruit crops with higher economic values, the most plausible explanation is that the *TGTT1* was transmitted by their common pathogen, such as bacteria, fungi and virus often believed to be the vectors of HT [55, 56], and perhaps with the help of a plant cell-piercing insect [54, 55]. Thus *TGTT1* should be an attractive candidate for testing whether similar mechanisms of HTTs exist in plants.

### Varied transcription activities of these non-canonical TEs in pear and apple

Although the LTR-RTs are less likely to be actively expressed in plant tissues during normal development, several exceptions have been reported in various organs belonging to different species, such as *Ogre* elements in leaves, roots and flowers of pea [57], *Grande* elements in leaves of *Zea* and *Tripsacum* [58], eight LTR-RT families in leaves, stalks and roots of *Eucalyptus* genus [59], and *EARE-1* elements in roots, staminate flowers, pistillate flowers, leaves and seeds of *Excoecaria agallocha*, which were all detected as transcriptionally active [60]. In our study, three families (12 *AACA* TEs) from pear were initially detected with transcriptional activity using the published RNA-seq data in the SRA database of NCBI, whereas no transcripts of the six families from peach (*PpTGTT1*), mei (*PmTGTT1* and *PmAACA1*) and apple (*MdTGTT1*, *MdTGTT2* and *MdAACA1*) were identified. In particular, all of the *TGTT* TEs were silenced in the four species, indicating that the transcriptional activities of the *TGTT* and *AACA* TEs varied in different species. The qRT-PCR analyses of three randomly selected elements from pear (*PbrAACA1_IT5*, *PbrAACA4_IT11* and *PbrAACA4_IT14*) also proved that these *TGTT* and *AACA* TEs are transcriptionally active at different levels in different organs. In addition, various elevated transcript levels of the three pear elements were observed following heat, cold and salt treatments, indicating that the *TGTT* and *AACA* TEs could be activated by stresses. This is coincident with the discovery that several other LTR-RTs are frequently activated under stress conditions [28, 60, 61], and also conforms to McClintock’s theory of genome shock in which the enhanced activities of TEs under stress might represent an evolutionary strategy for plant species to increase the chances of survival under unfavorable conditions [62]. Although the transcriptional activities of *TGTT* and *AACA* TEs were not detected in peach and mei using the SRA and EST databases from NCBI, the *PpTGTT1* family has proliferated into four copies since the HT of *PpTGTT1_IT3* from apple, indicating that the element was active for a short time after invasion and then the life cycle of *PpTGTT1* may have been firmly controlled at the post-transcriptional level. Further studies will be conducted in the future.

## Conclusions

*TGTT* and *AACA* are two new types of LTR-RT subfamilies isolated from pear, apple, peach and mei that terminate with atypical dinucleotide structures. Their family and element copy numbers, proliferation time frames and transcriptional activities varied among the four Rosaceae species. HT might have played a significant role in the life cycle of *TGTT1*. These newly identified TEs should be valuable materials for the further investigation of atypical LTR-RTs in other sequenced plant species and will provide interesting insights into their structural evolution and TE-driven genomic evolution.

## Methods

### Genome sequence resources, annotation and classification of *TGTT* and *AACA* LTR-RTs

The genome sequence data for the four Rosaceae species are available in Additional file 1: Table S1. The annotation method of *TGTT* and *AACA* LTR-RT elements has been widely used in previous studies [3, 13, 38]. First, based on the structural analysis, several intact elements were identified by the LTR_STRUC program [39]. Then, all of the identified LTR sequences of the intact elements with clearly defined boundaries were used as queries to detect additional intact elements through sequence homology searches using CROSS_MATCH and CLUSTALW programs with default parameters. Finally, the structures and boundaries of all of the identified LTR-RTs were manually inspected and confirmed, and the TSD sites were defined with one mismatch allowed. Fragments and truncated elements were not analyzed in this study. The *TGTT* and *AACA* LTR-RTs were classified into superfamilies based on the conserved functional domains detected using the BLASTX tool. The queried domains included GAG (for UBN2 superfamily domain, pfam14223), PR (for GAG-pre-integrase domain, pfam13976), INT (for integrase core domain, pfam00665), RT (for reverse transcriptase domain, pfam07727) and RNase H (for Ribonuclease H domain, cd09272). Each individual family was classified using sequence homology comparisons according to the criteria described previously [40].

### Estimation of insertion time

The insertion time of intact elements with TSD sites was estimated by comparing the divergence of their 5′ and 3′ LTR sequences because both LTR sequences of a newly proliferated LTR-RT were believed to be identical at the time of integration [41]. To investigate the nucleotide substitution rate for each element, the two LTR sequences were aligned using the MUSCLE program with default parameters [63]. The insertion time (T) for each intact element was calculated using the formula: T = K/2r, in which the average number of substitutions per aligned site (K) was corrected using the Jukes–Cantor method [64], and 1.3 × 10^−8^ substitutions per site per year was used as the average LTR substitution rate (r) [44].

### Phylogenetic analysis

For each *TGTT* and *AACA* TE, the 5′ LTR sequence, and RT and INT domains were extracted from the intact sequence using a perl script. Sequence alignments were performed by the MAFFT version 7 program with default options [65]. The MEGA 5.2 program implemented with Jukes–Cantor model was employed for building the Maximum Likelihood trees based on 1000 bootstrap replicates [66]. The taxonomic tree was built using the common tree tool on the NCBI website (http://www.ncbi.nlm.nih.gov/Taxonomy/CommonTree/wwwcmt.cgi, last accessed December 25, 2016).

### Identification of orthologous single genes and estimation of genomic sequence divergence

The strategy to identify orthologous single genes between the apple and peach genomes has been used in previous studies [4, 38]. First, the genomic protein and CDS sequences of apple and peach were downloaded from the Phytozome website (http://www.phytozome.net) and set as a database. Then, the BLASTP and orthoMCL software [67] were employed to identify all the orthologous single genes in the two genomes using the same parameters in the previous study [38]. All of the identified single-copy orthologous genes were manually inspected, and gene sequences that contained frame-shift mutations or stop codons were excluded from further analysis.

The Ka, Ks, and Ka/Ks ratio of orthologous single genes and the intra-family INT domains of *AACA* and *TGTT* TEs were calculated using the YN00 program implemented in the PAML software package [68].

The CDS sequence identities of orthologous single genes in apple and peach were computed using an in-house perl script, which ran in the following three steps: (1) All of the identified orthologous single-gene pairs were separately aligned using MUSCLE software; (2) For each orthologous single-gene pair, the numbers of identical nucleotides (I), mismatches (M) and gaps (G) were counted. (3) Gene identities without gaps were calculated using the formula: I/(I + M) × 100, and gene identities with gaps were calculated using the formula: I/(I + M + G) × 100 (Additional file 1: Table S7). The sequence identity analysis between the *TGTT1* TEs were also conducted using the same strategy.

### PCR and sequencing analysis

The total genomic DNA of the four Rosaceae species were extracted from the young leaves using the improved cetyltrimethyl ammonium bromide method. In total, 11 *TGTT* and 5 *AACA* TEs from eight families were randomly selected for validation. For each element, 300-bp 5′-flanking sequences and 300-bp 3′-flanking sequences of both LTR sequences were extracted and used to design primers (Additional file 2: Figure S2 and Additional file 1: Table S5). Polymerase chain reactions (PCR) were performed in a total volume of 25 ml, containing 1 ml of 50 ng/ml genomic DNA template, 2.5 ml of 10× buffer (without MgCl2), 2.5 ml of 2.5 mM dNTP mixture, 2.5 ml of 25 mM MgCl_2_, 0.8 ml each of forward and reverse primer (10 pmol/ml) and 0.2 ml of 5 U/ml Taq polymerase (Takara Biotechnology Company, Dalian, China). The reactions were performed with the following conditions: 94 °C for 3 min, then 35 cycles of 94 °C for 30 s, 57 °C for 40 s and 72 °C for 2 min, and a final step at 72 °C for 10 min. The PCR products were resolved on 1% agarose and detected by ethidium bromide staining. The analyses were performed three times and loaded on independent gels. All of the specific PCR products were isolated with the DNA Gel Extraction kit AxyPrep (Axygen Inc.). The fragments were cloned into the pMD19-T vector (Takara, China), and the plasmids were sequenced by Invitrogen (Shanghai, China).

### Transcriptional activity analysis of *TGTT* and *AACA* elements

The Illumina RNA-Seq data of four samples from the SRA database of NCBI (https://www.ncbi.nlm.nih.gov/), including pear fruit peel (*P. bretshneideri* ‘Dangshansuli’, SRX298075), pear bud (*P. bretshneideri* ‘Dangshansuli’, SRX147917) and apple leaves (*M. domestica* ‘Gala’, SRX1150925), were used to identify the transcriptional patterns. For each element, the whole-nucleotide sequences were used as queries to construct HISAT alignments using the default parameters [69].

### Stress treatments and LTR-RT expression analysis by quantitative RT-PCR

The scions of ‘Dangshansuli’ (*P. bretschneideri*) were grafted to 1-year-old *Pyrus betulifolia* plants and grown in a culture room at 25 °C under long-day conditions (16 h light/8 h dark) for 30 d prior to stresses. For the cold treatment, seedlings were placed in a growth incubator set at 4 °C for 0, 1, 3, 6 and 12 h. For heat stress treatments, plants were transferred to 40 °C for 0, 1, 3, 6 and 12 h. Salt stress was carried out by watering the plants with 1600 mM NaCl solution for 0, 1, 3, 6 and 12 h. All of the samples were recovered for 24 h.

The total RNA from fruit flesh at four developmental periods (32, 65, 99 and 143 d after flower bloom), leaves, pericarp, pollen and stylet were extracted using a cetyltrimethyl ammonium bromide-based method and digested with RNase-free DNase I (Thermo) to remove DNA contamination. According to the manufacturer’s instructions, 1 mg of total RNA was reverse transcribed into cDNA using the ReverTra Ace qPCR RT Kit (Toyobo, Shanghai, China). Specific primers for *PbrAACA1_IT5*, *PbrAACA4_IT11* and *PbrAACA4_IT14* were designed using the Primer 5 software (Additional file 1: Table S7). Quantitative real-time RT-PCR (qRT-PCR) was used for measuring the transcript levels of the three LTR-RTs. The PCR solution (20 ml) contained 10 ml of SYBR-Green PCR Master Mix (SYBR Premix EX TaqTM, TaKaRa), 0.25 mM each of forward and reverse primer, 100 ng of cDNA template, and nuclease free water. The qRT-PCR analysis with a SYBR Green PCR kit was performed in a Light Cycler 480 (Roche, USA) Real-Time System. The reactions were conducted under the following conditions: 95 °C for 5 min, then 45 cycles of 94°C4for 10 s, 60° C0for 30 s and 72 °C for 30 s, followed by a final extension at 72 °C for 3 min. The 2^−ΔΔCT^ method [70] was used to calculate the relative expression levels of each gene. Each sample was analyzed for three replicates. The mRNA capping enzyme gene (*Pbr035952.1*) and cytochrome B561 gene (*Pbr013721.1*) were used as internal controls for five different tissue and three stress treatments, respectively, and to normalize the relative expression levels of each LTR-RT. The expression analysis of each time point was repeated three times.

## Additional files

Additional file 1: Table S1. List of four species and their genomic sequence and DNA material source information used in this study. **Table S2.** Sequence identity between all TGTT and AACA consensus sequences in the four Rosaceae genomes. **Table S3.** Summary of *TGTT* and *AACA* elements identified in *P. bretschneideri*, *M. domestcia*, *P. persica* and *P. mume*. **Table S4.** Primers used for wet lab validations. **Table S5.** Sequence identity between all TGTT1 elements identified in the four Rosaceae genomes. **Table S6.** Sequence divergence of orthologous singletons between apple and peach. **Table S7.** Sequence identity of the orthologous singletons between apple and peach. **Table S8.** Primers used for Real time Quantitative PCR. (XLSX 142 kb)

Additional file 2: Figure S1. Sequence alignment of the *TGTT1* elements. **Figure S2.** Wet laboratory validation of the *TGTT* and *AACA* elements. **Figure S3.** Multiple alignments of PBS sites from *TGTT*, *AACA* and normal TGCA elements of the *Ale* lineage. **Figure S4.** INT phylogenetic relationships among *TGTT*, *AACA* and normal TGCA elements. **Figure S5**. Evidence of transcriptional activity in five *TGTT* and *AACA* elements. (PDF 2824 kb)

## Abbreviations

HTs: Horizontal transfers
HTT: Horizontal TE transfer
HTTEs: Horizontal transfer of transposable elements
int/INT: Integrase
LARDs: Large retrotransposon derivatives
LTR-RTs: Long terminal repeat retrotransposons
Mya: Million years ago
Mys: Million years
PBS: Primer-binding site
PPT: Polypurine tract
PR: Protease
qRT-PCR: Quantitative real time polymerase chain reaction
rh/RH: RNase H
rt/RT: Reverse transcriptase
SRA: Sequence read archive
TEs: Transposable elements
TRIMs: Terminal-repeat retrotransposons in miniature
TSD: Target site duplication
