# Supplementary material for: TGTT and AACA: two transcriptionally active LTR retrotransposon subfamilies with a specific LTR structure and horizontal transfer in four Rosaceae species
Source: Mob DNA. 2017 Oct 27;8:14. doi: 10.1186/s13100-017-0098-8 (PMC5659011; doi:10.1186/s13100-017-0098-8)
Supplement: Supplementary file 2 — Sequence alignment of the TGTT1 elements. Figure S2. Wet laboratory validation of the TGTT and AACA elements. Figure S3. Multiple alignments of PBS sites from TGTT, AACA and normal TGCA elements of the Ale lineage. Figure S4. INT phylogenetic relationships among TGTT, AACA and normal TGCA elements. Figure S5. Evidence of transcriptional activity in five TGTT and AACA elements. (PDF 2824 kb) [file 13100_2017_98_MOESM2_ESM.pdf]

Supplementary Figure Legends

Figure S1. Sequence alignment of the *TGTT1* elements

Sequence identities over 70% were shaded in blue. The TSD site, 5′LTR, PBS, PPT and 3′LTR regions are indicated using red lines and arrows.

Figure S2. Wet laboratory validation of the TGTT and AACA elements.

Each element was randomly selected and analyzed from each family. A schematic view of the LTR-RT, with the location of the region that was amplified (dashed pink boxes), a picture of the PCR amplification of the LTRs using DNA material of the four Rosaceae species (see Table S1), and the alignments of the sequenced PCR products together with the sequences that were mined from the genome sequence are provided. The positions of TSDs, beginnings and ends of LTRs, PBS and PPT are also indicated.

Figure S3. Multiple alignments of PBS sites from TGTT, AACA and normal TGCA elements of the *Ale* lineage.

A 20-bp region of each PBS site was extracted from all of the *TGTT* and *AACA* elements, as were the consensus sequences of the other four *Copia Ale* families from pear [38]. Sequence identities over 70% are shaded in blue.

Figure S4. INT phylogenetic relationships among TGTT, AACA and normal TGCA elements.

The INT domains (the integrase core domain, pfam00665) of 67 elements from the six *Copia-like TGTT* and *AACA* families and the consensus sequences of each normal *Copia* TGCA family from pear [38] were used to build the phylogenetic tree. The *TGTT* and *AACA* elements are indicated by various colored branches, and the normal TGCA families are shown in gray. The nucleotide sequence distances are indicated by the scales.

Figure S5. Evidence of transcriptional activity in five *TGTT* and *AACA* elements.

For each element, the whole-nucleotide sequences were used as queries to construct the HISAT alignments using the RNA-seq data from the NCBI species Sequence Read Archive database. Normal short pair reads are shown in the gray bar. Too long paired reads are shown in the purplish red bar. One-sided sequences are indicated by green or blue boxes.

Figure S1.

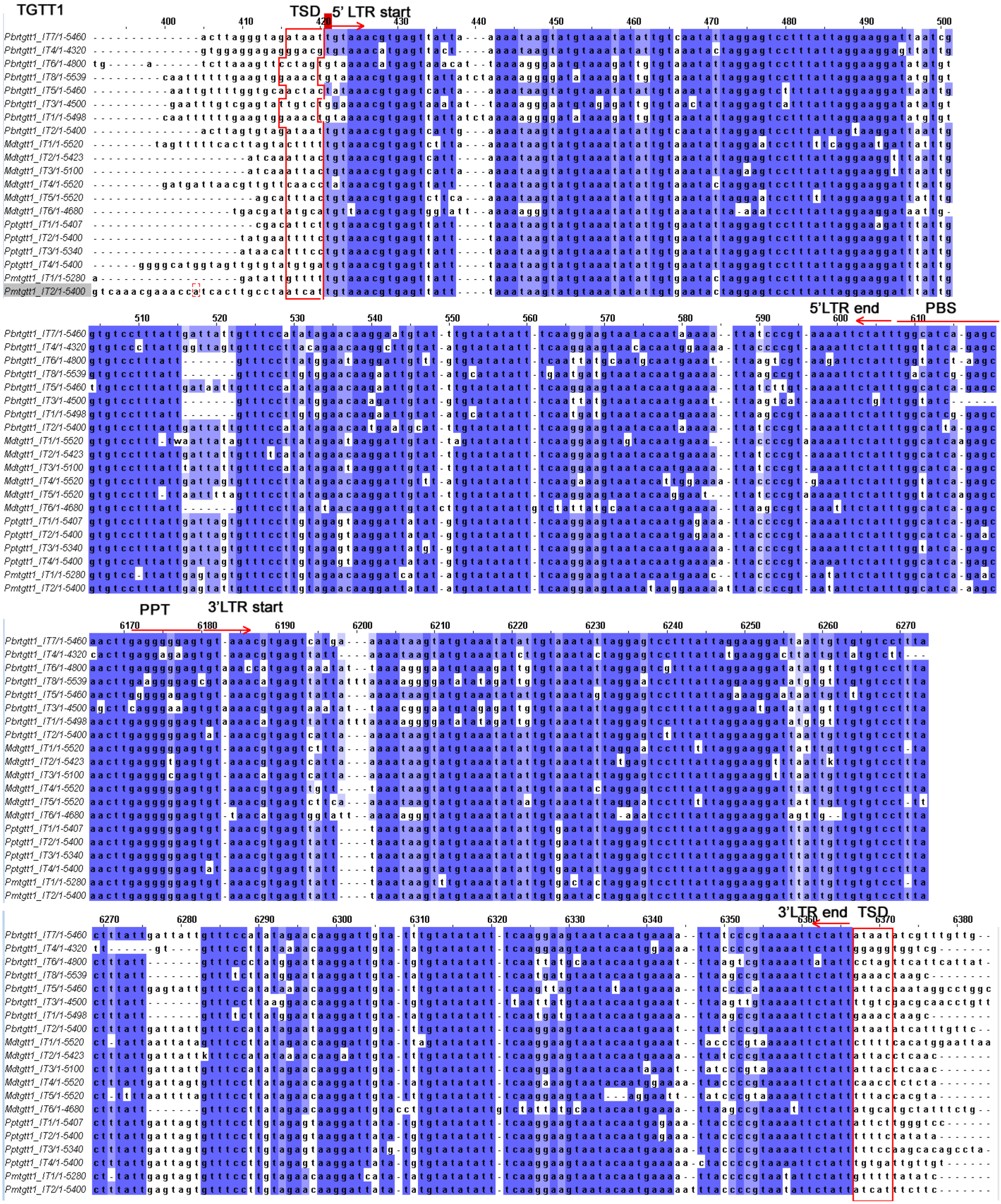

TGTT1
TSD
5' LTR start
5'LTR end
PBS
PPT
3'LTR start
3'LTR end
TSD
Pbrtgtt1_IT7/1-5460
Pbrtgtt1_IT4/1-4320
Pbrtgtt1_IT6/1-4800
Pbrtgtt1_IT8/1-5539
Pbrtgtt1_IT5/1-5460
Pbrtgtt1_IT3/1-4500
Pbrtgtt1_IT1/1-5498
Pbrtgtt1_IT2/1-5400
Mdtgtt1_IT1/1-5520
Mdtgtt1_IT2/1-5423
Mdtgtt1_IT3/1-5100
Mdtgtt1_IT4/1-5520
Mdtgtt1_IT5/1-5520
Mdtgtt1_IT6/1-4680
Pptgtt1_IT1/1-5407
Pptgtt1_IT2/1-5400
Pptgtt1_IT3/1-5340
Pptgtt1_IT4/1-5400
Pmtgtt1_IT1/1-5280
Pmtgtt1_IT2/1-5400

Figure S1.

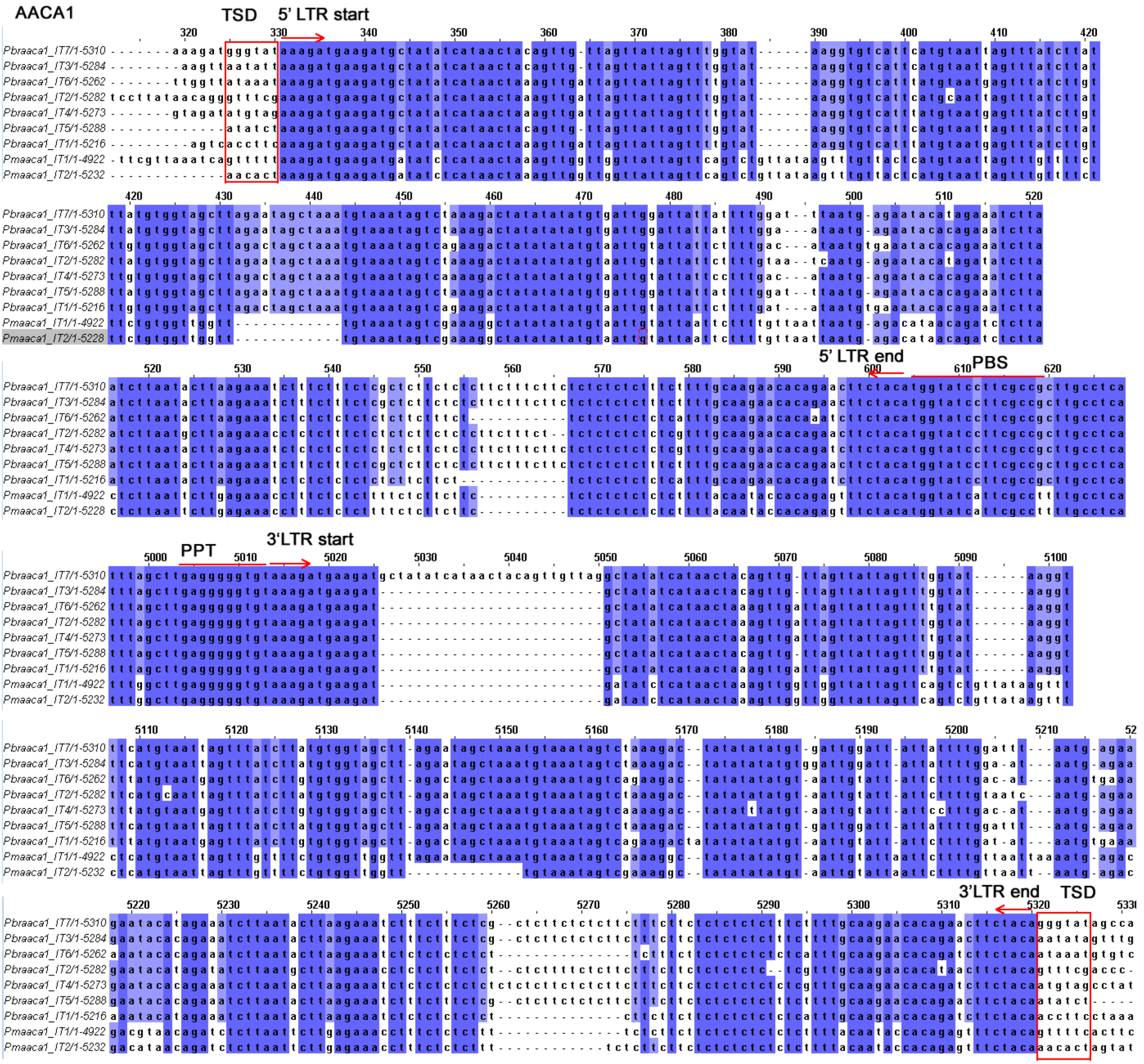

AACA1
TSD
5' LTR start
5' LTR end
PBS
PPT
3'LTR start
3'LTR end
TSD
Pbraaca1_IT7/1-5310
Pbraaca1_IT3/1-5284
Pbraaca1_IT6/1-5262
Pbraaca1_IT2/1-5282
Pbraaca1_IT4/1-5273
Pbraaca1_IT5/1-5288
Pbraaca1_IT1/1-5216
Pmaaca1_IT1/1-4922
Pmaaca1_IT2/1-5232

Figure S2.

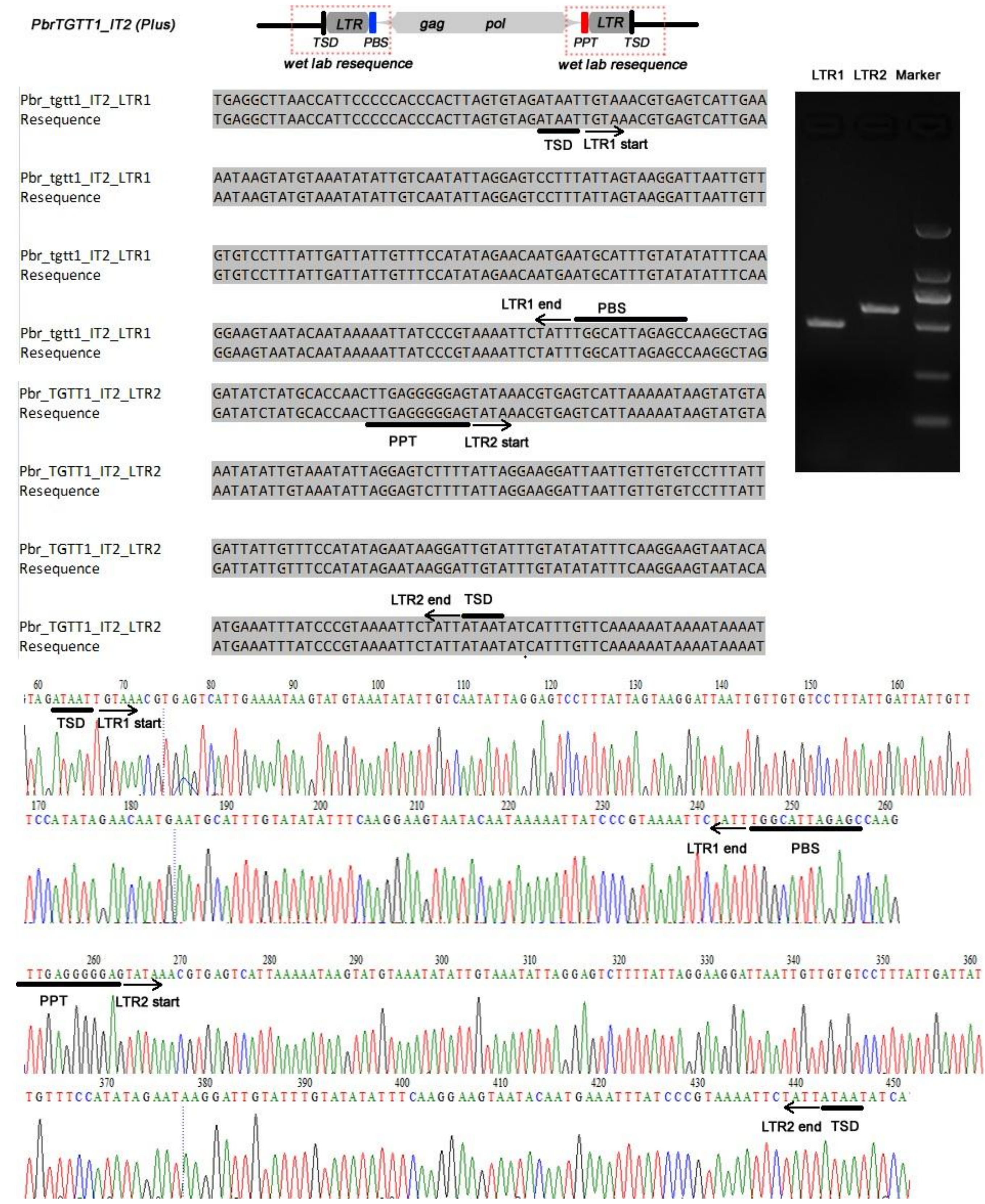

PbrTGTT1_IT2 (Plus)
LTR
gag
pol
LTR
TSD
PBS
PPT
TSD
wet lab resequence
wet lab resequence
LTR1 LTR2 Marker
Pbr_tgtt1_IT2_LTR1
Resequence
TGAGGCTTAACCATTCCCCCACCCACTTAGTGTAGATAATTGTAAACGTGAGTCATTGAA
TSD
LTR1 start
AATAAGTATGTAAATATATTGTCAATATTAGGAGTCCTTTATTAGTAAGGATTAATTGTT
GTGTCCTTTATTGATTATTGTTTCCATATAGAACAATGAATGCATTTGTATATATTTCAA
LTR1 end
PBS
GGAAGTAATACAATAAAAATTATCCCGTAAAATTCTATTTGGCATTAGAGCCAAGGCTAG
Pbr_TGTT1_IT2_LTR2
Resequence
GATATCTATGCACCAACTTGAGGGGGAGTATAAACGTGAGTCATTAAAAATAAGTATGTA
PPT
LTR2 start
AATATATTGTAAATATTAGGAGTCTTTTATTAGGAAGGATTAATTGTTGTGTCCTTTATT
GATTATTGTTTCCATATAGAATAAGGATTGTATTTGTATATATTTCAAGGAAGTAATACA
LTR2 end
TSD
ATGAAATTTATCCCGTAAAATTCTATTATAATATCATTTGTTCAAAAAATAAAATAAAAT
TSD
LTR1 start
LTR1 end
PBS
PPT
LTR2 start
LTR2 end
TSD

Figure S2.

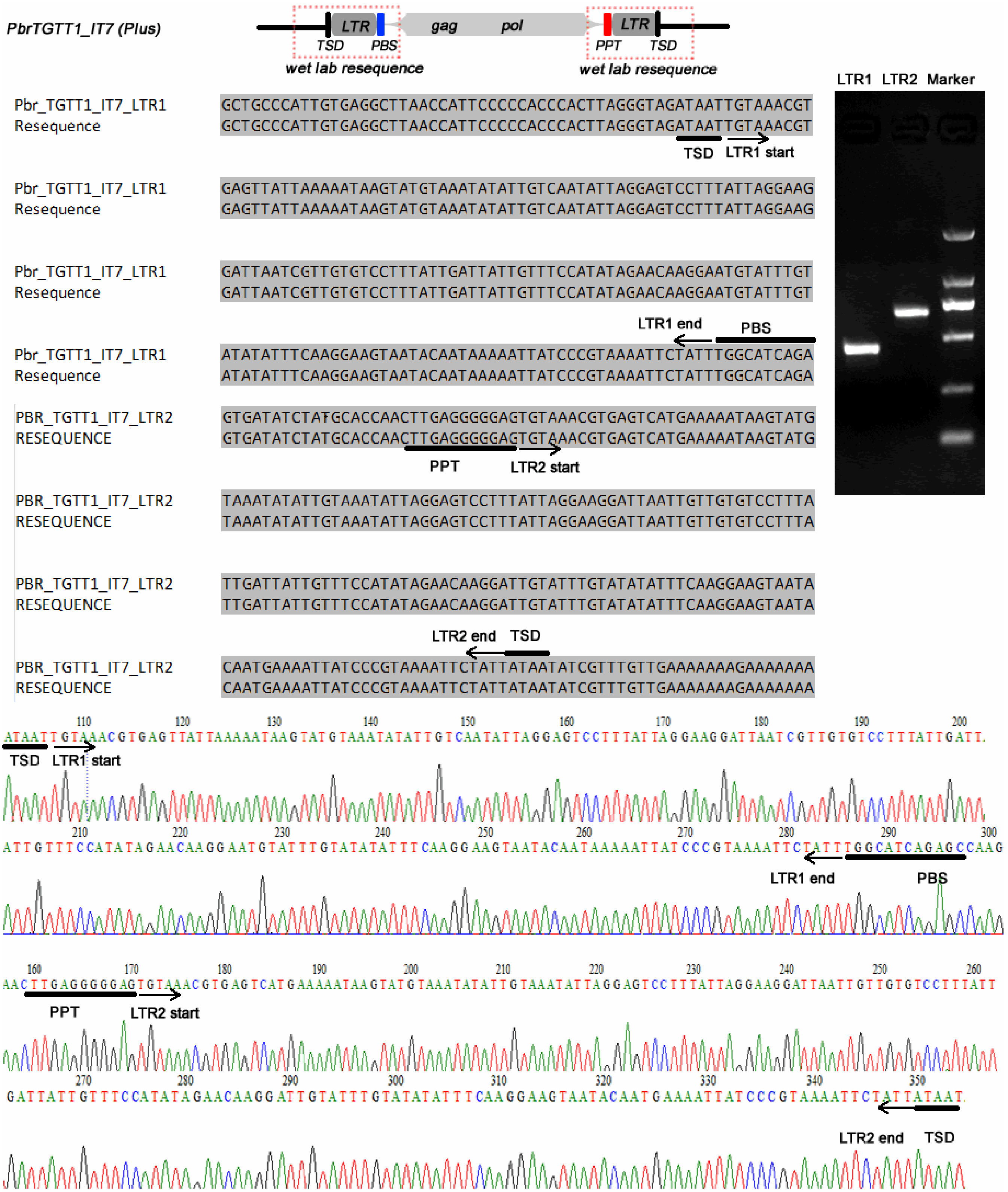
PbrTGTT1_IT7 (Plus)
LTR
TSD
PBS
gag
pol
PPT
wet lab resequence
wet lab resequence
LTR1 LTR2 Marker
Pbr_TGTT1_IT7_LTR1 GCTGCCCATTGTGAGGCTTAACCATTCCCCCACCCACTTAGGGTAGATAATTGTAAACGT
Resequence GCTGCCCATTGTGAGGCTTAACCATTCCCCCACCCACTTAGGGTAGATAATTGTAAACGT
TSD LTR1 start
Pbr_TGTT1_IT7_LTR1 GAGTTATTAAAAATAAGTATGTAAATATATTGTCAATATTAGGAGTCCTTTATTAGGAAG
Resequence GAGTTATTAAAAATAAGTATGTAAATATATTGTCAATATTAGGAGTCCTTTATTAGGAAG
Pbr_TGTT1_IT7_LTR1 GATTAATCGTTGTGTCCTTTATTGATTATTGTTTCCATATAGAACAAGGAATGTATTTGT
Resequence GATTAATCGTTGTGTCCTTTATTGATTATTGTTTCCATATAGAACAAGGAATGTATTTGT
LTR1 end PBS
Pbr_TGTT1_IT7_LTR1 ATATATTTCAAGGAAGTAATACAATAAAAATTATCCCGTAAAATTCTATTTGGCATCAGA
Resequence ATATATTTCAAGGAAGTAATACAATAAAAATTATCCCGTAAAATTCTATTTGGCATCAGA
PBR_TGTT1_IT7_LTR2 GTGATATCTATGCACCAACTTGAGGGGGAGTGTAAACGTGAGTCATGAAAAATAAGTATG
RESEQUENCE GTGATATCTATGCACCAACTTGAGGGGGAGTGTAAACGTGAGTCATGAAAAATAAGTATG
PPT LTR2 start
PBR_TGTT1_IT7_LTR2 TAAATATATTGTAAATATTAGGAGTCCTTTATTAGGAAGGATTAATTGTTGTGTCCTTTA
RESEQUENCE TAAATATATTGTAAATATTAGGAGTCCTTTATTAGGAAGGATTAATTGTTGTGTCCTTTA
PBR_TGTT1_IT7_LTR2 TTGATTATTGTTTCCATATAGAACAAGGATTGTATTTGTATATATTTCAAGGAAGTAATA
RESEQUENCE TTGATTATTGTTTCCATATAGAACAAGGATTGTATTTGTATATATTTCAAGGAAGTAATA
LTR2 end TSD
PBR_TGTT1_IT7_LTR2 CAATGAAAATTATCCCGTAAAATTCTATTATAATATCGTTTGTTGAAAAAAAGAAAAAAA
RESEQUENCE CAATGAAAATTATCCCGTAAAATTCTATTATAATATCGTTTGTTGAAAAAAAGAAAAAAA
TSD LTR1 start
LTR1 end PBS
PPT LTR2 start
LTR2 end TSD

Figure S2.

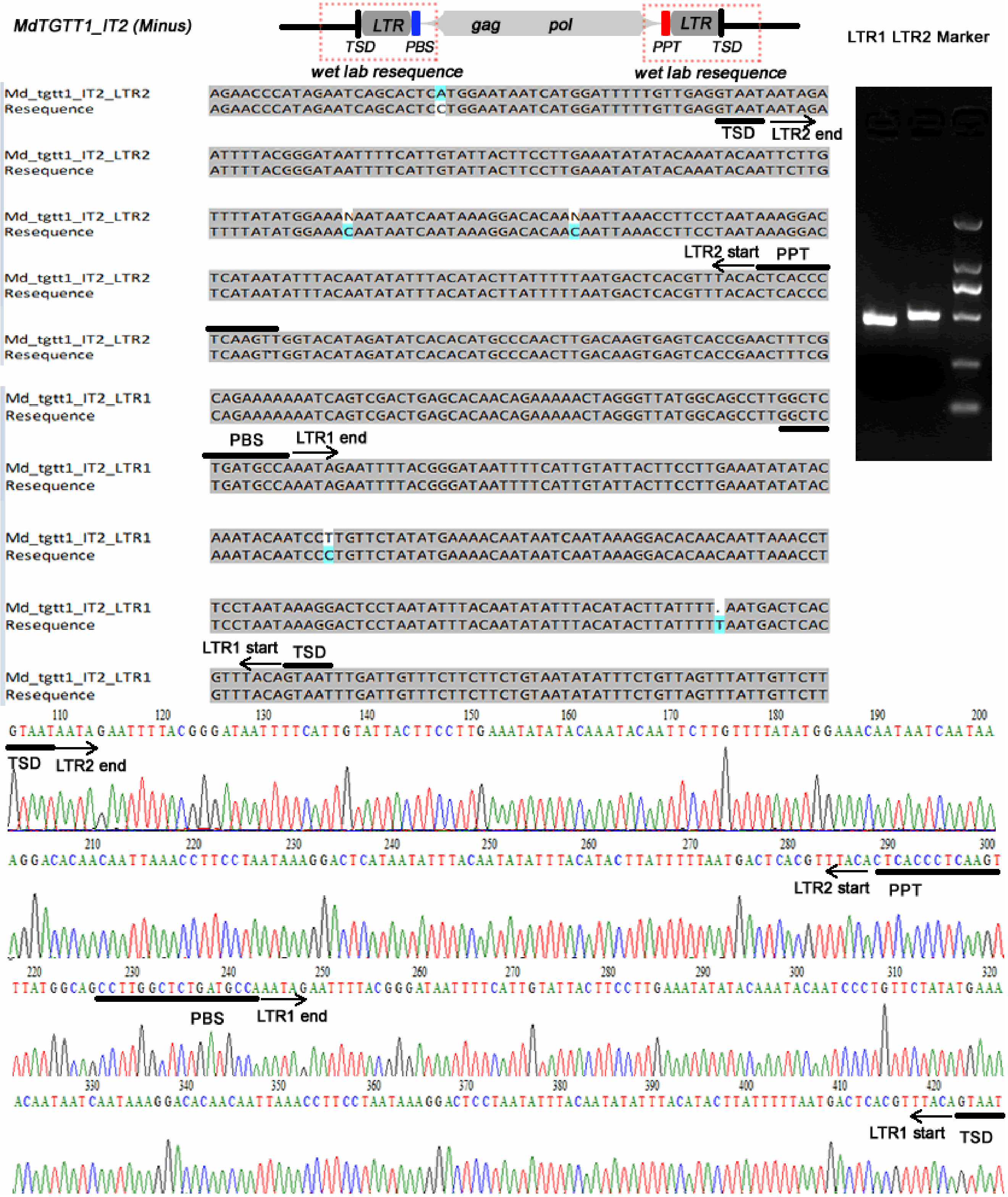
MdTGTT1_IT2 (Minus)
LTR
PBS
gag
pol
PPT
LTR
TSD
TSD
wet lab resequence
wet lab resequence
LTR1 LTR2 Marker
Md_tgtt1_IT2_LTR2
AGAACCCATAGAATCAGCACTCATGGAATAATCATGGATTTTTGTTGAGGTAATAATAGA
Resequence
AGAACCCATAGAATCAGCACTCCTGGAATAATCATGGATTTTTGTTGAGGTAATAATAGA
TSD
LTR2 end
Md_tgtt1_IT2_LTR2
ATTTTACGGGATAATTTTCATTGTATTACTTCCTTGAAATATATACAAATACAATTCTTG
Resequence
ATTTTACGGGATAATTTTCATTGTATTACTTCCTTGAAATATATACAAATACAATTCTTG
Md_tgtt1_IT2_LTR2
TTTTATATGGAAANAATAATCAATAAAGGACACAANAATTAAACCTTCCTAATAAAGGAC
Resequence
TTTTATATGGAAACAATAATCAATAAAGGACACAACAATTAAACCTTCCTAATAAAGGAC
LTR2 start
PPT
Md_tgtt1_IT2_LTR2
TCATAATATTTACAATATATTTACATACTTATTTTTAATGACTCACGTTTACACTCACCC
Resequence
TCATAATATTTACAATATATTTACATACTTATTTTTAATGACTCACGTTTACACTCACCC
Md_tgtt1_IT2_LTR2
TCAAGTTGGTACATAGATATCACACATGCCCAACTTGACAAGTGAGTCACCGAACTTTCG
Resequence
TCAAGTTGGTACATAGATATCACACATGCCCAACTTGACAAGTGAGTCACCGAACTTTCG
Md_tgtt1_IT2_LTR1
CAGAAAAAAATCAGTCGACTGAGCACAACAGAAAAACTAGGGTTATGGCAGCCTTGGCTC
Resequence
CAGAAAAAAATCAGTCGACTGAGCACAACAGAAAAACTAGGGTTATGGCAGCCTTGGCTC
PBS
LTR1 end
Md_tgtt1_IT2_LTR1
TGATGCCAAATAGAATTTTACGGGATAATTTTCATTGTATTACTTCCTTGAAATATATAC
Resequence
TGATGCCAAATAGAATTTTACGGGATAATTTTCATTGTATTACTTCCTTGAAATATATAC
Md_tgtt1_IT2_LTR1
AAATACAATCCTTGTTCTATATGAAAACAATAATCAATAAAGGACACAACAATTAAACCT
Resequence
AAATACAATCCCTGTTCTATATGAAAACAATAATCAATAAAGGACACAACAATTAAACCT
Md_tgtt1_IT2_LTR1
TCCTAATAAAGGACTCCTAATATTTACAATATATTTACATACTTATTTT. AATGACTCAC
Resequence
TCCTAATAAAGGACTCCTAATATTTACAATATATTTACATACTTATTTTTAATGACTCAC
LTR1 start
TSD
Md_tgtt1_IT2_LTR1
GTTTACAGTAATTTGATTGTTTCTTCTTCTGTAATATATTTCTGTTAGTTTATTGTTCTT
Resequence
GTTTACAGTAATTTGATTGTTTCTTCTTCTGTAATATATTTCTGTTAGTTTATTGTTCTT
TSD
LTR2 end
LTR2 start
PPT
PBS
LTR1 end
LTR1 start
TSD

Figure S2.

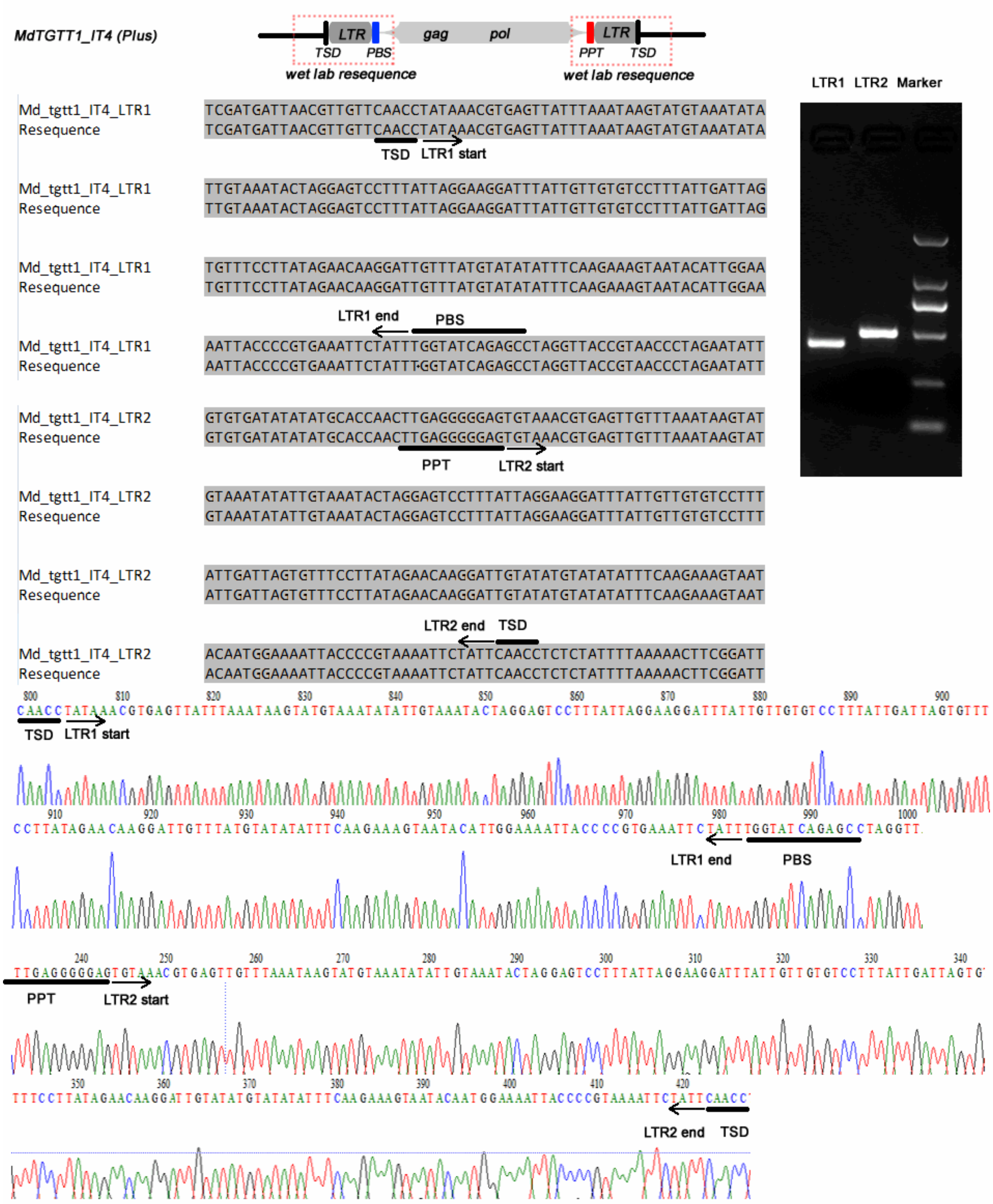

MdTGTT1_IT4 (Plus)
LTR
gag
pol
LTR
TSD
PBS
PPT
TSD
wet lab resequence
wet lab resequence
LTR1 LTR2 Marker
Md_tgtt1_IT4_LTR1
Resequence
TSD
LTR1 start
LTR1 end
PBS
Md_tgtt1_IT4_LTR2
PPT
LTR2 start
LTR2 end
TSD

Figure S2.

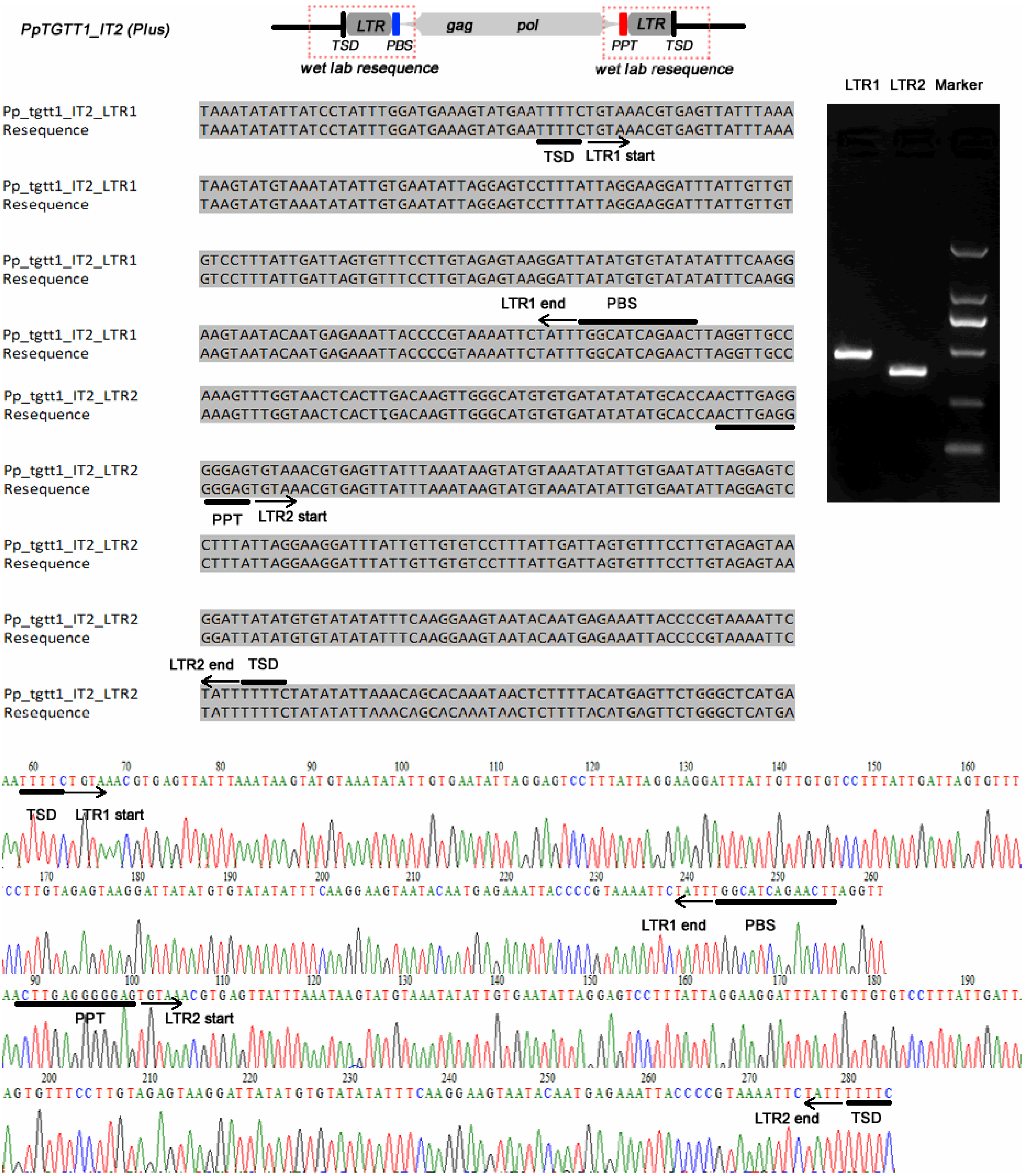
PpTGTT1_IT2 (Plus)
LTR
TSD
PBS
gag
pol
LTR
PPT
TSD
wet lab resequence
wet lab resequence
LTR1 LTR2 Marker
Pp_tgtt1_IT2_LTR1
Resequence
TAAATATATTATCCTATTTGGATGAAAGTATGAATTTTCTGTAAACGTGAGTTATTTAAA
TAAATATATTATCCTATTTGGATGAAAGTATGAATTTTCTGTAAACGTGAGTTATTTAAA
TSD
LTR1 start
Pp_tgtt1_IT2_LTR1
Resequence
TAAGTATGTAAATATATTGTGAATATTAGGAGTCCTTTATTAGGAAGGATTTATTGTTGT
TAAGTATGTAAATATATTGTGAATATTAGGAGTCCTTTATTAGGAAGGATTTATTGTTGT
Pp_tgtt1_IT2_LTR1
Resequence
GTCCTTTATTGATTAGTGTTTCCTTGTAGAGTAAGGATTATATGTGTATATATTTCAAGG
GTCCTTTATTGATTAGTGTTTCCTTGTAGAGTAAGGATTATATGTGTATATATTTCAAGG
LTR1 end
PBS
Pp_tgtt1_IT2_LTR1
Resequence
AAGTAATACAATGAGAAATTACCCCGTAAAATTCTATTTGGCATCAGAACTTAGGTTGCC
AAGTAATACAATGAGAAATTACCCCGTAAAATTCTATTTGGCATCAGAACTTAGGTTGCC
Pp_tgtt1_IT2_LTR2
Resequence
AAAGTTTGGTAACTCACTTGACAAGTTGGGCATGTGTGATATATATGCACCAACTTGAGG
AAAGTTTGGTAACTCACTTGACAAGTTGGGCATGTGTGATATATATGCACCAACTTGAGG
Pp_tgtt1_IT2_LTR2
Resequence
GGGAGTGTAAACGTGAGTTATTTAAATAAGTATGTAAATATATTGTGAATATTAGGAGTC
GGGAGTGTAAACGTGAGTTATTTAAATAAGTATGTAAATATATTGTGAATATTAGGAGTC
PPT
LTR2 start
Pp_tgtt1_IT2_LTR2
Resequence
CTTTATTAGGAAGGATTTATTGTTGTGTCCTTTATTGATTAGTGTTTCCTTGTAGAGTAA
CTTTATTAGGAAGGATTTATTGTTGTGTCCTTTATTGATTAGTGTTTCCTTGTAGAGTAA
Pp_tgtt1_IT2_LTR2
Resequence
GGATTATATGTGTATATATTTCAAGGAAGTAATACAATGAGAAATTACCCCGTAAAATTC
GGATTATATGTGTATATATTTCAAGGAAGTAATACAATGAGAAATTACCCCGTAAAATTC
LTR2 end
TSD
Pp_tgtt1_IT2_LTR2
Resequence
TATTTTTTCTATATATTAAACAGCACAAATAACTCTTTTACATGAGTTCTGGGCTCATGA
TATTTTTTCTATATATTAAACAGCACAAATAACTCTTTTACATGAGTTCTGGGCTCATGA
TSD
LTR1 start
LTR1 end
PBS
PPT
LTR2 start
LTR2 end
TSD

Figure S2.

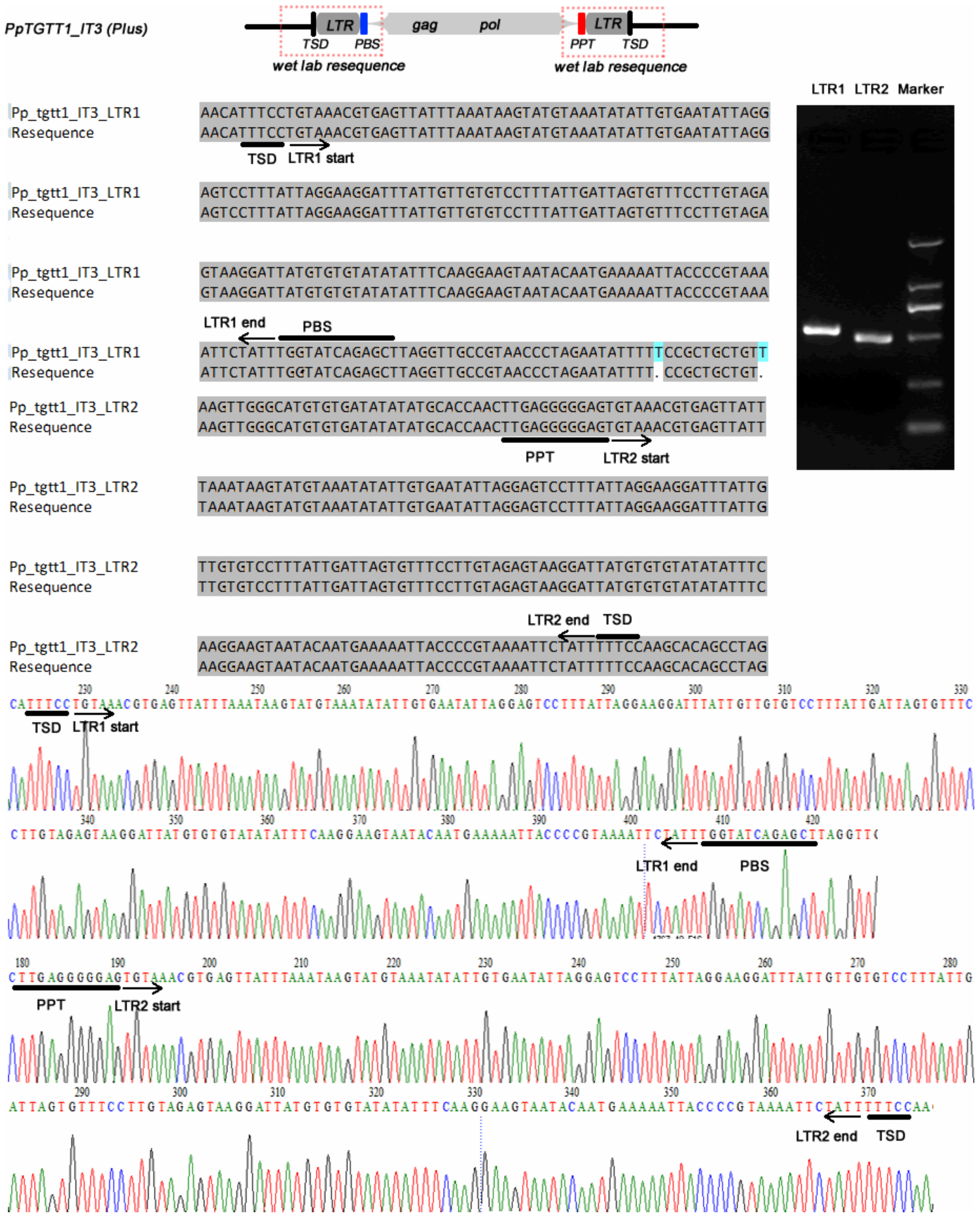
PpTGTT1_IT3 (Plus)
LTR
TSD
PBS
gag
pol
LTR
PPT
TSD
wet lab resequence
wet lab resequence
LTR1 LTR2 Marker
Pp_tgtt1_IT3_LTR1
Resequence
TSD
LTR1 start
LTR1 end
PBS
Pp_tgtt1_IT3_LTR2
Resequence
PPT
LTR2 start
LTR2 end
TSD

Figure S2.

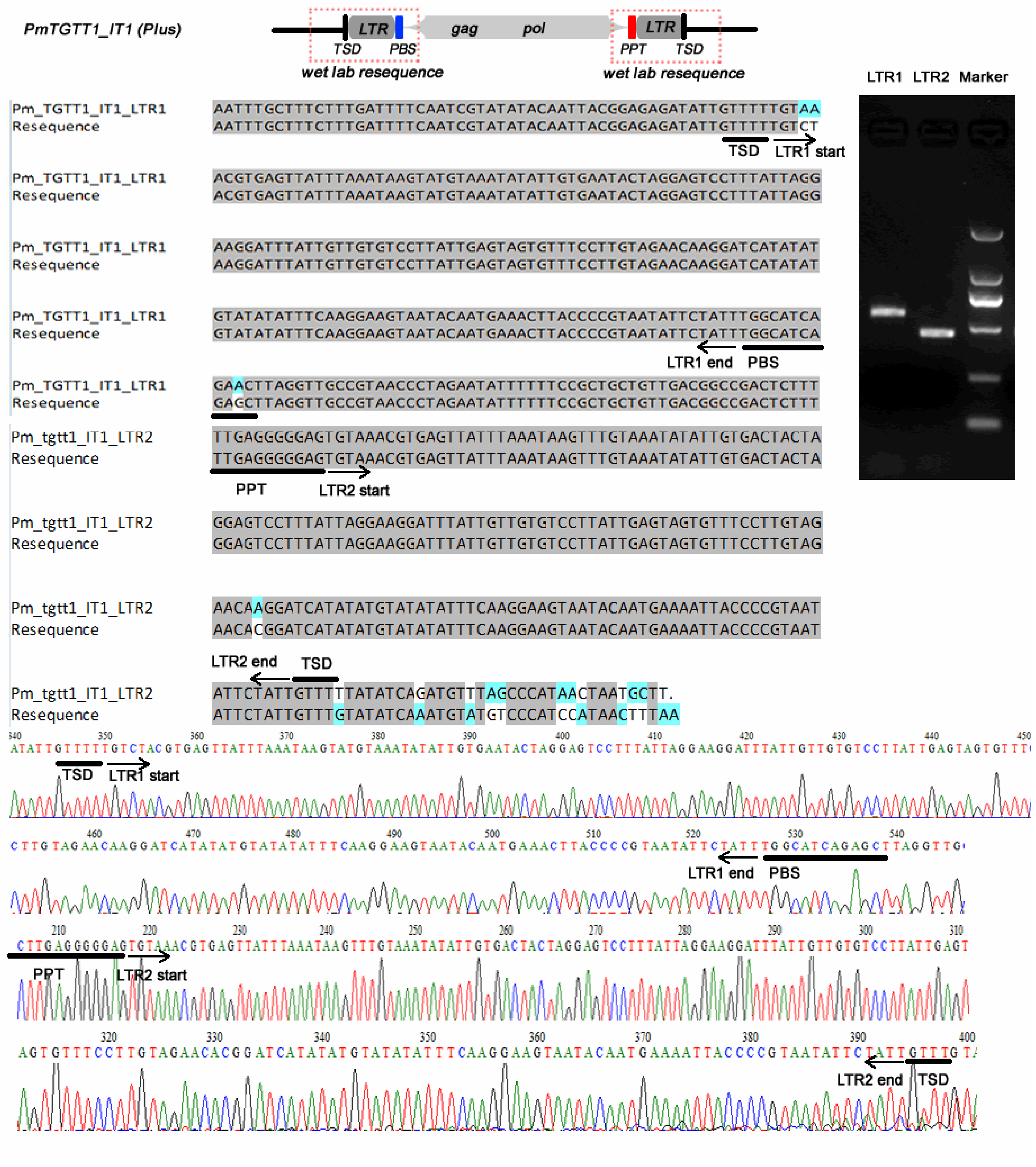
PmTGTT1_IT1 (Plus)
LTR
gag
pol
LTR
TSD
PBS
PPT
TSD
wet lab resequence
wet lab resequence
LTR1 LTR2 Marker
Pm_TGTT1_IT1_LTR1 AATTTGCTTTCTTTGATTTTCAATCGTATATACAATTACGGAGAGATATTGTTTTTGTAA
Resequence AATTTGCTTTCTTTGATTTTCAATCGTATATACAATTACGGAGAGATATTGTTTTTGTCT
TSD
LTR1 start
Pm_TGTT1_IT1_LTR1 ACGTGAGTTATTTAAATAAGTATGTAAATATATTGTGAATACTAGGAGTCCTTTATTAGG
Resequence ACGTGAGTTATTTAAATAAGTATGTAAATATATTGTGAATACTAGGAGTCCTTTATTAGG
Pm_TGTT1_IT1_LTR1 AAGGATTTATTGTTGTGTCCTTATTGAGTAGTGTTTCCTTGTAGAACAAGGATCATATAT
Resequence AAGGATTTATTGTTGTGTCCTTATTGAGTAGTGTTTCCTTGTAGAACAAGGATCATATAT
Pm_TGTT1_IT1_LTR1 GTATATATTTCAAGGAAGTAATACAATGAAACTTACCCCGTAATATTCTATTTGGCATCA
Resequence GTATATATTTCAAGGAAGTAATACAATGAAACTTACCCCGTAATATTCTATTTGGCATCA
LTR1 end
PBS
Pm_TGTT1_IT1_LTR1 GAACTTAGGTTGCCGTAACCCTAGAATATTTTTTCCGCTGCTGTTGACGGCCGACTCTTT
Resequence GAGCTTAGGTTGCCGTAACCCTAGAATATTTTTTCCGCTGCTGTTGACGGCCGACTCTTT
Pm_tgtt1_IT1_LTR2 TTGAGGGGGAGTGTAAACGTGAGTTATTTAAATAAGTTTGTAAATATATTGTGACTACTA
Resequence TTGAGGGGGAGTGTAAACGTGAGTTATTTAAATAAGTTTGTAAATATATTGTGACTACTA
PPT
LTR2 start
Pm_tgtt1_IT1_LTR2 GGAGTCCTTTATTAGGAAGGATTTATTGTTGTGTCCTTATTGAGTAGTGTTTCCTTGTAG
Resequence GGAGTCCTTTATTAGGAAGGATTTATTGTTGTGTCCTTATTGAGTAGTGTTTCCTTGTAG
Pm_tgtt1_IT1_LTR2 AACAAGGATCATATATGTATATATTTCAAGGAAGTAATACAATGAAAATTACCCCGTAAT
Resequence AACACGGATCATATATGTATATATTTCAAGGAAGTAATACAATGAAAATTACCCCGTAAT
LTR2 end
TSD
Pm_tgtt1_IT1_LTR2 ATTCTATTGTTTTTATATCAGATGTTTAGCCCATAACTAATGCTT.
Resequence ATTCTATTGTTTGTATATCAAATGTATGTCCCATCCATAACTTTAA
TSD
LTR1 start
LTR1 end
PBS
PPT
LTR2 start
LTR2 end
TSD

Figure S2.

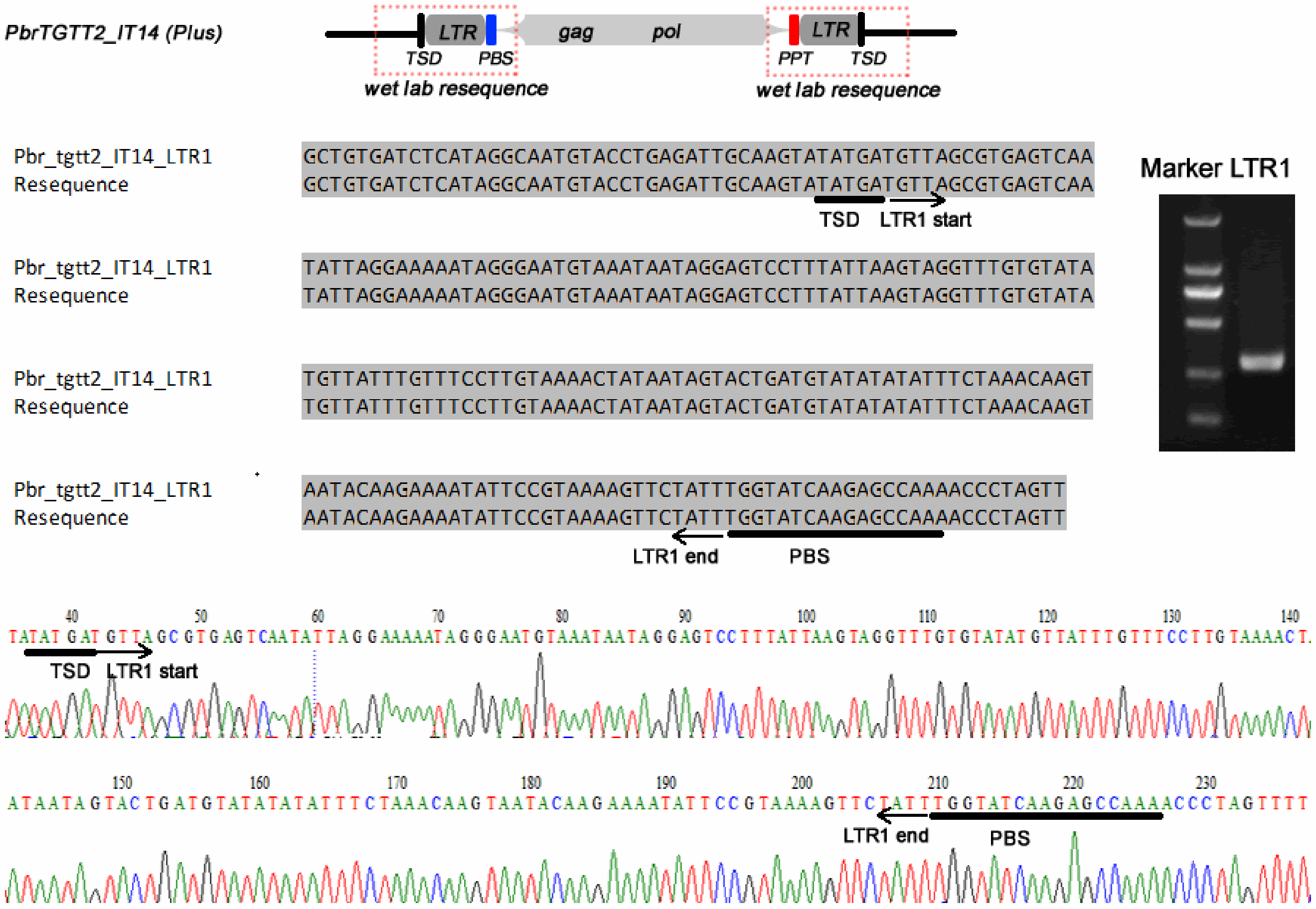
PbrTGTT2_IT14 (Plus)
LTR
TSD
PBS
gag
pol
PPT
LTR
TSD
wet lab resequence
wet lab resequence
Pbr_tgtt2_IT14_LTR1
Resequence
GCTGTGATCTCATAGGCAATGTACCTGAGATTGCAAGTATATGATGTTAGCGTGAGTCAA
GCTGTGATCTCATAGGCAATGTACCTGAGATTGCAAGTATATGATGTTAGCGTGAGTCAA
TSD
LTR1 start
Pbr_tgtt2_IT14_LTR1
Resequence
TATTAGGAAAAATAGGGAATGTAAATAATAGGAGTCCTTTATTAAGTAGGTTTGTGTATA
TATTAGGAAAAATAGGGAATGTAAATAATAGGAGTCCTTTATTAAGTAGGTTTGTGTATA
Pbr_tgtt2_IT14_LTR1
Resequence
TGTTATTTGTTTCCTTGTAAAACTATAATAGTACTGATGTATATATATTTCTAAACAAGT
TGTTATTTGTTTCCTTGTAAAACTATAATAGTACTGATGTATATATATTTCTAAACAAGT
Pbr_tgtt2_IT14_LTR1
Resequence
AATACAAGAAAATATTCCGTAAAAGTTCTATTTGGTATCAAGAGCCAAAACCCTAGTT
AATACAAGAAAATATTCCGTAAAAGTTCTATTTGGTATCAAGAGCCAAAACCCTAGTT
LTR1 end
PBS
Marker LTR1
TSD
LTR1 start
LTR1 end
PBS

Figure S2.

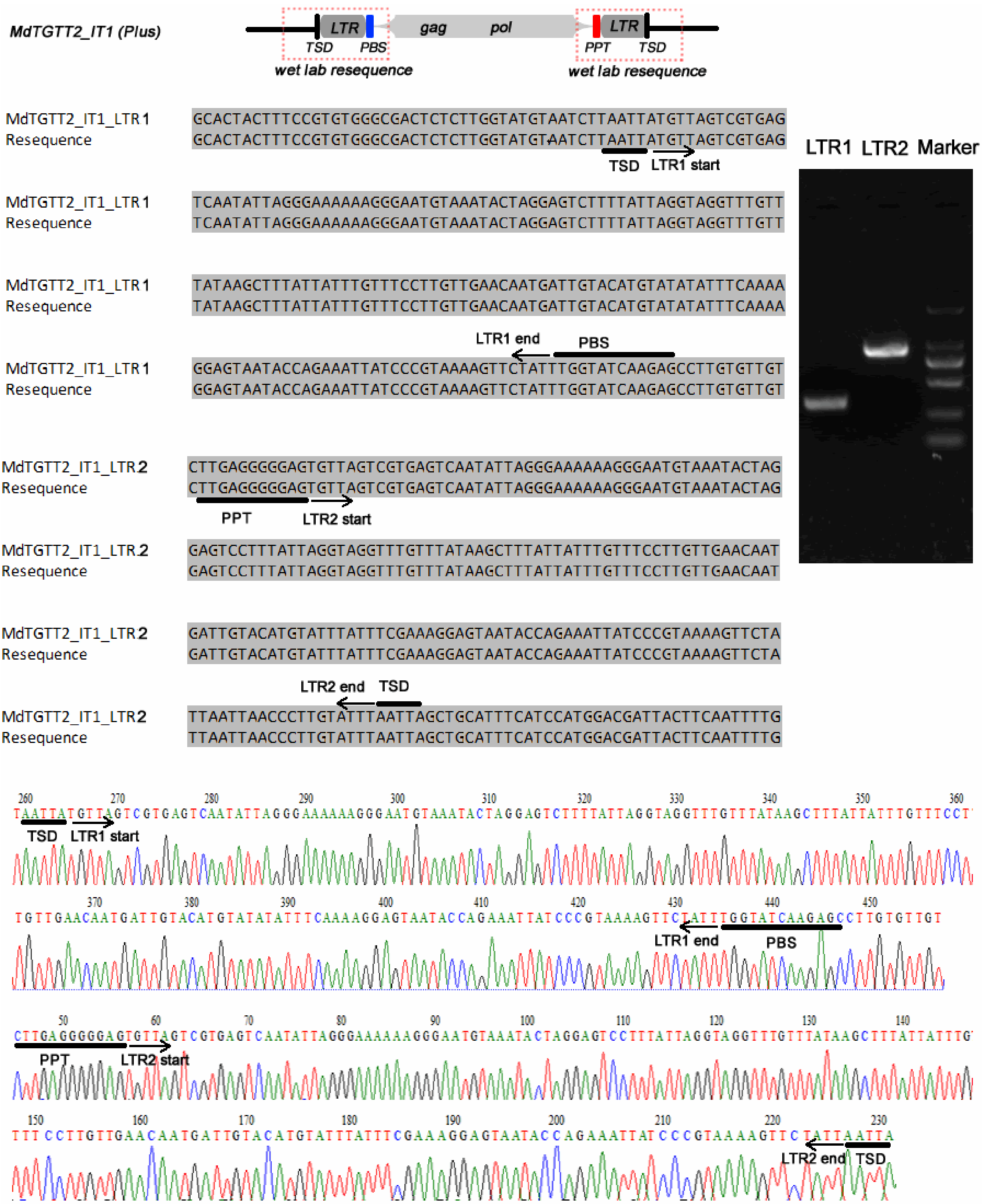
MdTGTT2_IT1 (Plus)
LTR
gag
pol
LTR
TSD
PBS
PPT
TSD
wet lab resequence
wet lab resequence
LTR1 LTR2 Marker
MdTGTT2_IT1_LTR1 GCACTACTTTCCGTGTGGGCGACTCTCTTGGTATGTAATCTTAATTATGTTAGTCGTGAG
Resequence GCACTACTTTCCGTGTGGGCGACTCTCTTGGTATGTAATCTTAATTATGTTAGTCGTGAG
TSD
LTR1 start
MdTGTT2_IT1_LTR1 TCAATATTAGGGAAAAAAGGGAATGTAAATACTAGGAGTCTTTTATTAGGTAGGTTTGTT
Resequence TCAATATTAGGGAAAAAAGGGAATGTAAATACTAGGAGTCTTTTATTAGGTAGGTTTGTT
MdTGTT2_IT1_LTR1 TATAAGCTTTATTATTTGTTTCCTTGTTGAACAATGATTGTACATGTATATATTTCAAAA
Resequence TATAAGCTTTATTATTTGTTTCCTTGTTGAACAATGATTGTACATGTATATATTTCAAAA
LTR1 end
PBS
MdTGTT2_IT1_LTR1 GGAGTAATACCAGAAATTATCCCGTAAAAGTTCTATTTGGTATCAAGAGCCTTGTGTTGT
Resequence GGAGTAATACCAGAAATTATCCCGTAAAAGTTCTATTTGGTATCAAGAGCCTTGTGTTGT
MdTGTT2_IT1_LTR2 CTTGAGGGGGAGTGTTAGTCGTGAGTCAATATTAGGGAAAAAAGGGAATGTAAATACTAG
Resequence CTTGAGGGGGAGTGTTAGTCGTGAGTCAATATTAGGGAAAAAAGGGAATGTAAATACTAG
PPT
LTR2 start
MdTGTT2_IT1_LTR2 GAGTCCTTTATTAGGTAGGTTTGTTTATAAGCTTTATTATTTGTTTCCTTGTTGAACAAT
Resequence GAGTCCTTTATTAGGTAGGTTTGTTTATAAGCTTTATTATTTGTTTCCTTGTTGAACAAT
MdTGTT2_IT1_LTR2 GATTGTACATGTATTTATTTCGAAAGGAGTAATACCAGAAATTATCCCGTAAAAGTTCTA
Resequence GATTGTACATGTATTTATTTCGAAAGGAGTAATACCAGAAATTATCCCGTAAAAGTTCTA
LTR2 end
TSD
MdTGTT2_IT1_LTR2 TTAATTAACCCTTGTATTTAATTAGCTGCATTTCATCCATGGACGATTACTTCAATTTTG
Resequence TTAATTAACCCTTGTATTTAATTAGCTGCATTTCATCCATGGACGATTACTTCAATTTTG
TSD
LTR1 start
LTR1 end
PBS
PPT
LTR2 start
LTR2 end
TSD

Figure S2.

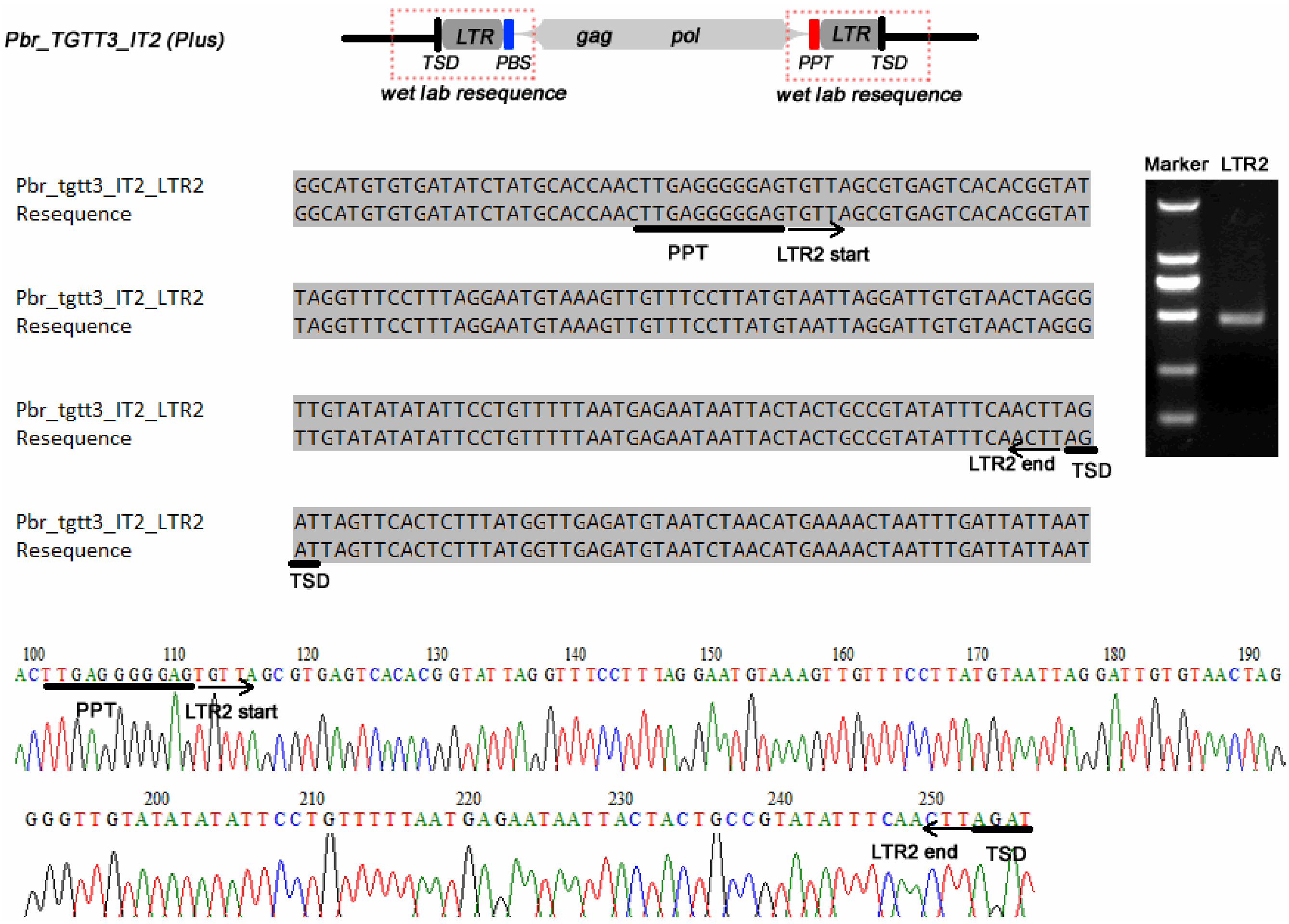
Pbr_TGTT3_IT2 (Plus)
LTR
TSD
PBS
gag
pol
LTR
PPT
TSD
wet lab resequence
wet lab resequence
Pbr_tgtt3_IT2_LTR2
Resequence
GGCATGTGTGATATCTATGCACCAACTTGAGGGGGAGTGTTAGCGTGAGTCACACGGTAT
GGCATGTGTGATATCTATGCACCAACTTGAGGGGGAGTGTTAGCGTGAGTCACACGGTAT
PPT
LTR2 start
Pbr_tgtt3_IT2_LTR2
Resequence
TAGGTTTCCTTTAGGAATGTAAAGTTGTTTCCTTATGTAATTAGGATTGTGTAACTAGGG
TAGGTTTCCTTTAGGAATGTAAAGTTGTTTCCTTATGTAATTAGGATTGTGTAACTAGGG
Pbr_tgtt3_IT2_LTR2
Resequence
TTGTATATATATTCCTGTTTTTAATGAGAATAATTACTACTGCCGTATATTTCAACTTAG
TTGTATATATATTCCTGTTTTTAATGAGAATAATTACTACTGCCGTATATTTCAACTTAG
LTR2 end
TSD
Pbr_tgtt3_IT2_LTR2
Resequence
ATTAGTTCACTCTTTATGGTTGAGATGTAATCTAACATGAAAACTAATTTGATTATTAAT
ATTAGTTCACTCTTTATGGTTGAGATGTAATCTAACATGAAAACTAATTTGATTATTAAT
TSD
Marker
LTR2
PPT
LTR2 start
LTR2 end
TSD

Figure S2.

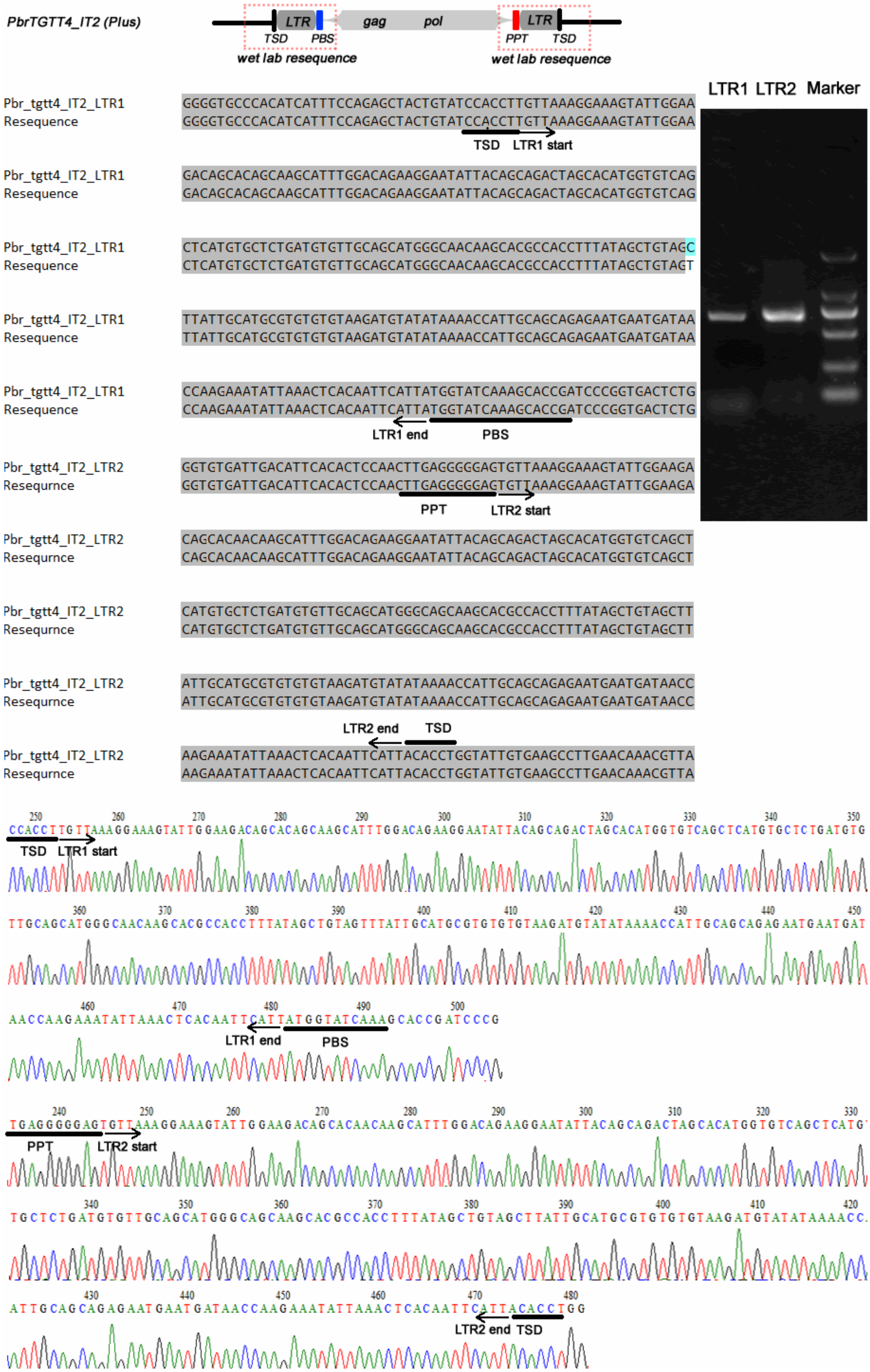

PbrTGTT4_IT2 (Plus)
LTR
TSD
PBS
gag
pol
LTR
PPT
TSD
wet lab resequence
wet lab resequence
LTR1 LTR2 Marker
Pbr_tgtt4_IT2_LTR1 GGGGTGCCCACATCATTTCCAGAGCTACTGTATCCACCTTGTTAAAGGAAAGTATTGGAA
Resequence GGGGTGCCCACATCATTTCCAGAGCTACTGTATCCACCTTGTTAAAGGAAAGTATTGGAA
TSD LTR1 start
Pbr_tgtt4_IT2_LTR1 GACAGCACAGCAAGCATTTGGACAGAAGGAATATTACAGCAGACTAGCACATGGTGTCAG
Resequence GACAGCACAGCAAGCATTTGGACAGAAGGAATATTACAGCAGACTAGCACATGGTGTCAG
Pbr_tgtt4_IT2_LTR1 CTCATGTGCTCTGATGTGTTGCAGCATGGGCAACAAGCACGCCACCTTTATAGCTGTAGC
Resequence CTCATGTGCTCTGATGTGTTGCAGCATGGGCAACAAGCACGCCACCTTTATAGCTGTAGT
Pbr_tgtt4_IT2_LTR1 TTATTGCATGCGTGTGTGTAAGATGTATATAAAACCATTGCAGCAGAGAATGAATGATAA
Resequence TTATTGCATGCGTGTGTGTAAGATGTATATAAAACCATTGCAGCAGAGAATGAATGATAA
Pbr_tgtt4_IT2_LTR1 CCAAGAAATATTAAACTCACAATTCATTATGGTATCAAAGCACCGATCCCGGTGACTCTG
Resequence CCAAGAAATATTAAACTCACAATTCATTATGGTATCAAAGCACCGATCCCGGTGACTCTG
LTR1 end PBS
Pbr_tgtt4_IT2_LTR2 GGTGTGATTGACATTCACACTCCAACTTGAGGGGGAGTGTTAAAGGAAAGTATTGGAAGA
Resequrnce GGTGTGATTGACATTCACACTCCAACTTGAGGGGGAGTGTTAAAGGAAAGTATTGGAAGA
PPT LTR2 start
Pbr_tgtt4_IT2_LTR2 CAGCACAACAAGCATTTGGACAGAAGGAATATTACAGCAGACTAGCACATGGTGTCAGCT
Resequrnce CAGCACAACAAGCATTTGGACAGAAGGAATATTACAGCAGACTAGCACATGGTGTCAGCT
Pbr_tgtt4_IT2_LTR2 CATGTGCTCTGATGTGTTGCAGCATGGGCAGCAAGCACGCCACCTTTATAGCTGTAGCTT
Resequrnce CATGTGCTCTGATGTGTTGCAGCATGGGCAGCAAGCACGCCACCTTTATAGCTGTAGCTT
Pbr_tgtt4_IT2_LTR2 ATTGCATGCGTGTGTGTAAGATGTATATAAAACCATTGCAGCAGAGAATGAATGATAACC
Resequrnce ATTGCATGCGTGTGTGTAAGATGTATATAAAACCATTGCAGCAGAGAATGAATGATAACC
LTR2 end TSD
Pbr_tgtt4_IT2_LTR2 AAGAAATATTAAACTCACAATTCATTACACCTGGTATTGTGAAGCCTTGAACAAACGTTA
Resequrnce AAGAAATATTAAACTCACAATTCATTACACCTGGTATTGTGAAGCCTTGAACAAACGTTA
TSD LTR1 start
LTR1 end PBS
PPT LTR2 start
LTR2 end TSD

Figure S2.

PbrAACA1_IT3 (Plus)

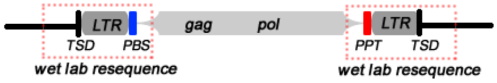

Pbr_aaca1_IT3_LTR1 CTTGTACAGAGCTGCTTGTAAGAAAGTTAATATTAAAGATGAAGATGCTATATCATAACT
Resequence CTTGTACAGAGCTGCTTGTAAGAAAGTTAATATTAAAGATGAAGATGCTATATCATAACT

TSD LTR1 start

Pbr_aaca1_IT3_LTR1 ACAGTTGTTAGTTATTAGTTTGGTATAAGGTGTCATTCATGTAATTAGTTTATCTTATGT
Resequence ACAGTTGTTAGTTATTAGTTTGGTATAAGGTGTCATTCATGTAATTAGTTTATCTTATGT

Pbr_aaca1_IT3_LTR1 GGTAGCTTAGAATAGCTAAATGTAAATAGTCTAAAGACTATATATATGTGATTGGATTAT
Resequence GGTAGCTTAGAATAGCTAAATGTAAATAGTCTAAAGACTATATATATGTGATTGGATTAT

Pbr_aaca1_IT3_LTR1 TATTTTGGAATAATGAGAATACACAGAAATCTTAATACTTAAGAAATCTTTCTTTCTCGC
Resequence TATTTTGGAATAATGAGAATACACAGAAATCTTAATACTTAAGAAATCTTTCTTTCTCGC

Pbr_aaca1_IT3_LTR1 TCTTCTCTCTTCTTTCTTCTCTCTCTCTTTCTTTTGCAAGAACACAGAACTTCTACATGG
Resequence TCTTCTCTCTTCTTTCTTCTCTCTCTCTTTCTTTTGCAAGAACACAGAACTTCTACATGG

LTR1 end PBS

Pbr_aaca1_IT3_LTR1 TATCCTTCGCCGCTTGCCTCACGGCCATTTCGATCCATTCTTCCGCTTTGTTTCTCTGAT
Resequence TATCCTTCGCCGCTTGCCTCACGGCCATTTCGATCCATTCTTCCGCTTTGTTTCTCTGAT

PBS

Pbr_aaca1_IT3_LTR2 AATTCTTAATTCTTCCAAGTTTAGCTTGAGGGGGTGTAAAGATGAAGATGCTATATCATA
Resequence AATTCTTAATTCTTCCAAGTTTAGCTTGAGGGGGTGTAAAGATGAAGATGCTATATCATA

PPT LTR2 start

Pbr_aaca1_IT3_LTR2 ACTACAGTTGTTAGTTATTAGTTTGGTATAAGGTGTCATTCATGTAATTAGTTTATCTTA
Resequence ACTACAGTTGTTAGTTATTAGTTTGGTATAAGGTGTCATTCATGTAATTAGTTTATCTTA

Pbr_aaca1_IT3_LTR2 TGTGGTAGCTTAGAATAGCTAAATGTAAATAGTCTAAAGACTATATATATGTGGATTGGA
Resequence TGTGGTAGCTTAGAATAGCTAAATGTAAATAGTCTAAAGACTATATATATGT. GATTGGA

Pbr_aaca1_IT3_LTR2 TTATTATTTTGGAATAATGAGAATACACAGAAATCTTAATACTTAAGAAATCTTTCTTTC
Resequence TTATTATTTTGGAATAATGAGAATACACAGAAATCTTAATACTTAAGAAATCTTTCTTTC

LTR2 end

Pbr_aaca1_IT3_LTR2 TCGCTCTTCTCTCTTCTTTCTTCTCTCTCTCTTTCTTTTGCAAGAACACAGAACTTCTAC
Resequence TCGCTCTTCTCTCTTCTTTCTTCTCTCTCTCTTTCTTTTGCAAGAACACAGAACTTCTAC

LTR2 end TSD

Pbr_aaca1_IT3_LTR2 AAATATAGTTTGCTTTGTTTCGTAATGGTTGTGGGGAAGAAAGTTAATATATACACCTTC
Resequence AAATATAGTTTGCTTTGTTTCGTAATGGTTGTGGGGAAGAAAGTTAATATATACACCTTC

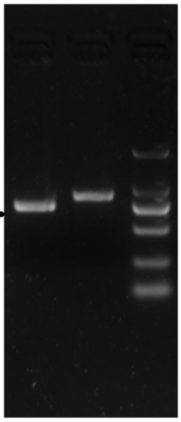

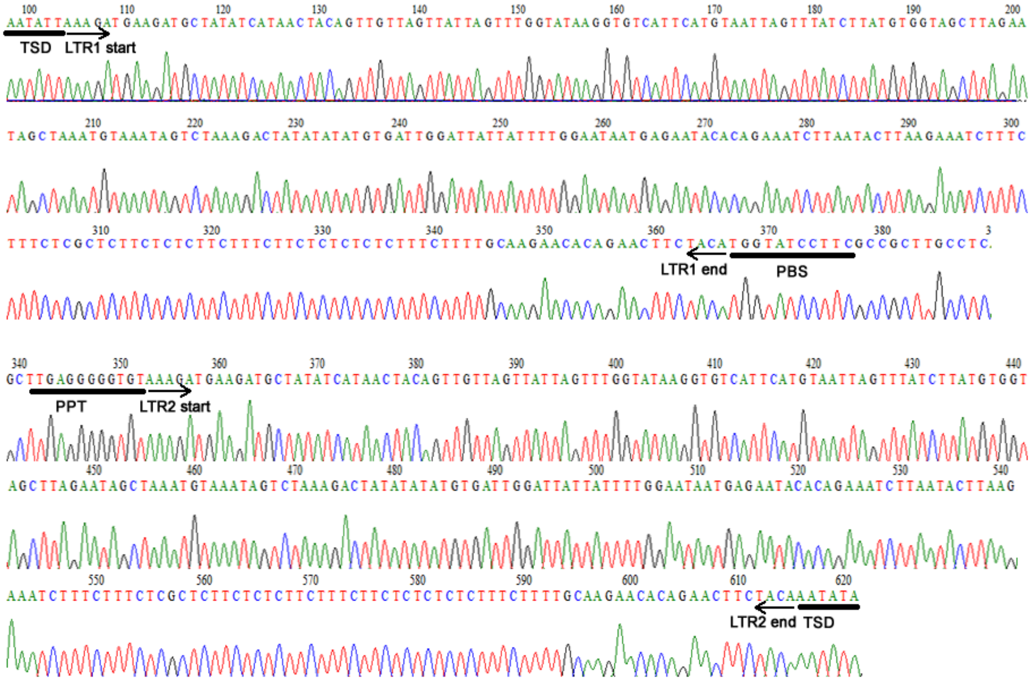

Figure S2.

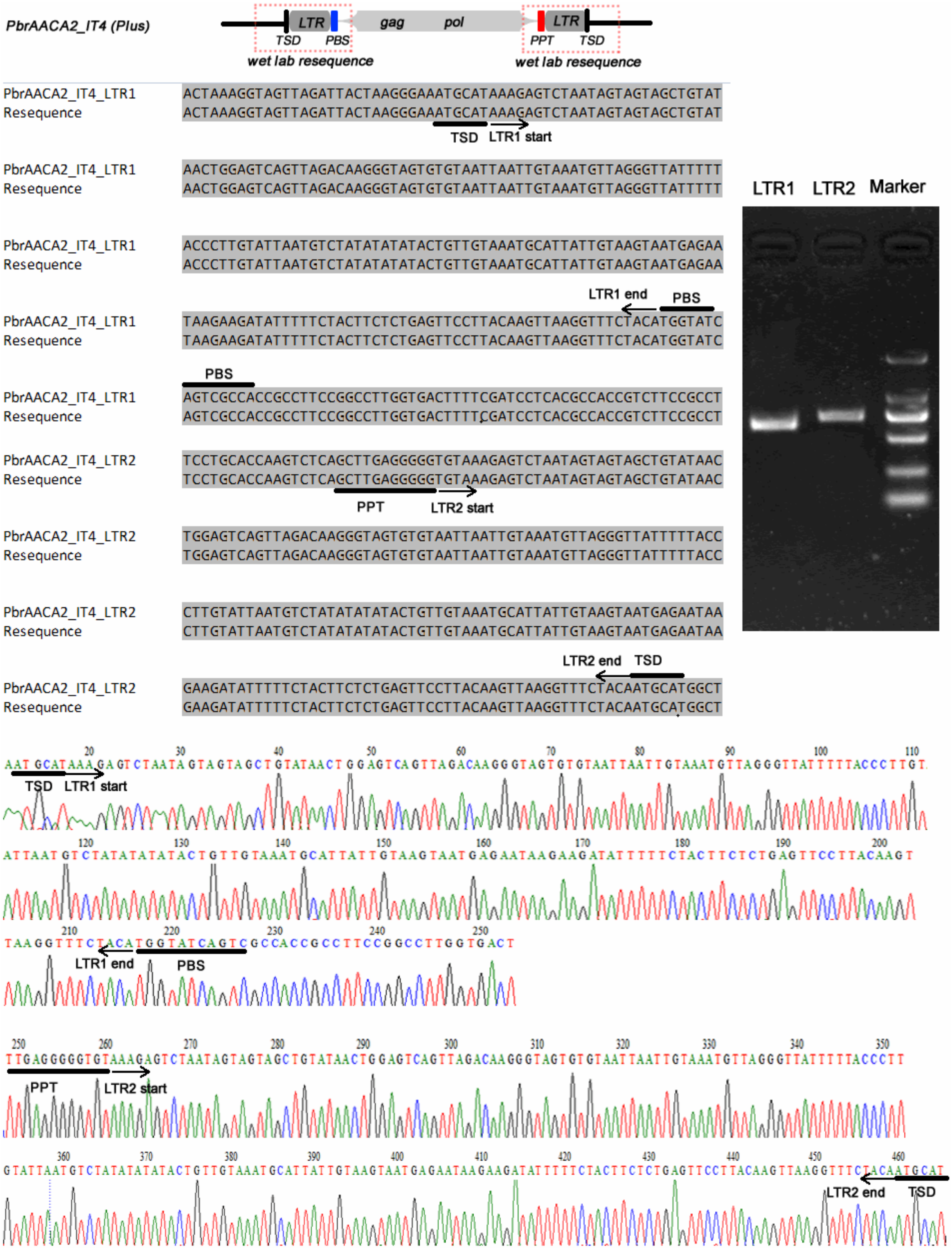

PbrAACA2_IT4 (Plus)
LTR
gag
pol
LTR
TSD
PBS
PPT
TSD
wet lab resequence
wet lab resequence
PbrAACA2_IT4_LTR1 ACTAAAGGTAGTTAGATTACTAAGGGAAATGCATAAAGAGTCTAATAGTAGTAGCTGTAT
Resequence ACTAAAGGTAGTTAGATTACTAAGGGAAATGCATAAAGAGTCTAATAGTAGTAGCTGTAT
TSD
LTR1 start
PbrAACA2_IT4_LTR1 AACTGGAGTCAGTTAGACAAGGGTAGTGTGTAATTAATTGTAAATGTTAGGGTTATTTTT
Resequence AACTGGAGTCAGTTAGACAAGGGTAGTGTGTAATTAATTGTAAATGTTAGGGTTATTTTT
PbrAACA2_IT4_LTR1 ACCCTTGTATTAATGTCTATATATATACTGTTGTAAATGCATTATTGTAAGTAATGAGAA
Resequence ACCCTTGTATTAATGTCTATATATATACTGTTGTAAATGCATTATTGTAAGTAATGAGAA
LTR1 end
PBS
PbrAACA2_IT4_LTR1 TAAGAAGATATTTTTCTACTTCTCTGAGTTCCTTACAAGTTAAGGTTTCTACATGGTATC
Resequence TAAGAAGATATTTTTCTACTTCTCTGAGTTCCTTACAAGTTAAGGTTTCTACATGGTATC
PBS
PbrAACA2_IT4_LTR1 AGTCGCCACCGCCTTCCGGCCTTGGTGACTTTTCGATCCTCACGCCACCGTCTTCCGCCT
Resequence AGTCGCCACCGCCTTCCGGCCTTGGTGACTTTTCGATCCTCACGCCACCGTCTTCCGCCT
PbrAACA2_IT4_LTR2 TCCTGCACCAAGTCTCAGCTTGAGGGGGTGTAAAGAGTCTAATAGTAGTAGCTGTATAAC
Resequence TCCTGCACCAAGTCTCAGCTTGAGGGGGTGTAAAGAGTCTAATAGTAGTAGCTGTATAAC
PPT
LTR2 start
PbrAACA2_IT4_LTR2 TGGAGTCAGTTAGACAAGGGTAGTGTGTAATTAATTGTAAATGTTAGGGTTATTTTTACC
Resequence TGGAGTCAGTTAGACAAGGGTAGTGTGTAATTAATTGTAAATGTTAGGGTTATTTTTACC
PbrAACA2_IT4_LTR2 CTTGTATTAATGTCTATATATATACTGTTGTAAATGCATTATTGTAAGTAATGAGAATAA
Resequence CTTGTATTAATGTCTATATATATACTGTTGTAAATGCATTATTGTAAGTAATGAGAATAA
LTR2 end
TSD
PbrAACA2_IT4_LTR2 GAAGATATTTTTCTACTTCTCTGAGTTCCTTACAAGTTAAGGTTTCTACAATGCATGGCT
Resequence GAAGATATTTTTCTACTTCTCTGAGTTCCTTACAAGTTAAGGTTTCTACAATGCATGGCT
LTR1
LTR2
Marker
TSD
LTR1 start
LTR1 end
PBS
PPT
LTR2 start
LTR2 end
TSD

Figure S2.

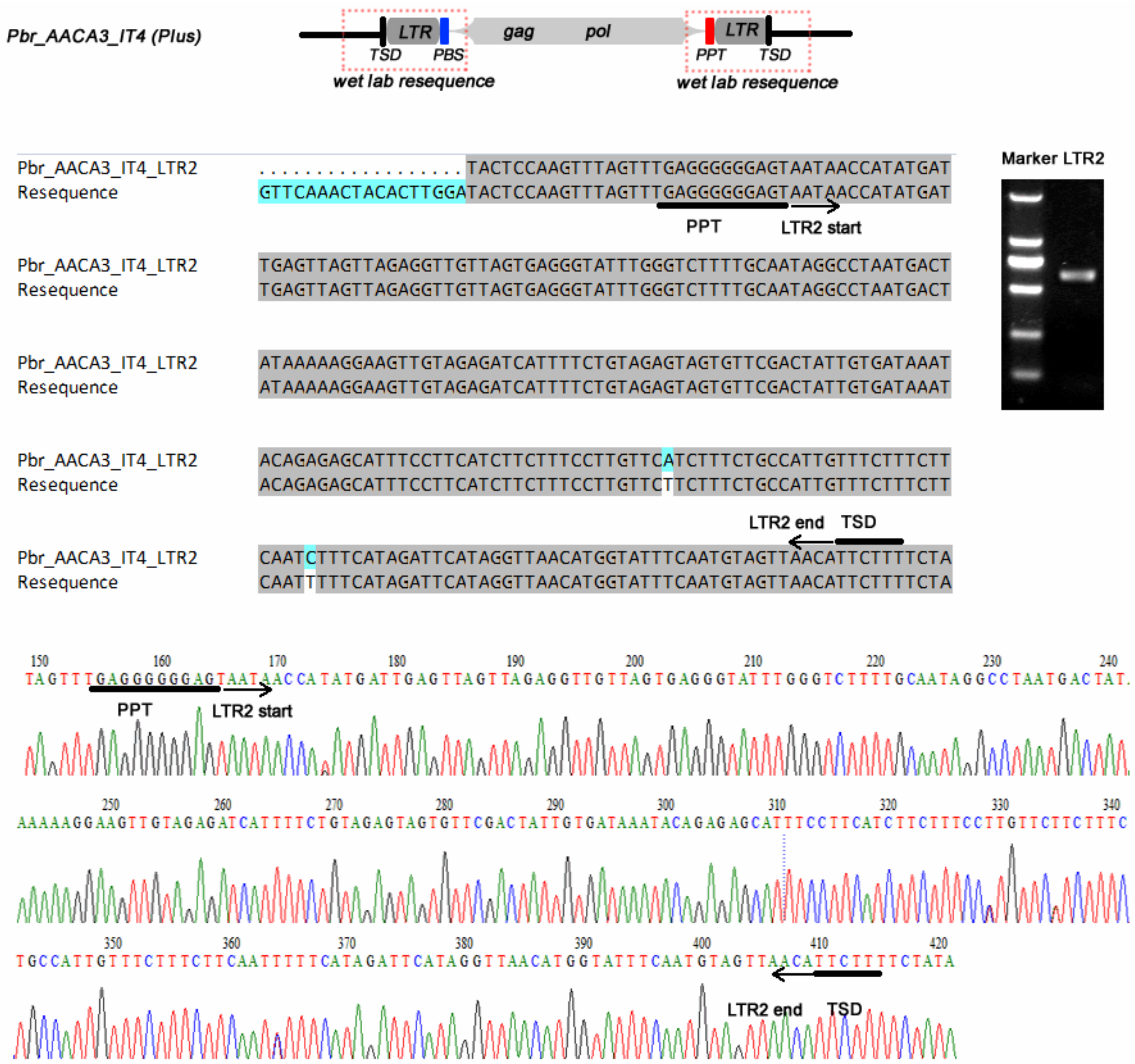

Pbr_AACA3_IT4 (Plus)
LTR
gag
pol
LTR
TSD
PBS
PPT
TSD
wet lab resequence
wet lab resequence
Pbr_AACA3_IT4_LTR2 ..................TACTCCAAGTTTAGTTTGAGGGGGGAGTAATAACCATATGAT
Resequence GTTCAAACTACACTTGGATACTCCAAGTTTAGTTTGAGGGGGGAGTAATAACCATATGAT
PPT
LTR2 start
Pbr_AACA3_IT4_LTR2 TGAGTTAGTTAGAGGTTGTTAGTGAGGGTATTTGGGTCTTTTGCAATAGGCCTAATGACT
Resequence TGAGTTAGTTAGAGGTTGTTAGTGAGGGTATTTGGGTCTTTTGCAATAGGCCTAATGACT
Pbr_AACA3_IT4_LTR2 ATAAAAAGGAAGTTGTAGAGATCATTTTCTGTAGAGTAGTGTTCGACTATTGTGATAAAT
Resequence ATAAAAAGGAAGTTGTAGAGATCATTTTCTGTAGAGTAGTGTTCGACTATTGTGATAAAT
Pbr_AACA3_IT4_LTR2 ACAGAGAGCATTTCCTTCATCTTCTTTCCTTGTTCATCTTTCTGCCATTGTTTCTTTCTT
Resequence ACAGAGAGCATTTCCTTCATCTTCTTTCCTTGTTCTTCTTTCTGCCATTGTTTCTTTCTT
LTR2 end
TSD
Pbr_AACA3_IT4_LTR2 CAATCTTTCATAGATTCATAGGTTAACATGGTATTTCAATGTAGTTAACATTCTTTTCTA
Resequence CAATTTTTCATAGATTCATAGGTTAACATGGTATTTCAATGTAGTTAACATTCTTTTCTA
Marker LTR2
PPT
LTR2 start
LTR2 end
TSD

Figure S2.

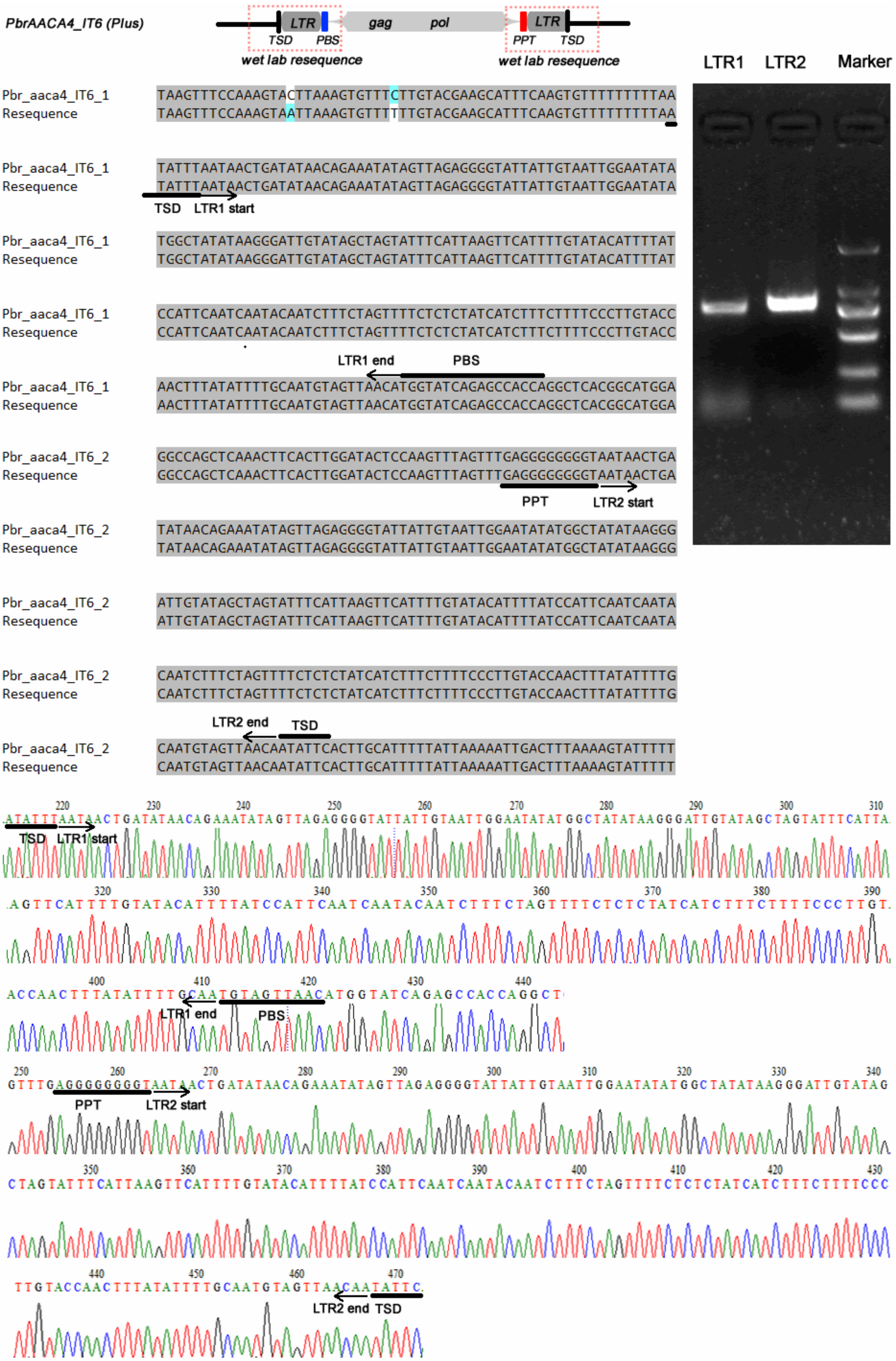

PbrAACA4_IT6 (Plus)
LTR
TSD
PBS
gag
pol
LTR
PPT
TSD
wet lab resequence
wet lab resequence
LTR1
LTR2
Marker
Pbr_aaca4_IT6_1 TAAGTTTCCAAAGTACTTAAAGTGTTTCTTGTACGAAGCATTTCAAGTGTTTTTTTTTAA
Resequence TAAGTTTCCAAAGTAATTAAAGTGTTTTTTGTACGAAGCATTTCAAGTGTTTTTTTTTAA
Pbr_aaca4_IT6_1 TATTTAATAACTGATATAACAGAAATATAGTTAGAGGGGTATTATTGTAATTGGAATATA
Resequence TATTTAATAACTGATATAACAGAAATATAGTTAGAGGGGTATTATTGTAATTGGAATATA
TSD
LTR1 start
Pbr_aaca4_IT6_1 TGGCTATATAAGGGATTGTATAGCTAGTATTTCATTAAGTTCATTTTGTATACATTTTAT
Resequence TGGCTATATAAGGGATTGTATAGCTAGTATTTCATTAAGTTCATTTTGTATACATTTTAT
Pbr_aaca4_IT6_1 CCATTCAATCAATACAATCTTTCTAGTTTTCTCTCTATCATCTTTCTTTTCCCTTGTACC
Resequence CCATTCAATCAATACAATCTTTCTAGTTTTCTCTCTATCATCTTTCTTTTCCCTTGTACC
LTR1 end
PBS
Pbr_aaca4_IT6_1 AACTTTATATTTTGCAATGTAGTTAACATGGTATCAGAGCCACCAGGCTCACGGCATGGA
Resequence AACTTTATATTTTGCAATGTAGTTAACATGGTATCAGAGCCACCAGGCTCACGGCATGGA
Pbr_aaca4_IT6_2 GGCCAGCTCAAACTTCACTTGGATACTCCAAGTTTAGTTTGAGGGGGGGGTAATAACTGA
Resequence GGCCAGCTCAAACTTCACTTGGATACTCCAAGTTTAGTTTGAGGGGGGGGTAATAACTGA
PPT
LTR2 start
Pbr_aaca4_IT6_2 TATAACAGAAATATAGTTAGAGGGGTATTATTGTAATTGGAATATATGGCTATATAAGGG
Resequence TATAACAGAAATATAGTTAGAGGGGTATTATTGTAATTGGAATATATGGCTATATAAGGG
Pbr_aaca4_IT6_2 ATTGTATAGCTAGTATTTCATTAAGTTCATTTTGTATACATTTTATCCATTCAATCAATA
Resequence ATTGTATAGCTAGTATTTCATTAAGTTCATTTTGTATACATTTTATCCATTCAATCAATA
Pbr_aaca4_IT6_2 CAATCTTTCTAGTTTTCTCTCTATCATCTTTCTTTTCCCTTGTACCAACTTTATATTTTG
Resequence CAATCTTTCTAGTTTTCTCTCTATCATCTTTCTTTTCCCTTGTACCAACTTTATATTTTG
LTR2 end
TSD
Pbr_aaca4_IT6_2 CAATGTAGTTAACAATATTCACTTGCATTTTTATTAAAAATTGACTTTAAAAGTATTTTT
Resequence CAATGTAGTTAACAATATTCACTTGCATTTTTATTAAAAATTGACTTTAAAAGTATTTTT
TSD
LTR1 start
LTR1 end
PBS
PPT
LTR2 start
LTR2 end
TSD

Figure S2.

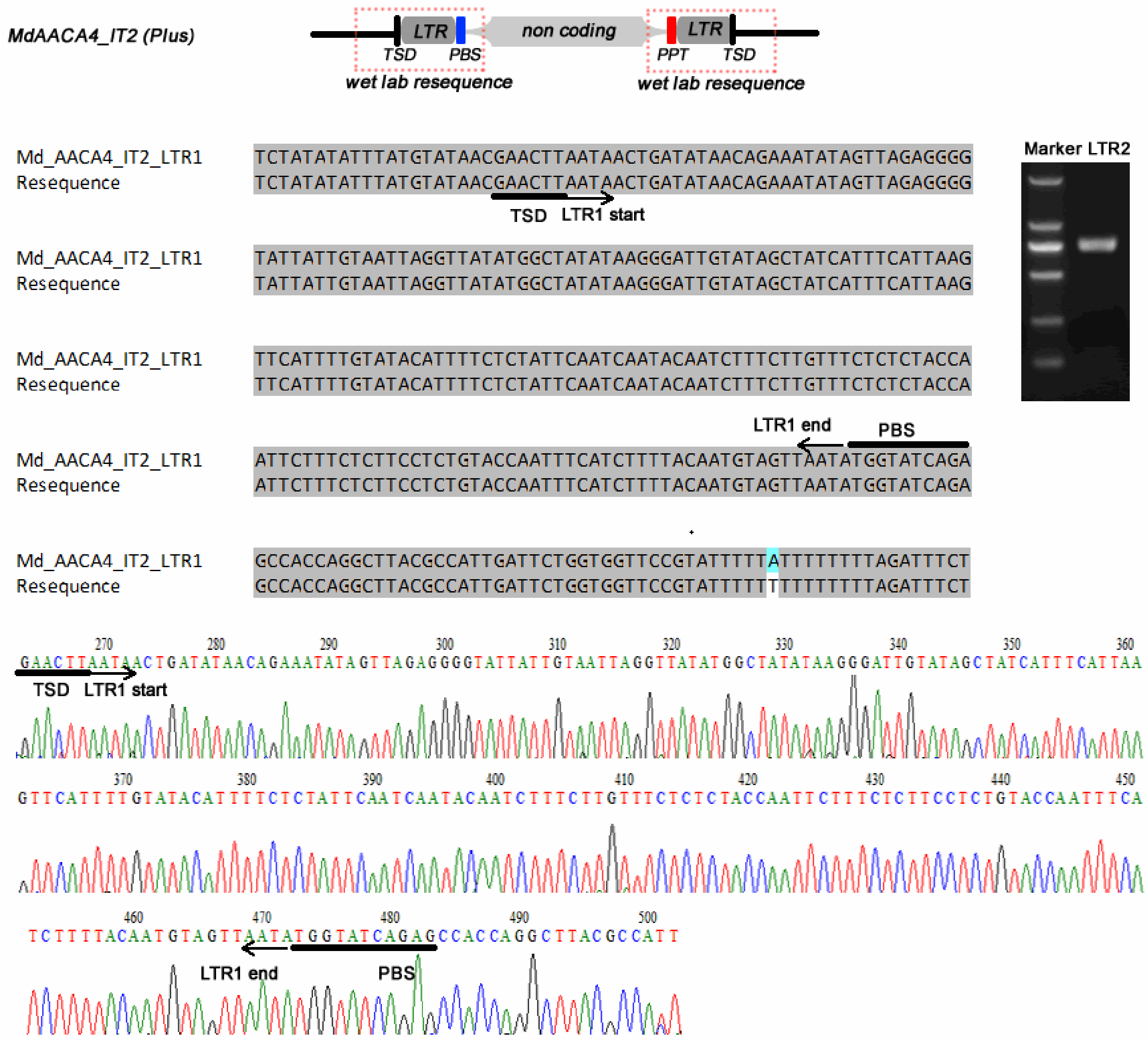
MdAACA4_IT2 (Plus)
LTR
non coding
LTR
TSD
PBS
PPT
TSD
wet lab resequence
wet lab resequence
Marker LTR2
Md_AACA4_IT2_LTR1 TCTATATATTTATGTATAACGAACTTAATAACTGATATAACAGAAATATAGTTAGAGGGG
Resequence TCTATATATTTATGTATAACGAACTTAATAACTGATATAACAGAAATATAGTTAGAGGGG
TSD LTR1 start
Md_AACA4_IT2_LTR1 TATTATTGTAATTAGGTTATATGGCTATATAAGGGATTGTATAGCTATCATTTCATTAAG
Resequence TATTATTGTAATTAGGTTATATGGCTATATAAGGGATTGTATAGCTATCATTTCATTAAG
Md_AACA4_IT2_LTR1 TTCATTTTGTATACATTTTCTCTATTCAATCAATACAATCTTTCTTGTTTCTCTCTACCA
Resequence TTCATTTTGTATACATTTTCTCTATTCAATCAATACAATCTTTCTTGTTTCTCTCTACCA
LTR1 end
PBS
Md_AACA4_IT2_LTR1 ATTCTTTCTCTTCCTCTGTACCAATTTCATCTTTTACAATGTAGTTAATATGGTATCAGA
Resequence ATTCTTTCTCTTCCTCTGTACCAATTTCATCTTTTACAATGTAGTTAATATGGTATCAGA
Md_AACA4_IT2_LTR1 GCCACCAGGCTTACGCCATTGATTCTGGTGGTTCCGTATTTTTATTTTTTTTAGATTTCT
Resequence GCCACCAGGCTTACGCCATTGATTCTGGTGGTTCCGTATTTTTTTTTTTTTTAGATTTCT
TSD LTR1 start
LTR1 end
PBS

Figure S3.

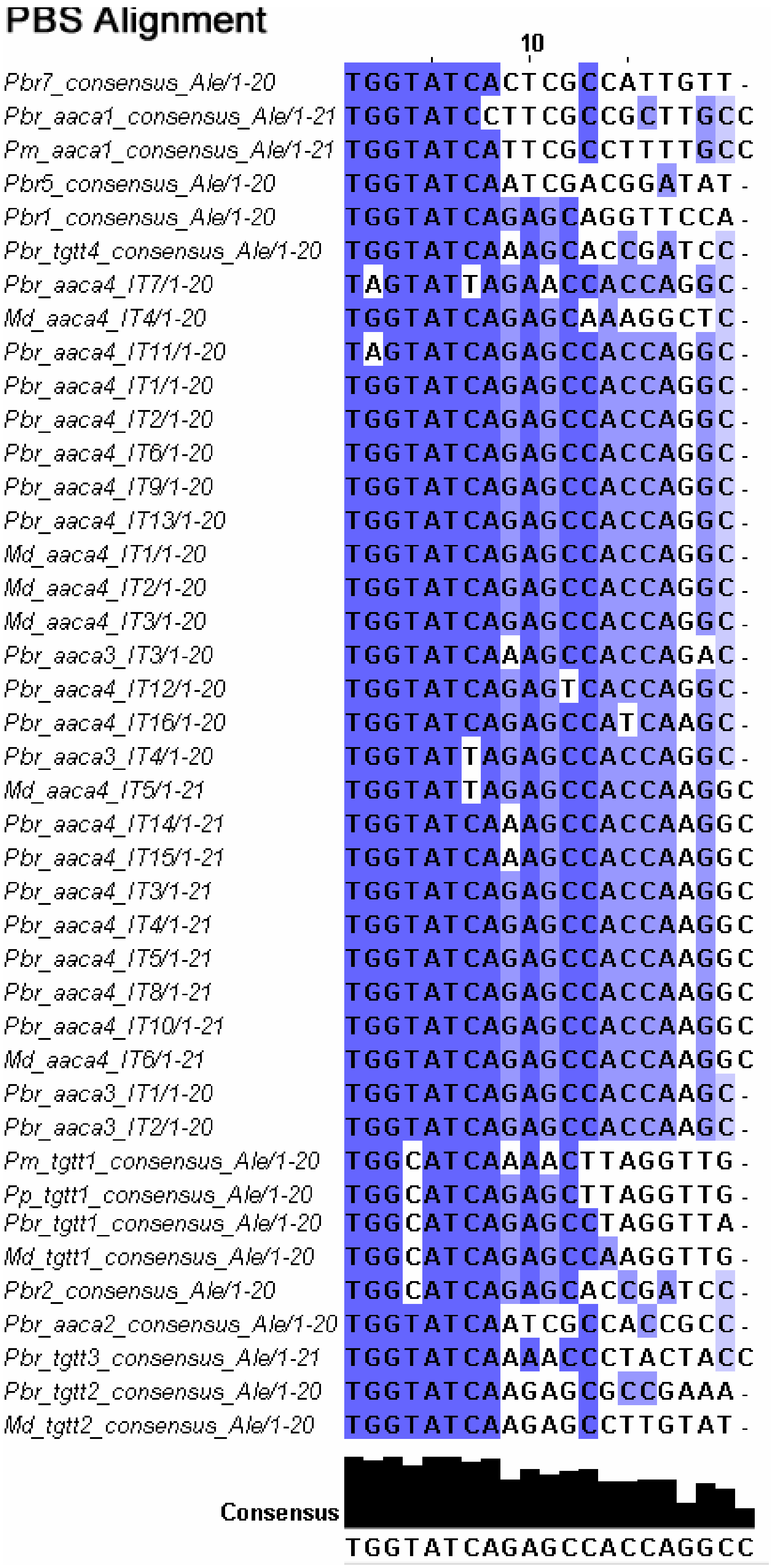
PBS Alignment
10
Pbr7_consensus_Ale/1-20 TGGTATCACTCGCCATTGTT-
Pbr_aaca1_consensus_Ale/1-21 TGGTATCCTTCGCCGCTTGCC
Pm_aaca1_consensus_Ale/1-21 TGGTATCATTCGCCTTTTGCC
Pbr5_consensus_Ale/1-20 TGGTATCAATCGACGGATAT-
Pbr1_consensus_Ale/1-20 TGGTATCAGAGCAGGTTCCA-
Pbr_tgtt4_consensus_Ale/1-20 TGGTATCAAAGCACCGATCC-
Pbr_aaca4_IT7/1-20 TAGTATTAGAACCACCAGGC-
Md_aaca4_IT4/1-20 TGGTATCAGAGCAAAGGCTC-
Pbr_aaca4_IT11/1-20 TAGTATCAGAGCCACCAGGC-
Pbr_aaca4_IT1/1-20 TGGTATCAGAGCCACCAGGC-
Pbr_aaca4_IT2/1-20 TGGTATCAGAGCCACCAGGC-
Pbr_aaca4_IT6/1-20 TGGTATCAGAGCCACCAGGC-
Pbr_aaca4_IT9/1-20 TGGTATCAGAGCCACCAGGC-
Pbr_aaca4_IT13/1-20 TGGTATCAGAGCCACCAGGC-
Md_aaca4_IT1/1-20 TGGTATCAGAGCCACCAGGC-
Md_aaca4_IT2/1-20 TGGTATCAGAGCCACCAGGC-
Md_aaca4_IT3/1-20 TGGTATCAGAGCCACCAGGC-
Pbr_aaca3_IT3/1-20 TGGTATCAAAGCCACCAGAC-
Pbr_aaca4_IT12/1-20 TGGTATCAGAGTCACCAGGC-
Pbr_aaca4_IT16/1-20 TGGTATCAGAGCCATCAAGC-
Pbr_aaca3_IT4/1-20 TGGTATTAGAGCCACCAGGC-
Md_aaca4_IT5/1-21 TGGTATTAGAGCCACCAAGGC
Pbr_aaca4_IT14/1-21 TGGTATCAAAGCCACCAAGGC
Pbr_aaca4_IT15/1-21 TGGTATCAAAGCCACCAAGGC
Pbr_aaca4_IT3/1-21 TGGTATCAGAGCCACCAAGGC
Pbr_aaca4_IT4/1-21 TGGTATCAGAGCCACCAAGGC
Pbr_aaca4_IT5/1-21 TGGTATCAGAGCCACCAAGGC
Pbr_aaca4_IT8/1-21 TGGTATCAGAGCCACCAAGGC
Pbr_aaca4_IT10/1-21 TGGTATCAGAGCCACCAAGGC
Md_aaca4_IT6/1-21 TGGTATCAGAGCCACCAAGGC
Pbr_aaca3_IT1/1-20 TGGTATCAGAGCCACCAAGC-
Pbr_aaca3_IT2/1-20 TGGTATCAGAGCCACCAAGC-
Pm_tgtt1_consensus_Ale/1-20 TGGCATCAAAACTTAGGTTG-
Pp_tgtt1_consensus_Ale/1-20 TGGCATCAGAGCTTAGGTTG-
Pbr_tgtt1_consensus_Ale/1-20 TGGCATCAGAGCCTAGGTTA-
Md_tgtt1_consensus_Ale/1-20 TGGCATCAGAGCCAAGGTTG-
Pbr2_consensus_Ale/1-20 TGGCATCAGAGCACCGATCC-
Pbr_aaca2_consensus_Ale/1-20 TGGTATCAATCGCCACCGCC-
Pbr_tgtt3_consensus_Ale/1-21 TGGTATCAAAACCCTACTACC
Pbr_tgtt2_consensus_Ale/1-20 TGGTATCAAGAGCGCCGAAA-
Md_tgtt2_consensus_Ale/1-20 TGGTATCAAGAGCCTTGTAT-
Consensus
TGGTATCAGAGCCACCAGGCC

Figure S4.

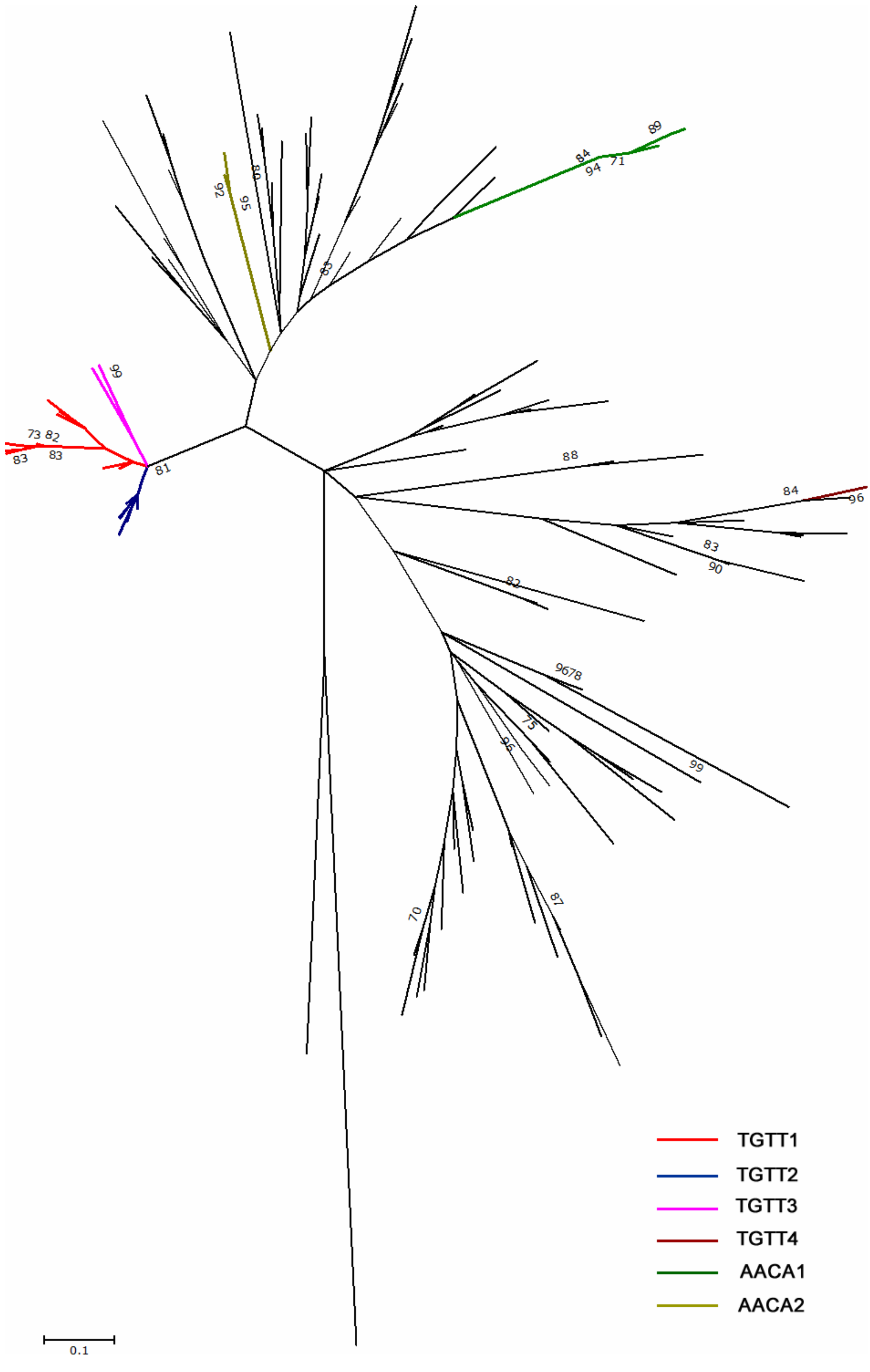

Figure S5.

PbrAACA1_IT3

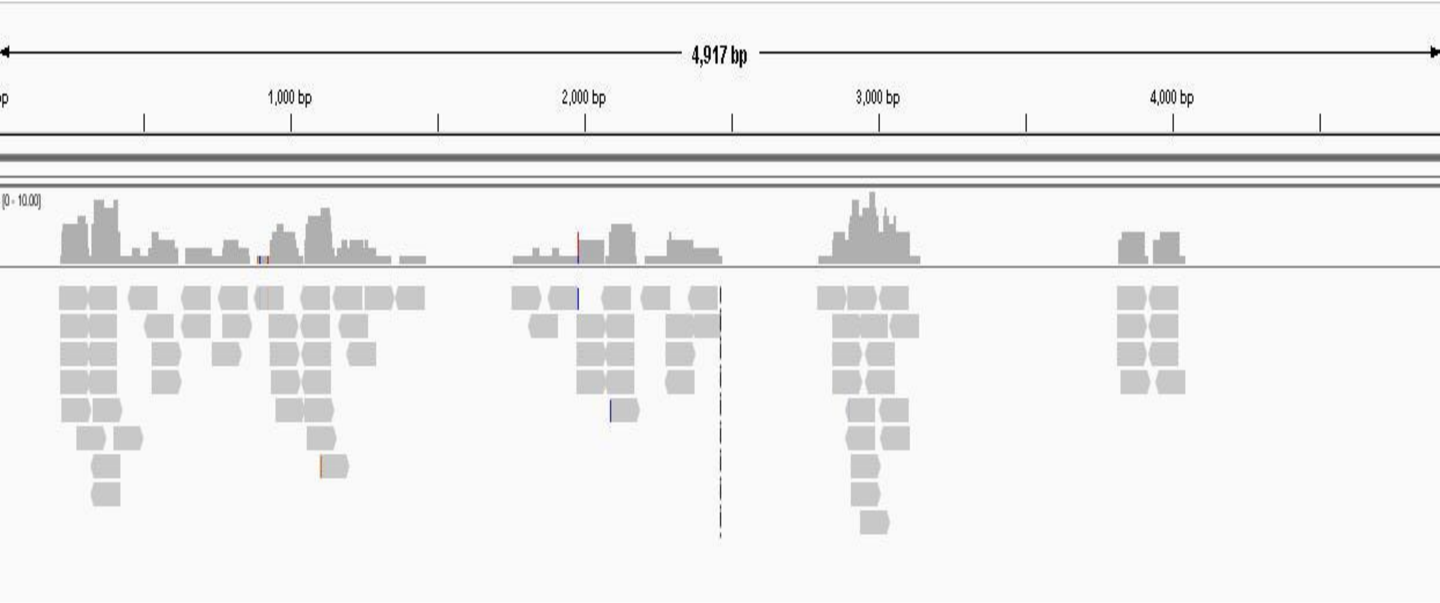

NCBI SRA database: SRX147917

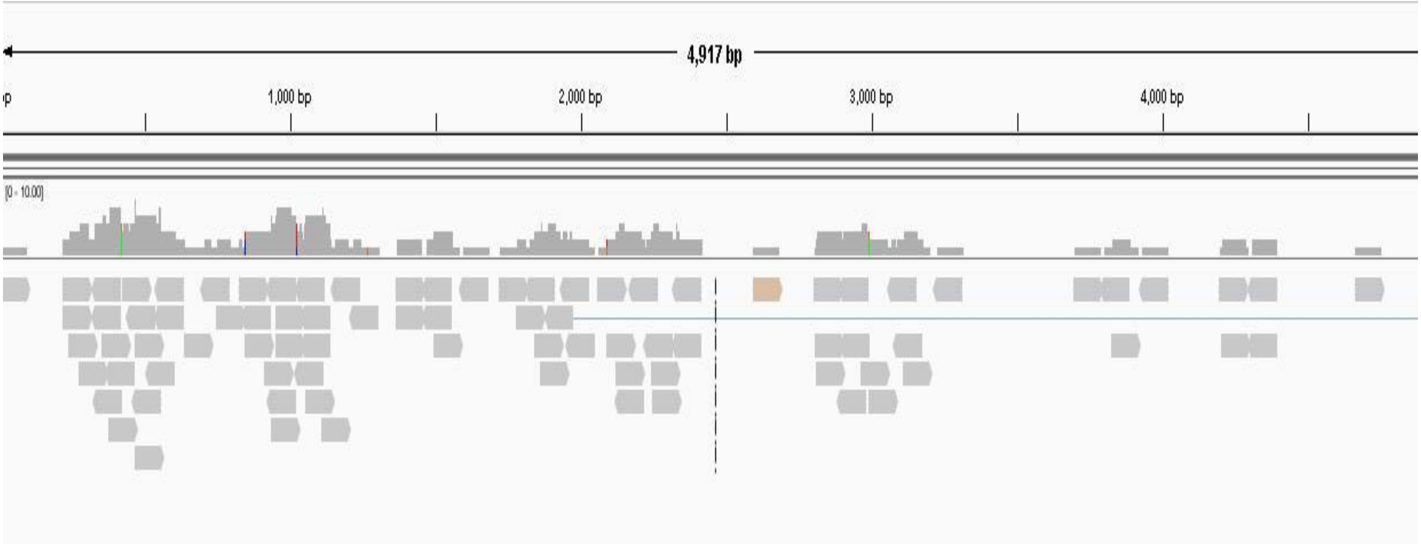

NCBI SRA database: SRX298075

Figure S5.

PbrAACA1_IT5

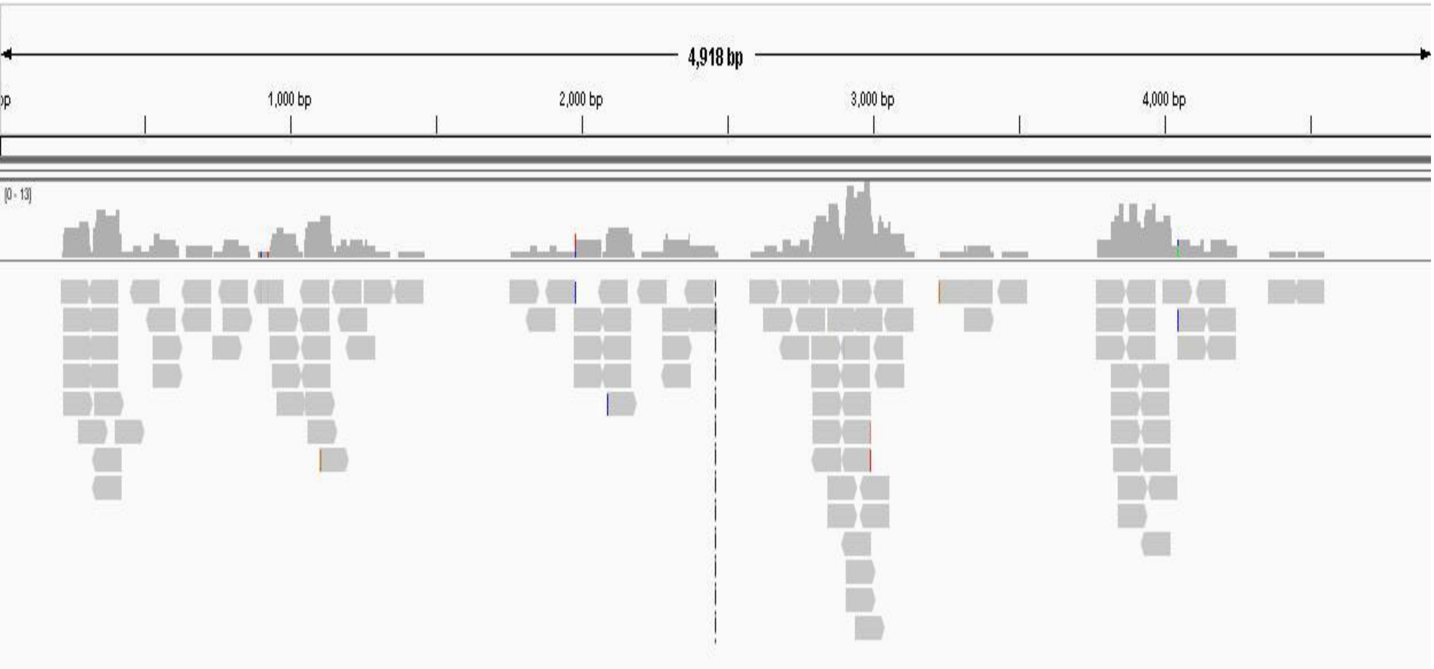

NCBI SRA database: SRX147917

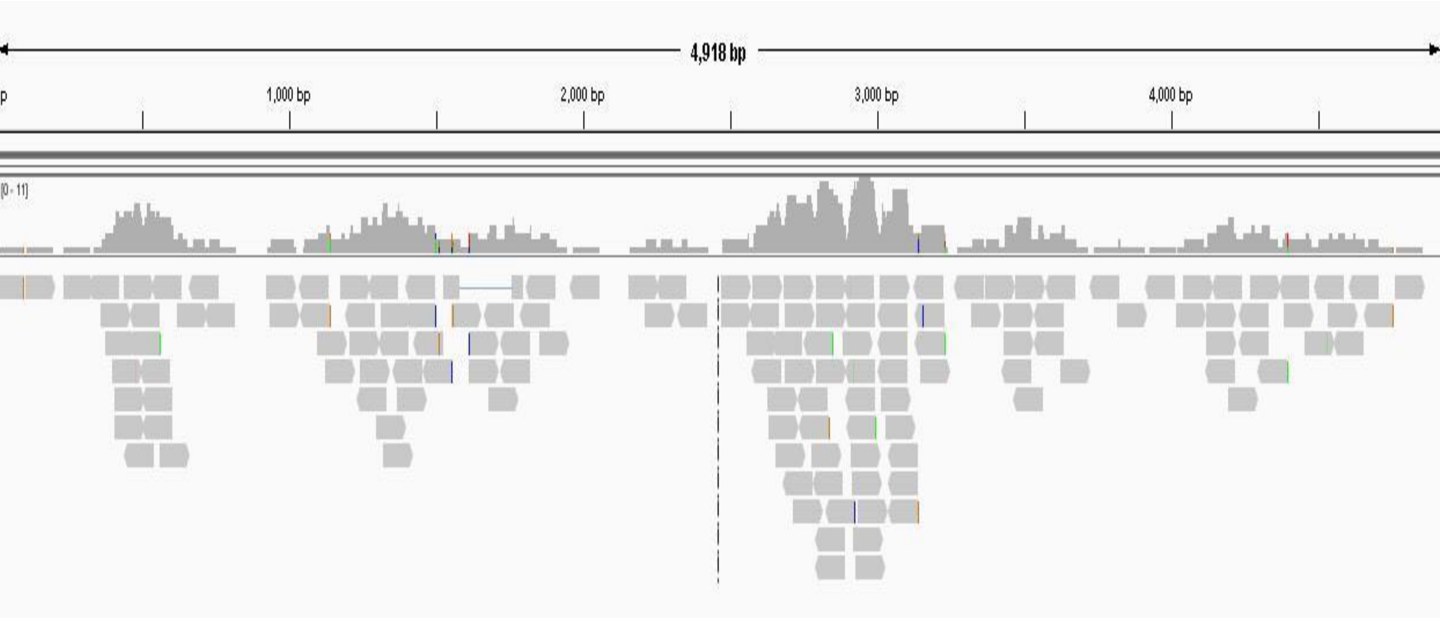

NCBI SRA database: SRX298075

Figure S5.

PbrAACA3_IT4

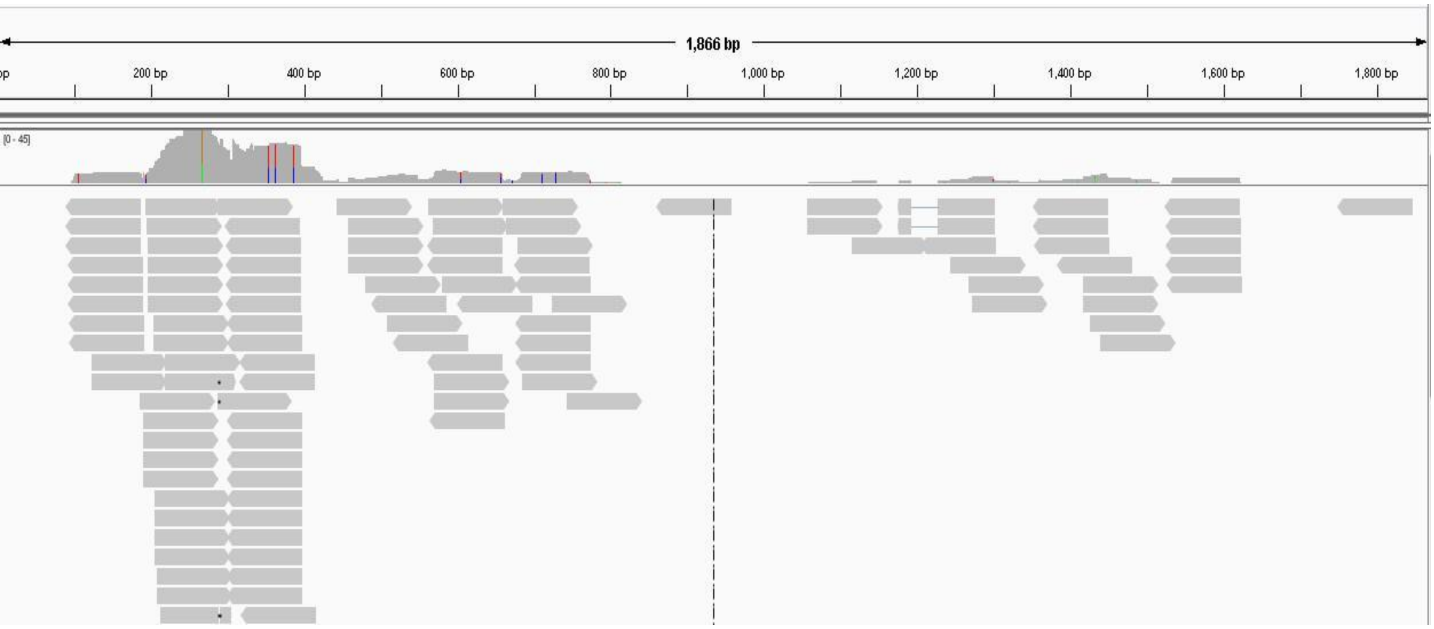

NCBI SRA database: SRX147917

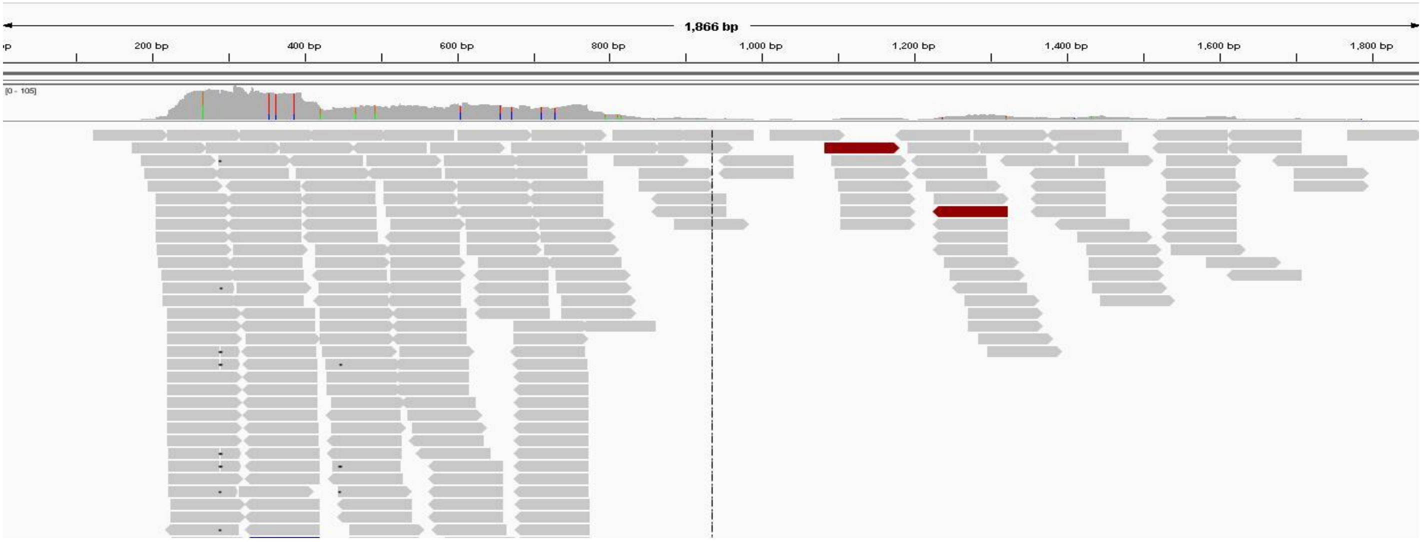

NCBI SRA database: : SRX298075

Figure S5.

PbrAACA4_IT11

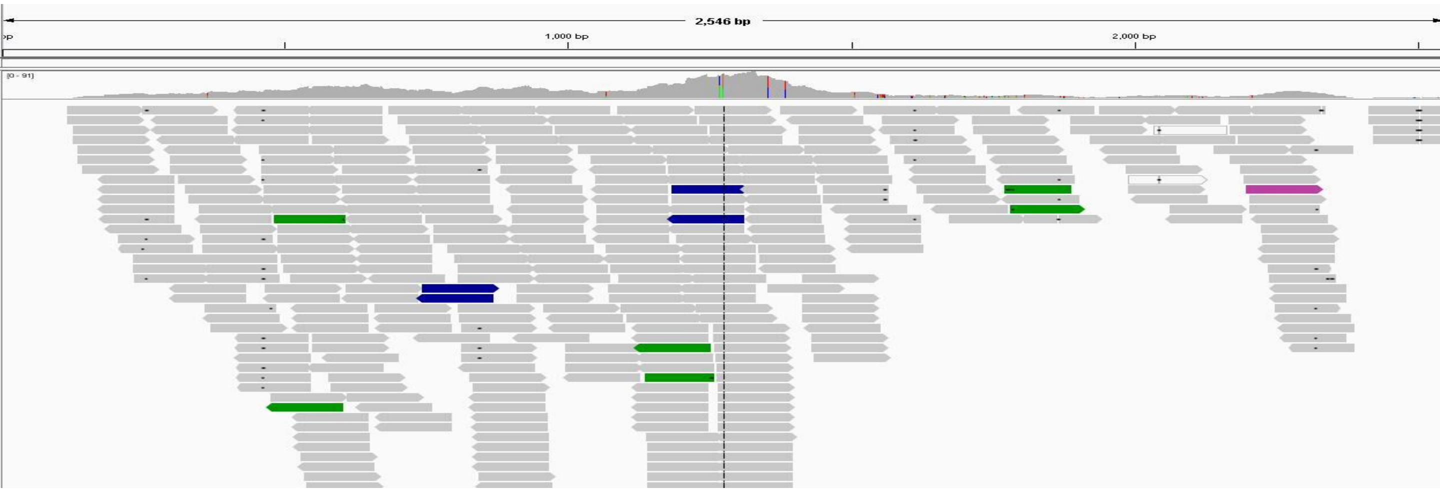

NCBI SRA database: SRX147917

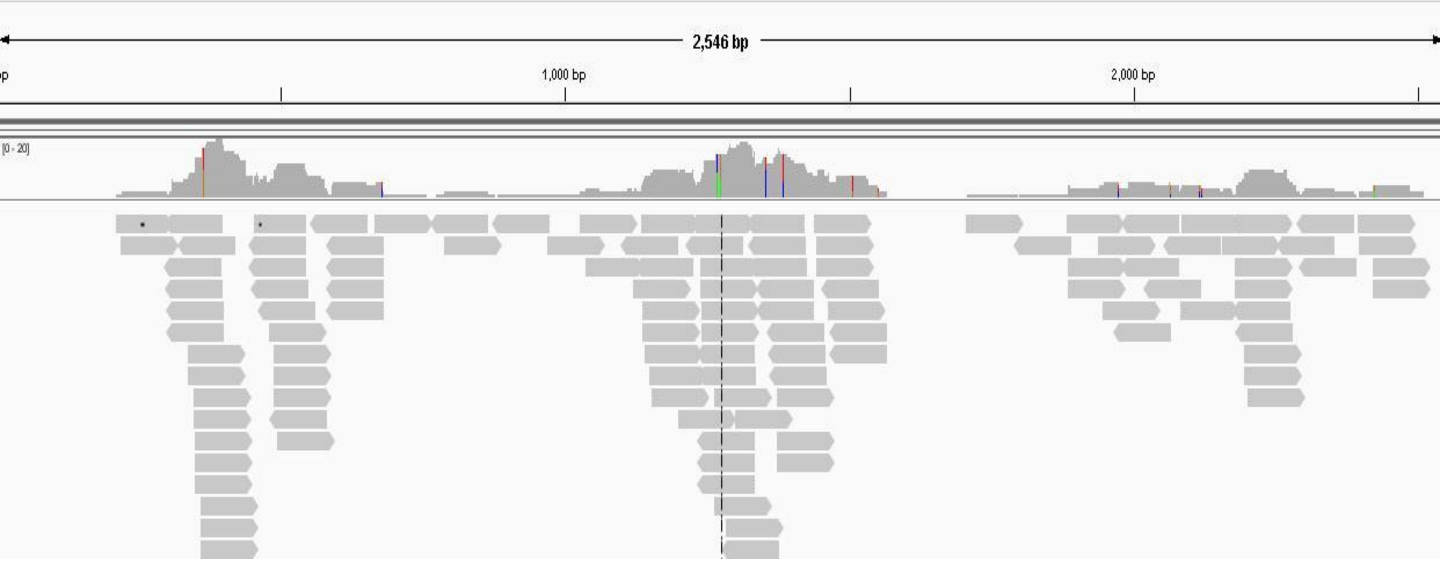

NCBI SRA database: SRX298075

Figure S5.

PbrAACA4_IT14

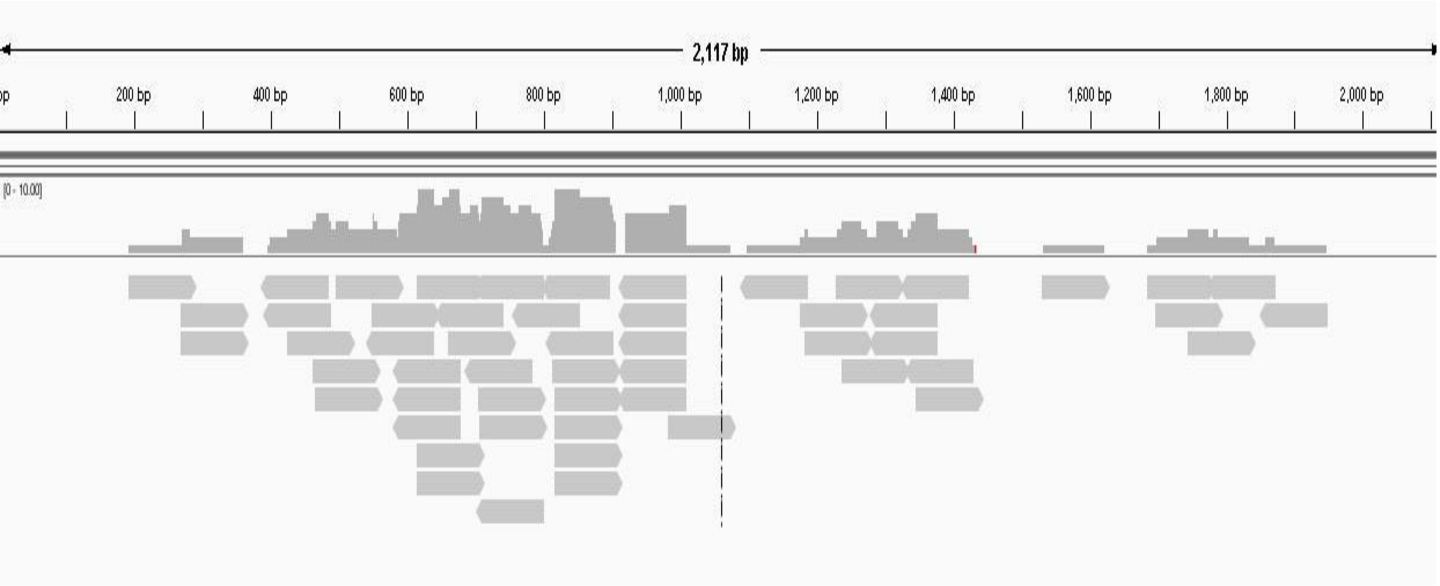

NCBI SRA database: SRX147917

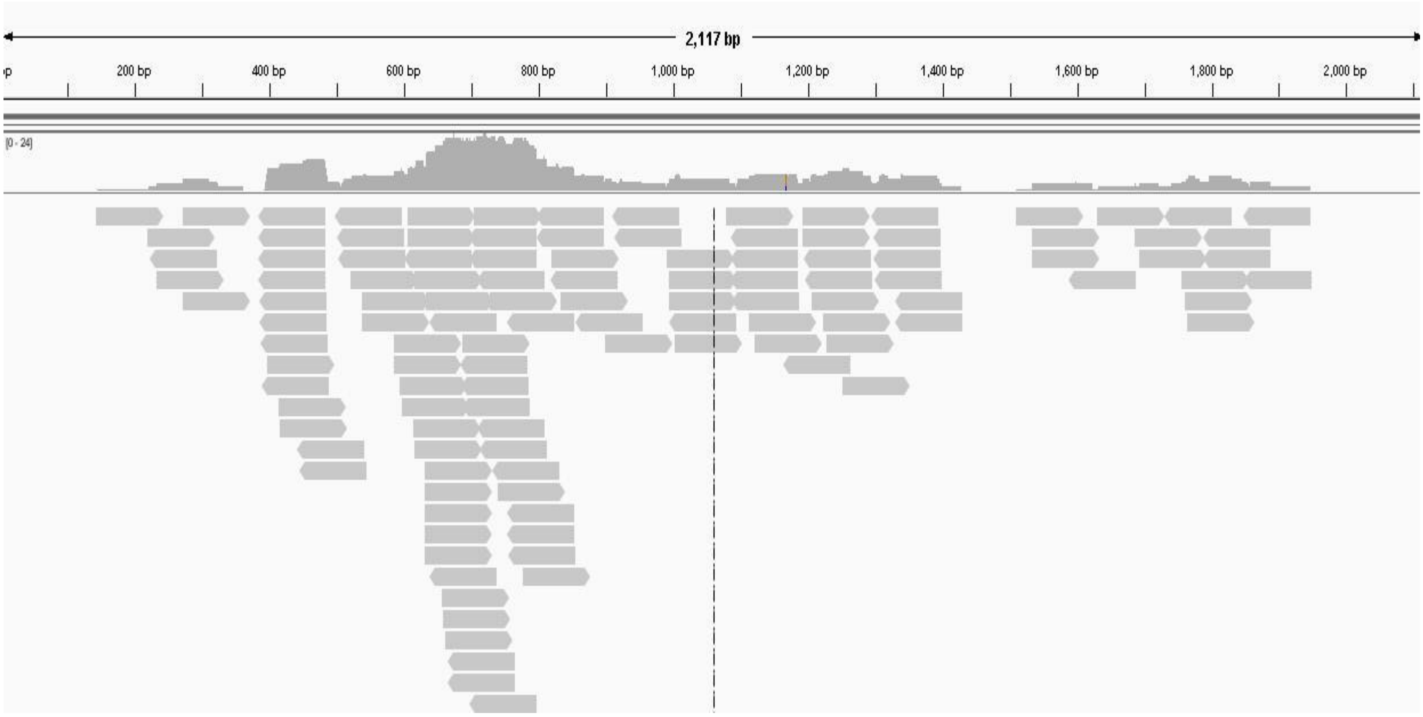

NCBI SRA database: SRX298075

Figure S5.

PbrAACA4_IT15

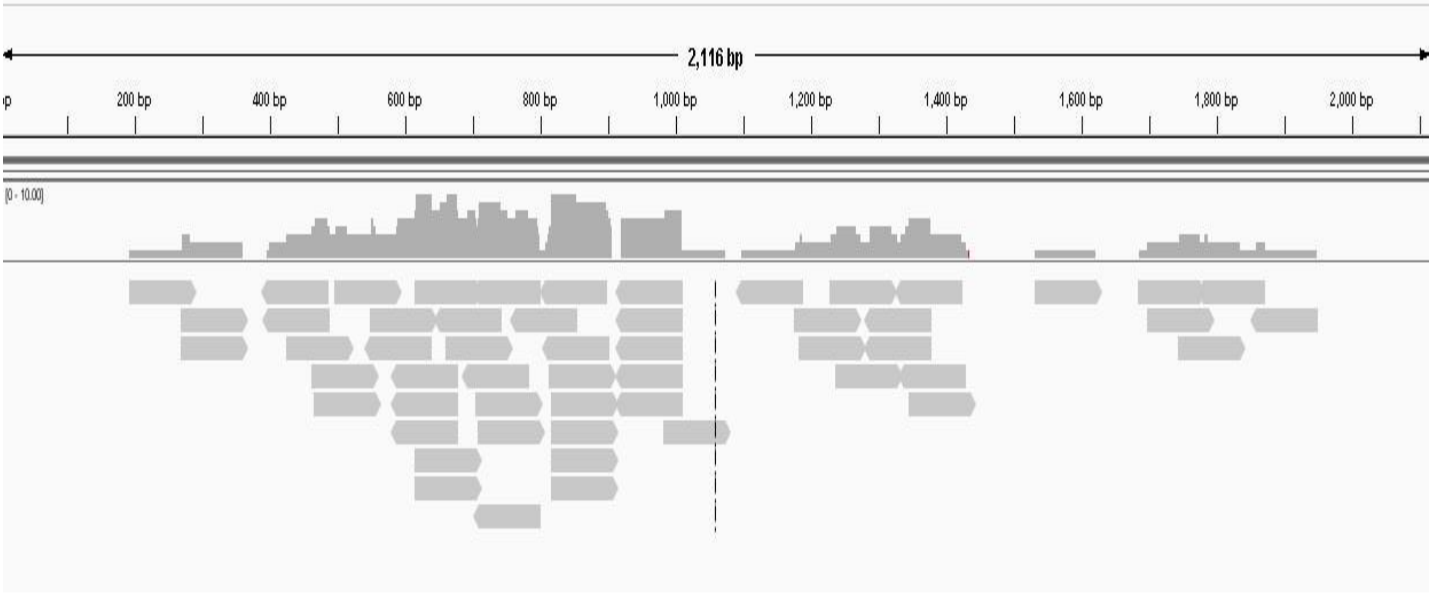

NCBI SRA database: SRX147917

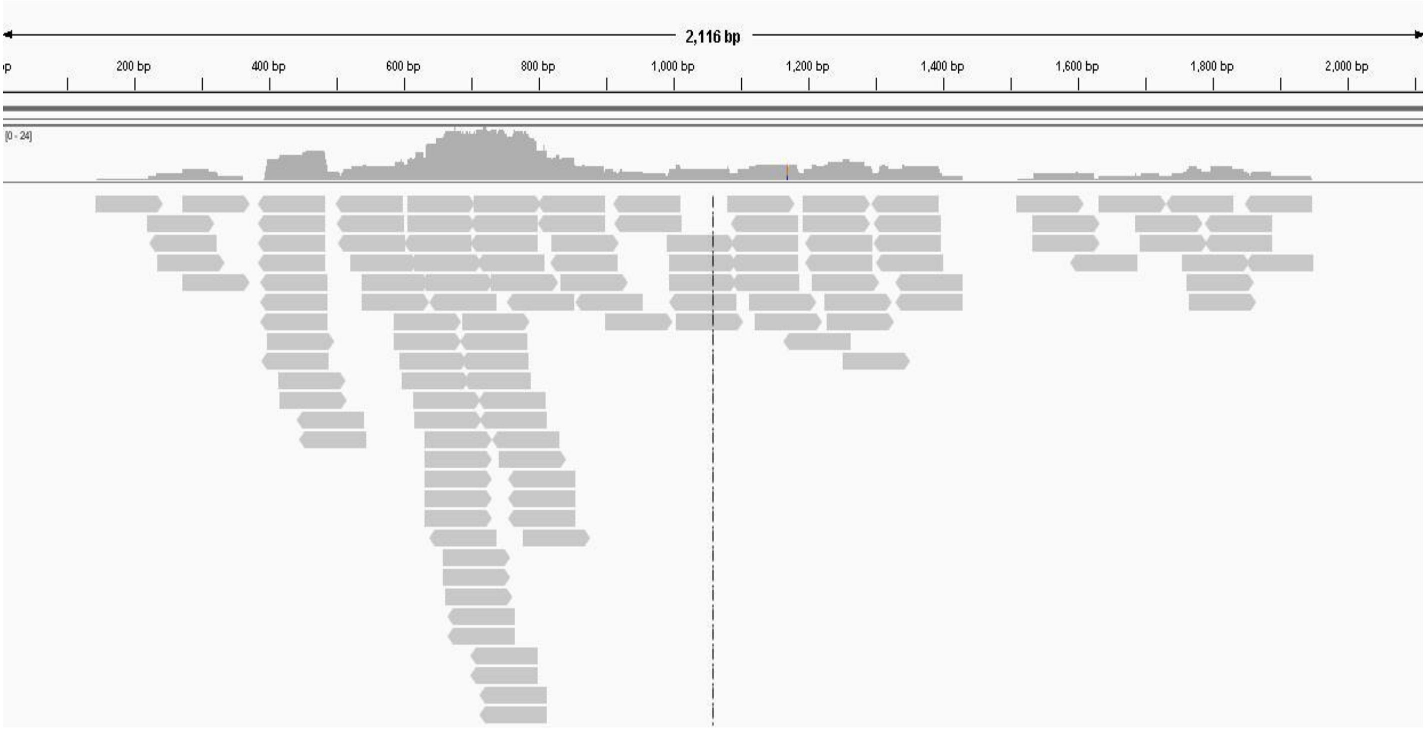

NCBI SRA database: SRX298075
